# Setting research priorities to improve global newborn health and prevent stillbirths by 2025

**DOI:** 10.7189/jogh.06.010508

**Published:** 2016-06

**Authors:** Sachiyo Yoshida, José Martines, Joy E Lawn, Stephen Wall, Joăo Paulo Souza, Igor Rudan, Simon Cousens, The neonatal health research priority setting group, Peter Aaby, Ishag Adam, Ramesh Kant Adhikari, Namasivayam Ambalavanan, Shams EI Arifeen, Dhana Raj Aryal, Sk Asiruddin, Abdullah Baqui, Aluisio JD Barros, Christine S Benn, Vineet Bhandari, Shinjini Bhatnagar, Sohinee Bhattacharya, Zulfiqar A Bhutta, Robert E Black, Hannah Blencowe, Carl Bose, Justin Brown, Christoph Bührer, Wally Carlo, Jose Guilherme Cecatti, Po–Yin Cheung, Robert Clark, Tim Colbourn, Agustin Conde–Agudelo, Erica Corbett, Andrew E Czeizel, Abhik Das, Louise Tina Day, Carolyn Deal, Ashok Deorari, Uğur Dilmen, Mike English, Cyril Engmann, Fabian Esamai, Caroline Fall, Donna M Ferriero, Peter Gisore, Tabish Hazir, Rosemary D Higgins, Caroline SE Homer, DE Hoque, Lorentz Irgens, MT Islam, Joseph de Graft–Johnson, Martias Alice Joshua, William Keenan, Soofia Khatoon, Helle Kieler, Michael S Kramer, Eve M Lackritz, Tina Lavender, Laurensia Lawintono, Richard Luhanga, David Marsh, Douglas McMillan, Patrick J McNamara, Ben Willem J Mol, Elizabeth Molyneux, G. K Mukasa, Miriam Mutabazi, Luis Carlos Nacul, Margaret Nakakeeto, Indira Narayanan, Bolajoko Olusanya, David Osrin, Vinod Paul, Christian Poets, Uma M Reddy, Mathuram Santosham, Rubayet Sayed, Natalia E Schlabritz–Loutsevitch, Nalini Singhal, Mary Alice Smith, Peter G Smith, Sajid Soofi, Catherine Y Spong, Shahin Sultana, Antoinette Tshefu, Frank van Bel, Lauren Vestewig Gray, Peter Waiswa, Wei Wang, Sarah LA Williams, Linda Wright, Anita Zaidi, Yanfeng Zhang, Nanbert Zhong, Isabel Zuniga, Rajiv Bahl

**Affiliations:** 1 Department of Maternal, Newborn, Child and Adolescent Health, World Health Organization, Geneva, Switzerland; 2 Centre for Intervention Science in Maternal and Child Health, Centre for International Health, University of Bergen, Norway; 3 London School of Hygiene and Tropical Medicine, London, UK; 4 Saving Newborn Lives, Save the Children, Washington, USA; 5 Department of Social Medicine, Ribeirăo Preto School of Medicine, University of Săo Paulo, Brazil; 6 Centre for Population Health Sciences and Global Health Academy, The University of Edinburgh Medical School, Scotland, UK; 7 Bandim Health Project, Indepth Network, Guinea–Bissau; 8 Faculty of Medicine, University of Khartoum, Sudan; 9 Kathmandu Medical College, Nepal; 10 Department of Pediatrics, University of Alabama at Birmingham, Birmingham, AL, USA; 11 Centre for Child and Adolescent Health, International Centre for Diarrhoeal Disease Research, Bangladesh; 12 Department of Neonatology Paropakar Maternity and women's Hospital, Nepal; 13 TRAction Bangladesh Project, University Research Co., LLC; 14 Johns Hopkins Bloomberg School of Public Health, USA; 15 Centro de Pesquisas Epidemiológicas, Universidade Federal de Pelotas, Brazil; 16 Research Center for Vitamins and Vaccines, Statens Serum Institut, and University of Southern Denmark/Odense University Hospital, Denmark; 17 Program in Perinatal Research, Yale University School of Medicine, USA; 18 Pediatric Biology Centre, Translational Health Science and Technology Institute, India; 19 University of Aberdeen, UK; 20 Center of Excellence in Women and Child Health, the Aga Khan University, Karachi, Pakistan; 21 Institute of International Programs, Johns Hopkins Bloomberg School of Public Health, USA; 22 The London School of Hygiene & Tropical Medicine, UK; 23 The University of North Carolina at Chapel Hill School of Medicine, USA; 24 Thrasher Research Fund, USA; 25 Department of Neonatology, Charité University Medical Center, Germany; 26 University of Alabama at Birmingham, USA; 27 Department of Obstetrics and Gynaecology, School of Medical Sciences, University of Campinas, Brazil; 28 Departments of Pediatrics, Pharmacology & Surgery, University of Alberta, Canada; 29 Charities, USA; 30 University College London Institute for Global Health, UK; 31 Perinatology Research Branch, Eunice Kennedy Shriver National Institute of Child Health and Human Development/National Institutes of Health/Department of Health and Human Services, Bethesda, Maryland and Detroit, Michigan, USA; 32 Independent consultant maternal health research, Rwanda; 33 Foundation for the Community Control of Hereditary Diseases, Hungary; 34 Biostatistics and Epidemiology, RTI International, USA; 35 LAMB Integrated Rural Health & Development, Bangladesh; 36 Division of Microbiology and Infectious Diseases, National Institute of Allergy and Infectious Diseases National Institute of Health, USA; 37 All India Institute of Medical Sciences, India; 38 Pediatrics and Neonatology, Yıldırım Beyazıt University Medical Faculty, Turkey; 39 Nuffield Department of Medicine & Department of Paediatrics, University of Oxford, UK and KEMRi–Wellcome Trust Research Programme, Nairobi, Kenya; 40 Newborn Health, Family Health Division, The Bill & Melinda Gates Foundation and the University of North Carolina Schools of Medicine and Public Health, USA; 41 Moi University, School of Medicine, Kenya; 42 International Paediatric Epidemiology; Affiliations: Medical Research Council Lifecourse Epidemiology Unit, University of Southampton, UK; 43 Department of Pediatrics, UCSF Benioff Children's Hospital, USA; 44 School of Medicine, Child Health and Pediatrics, Moi University, Kenya; 45 Children's Hospital, Pakistan Institute of Medical Sciences, Pakistan; 46 Eunice Kennedy Shriver NICHD Neonatal Research Network, Pregnancy and Perinatology, Branch, National Institute of Health, USA; 47 Centre for Midwifery, Child and Family Health, University of Technology, Sydney, Australia; 48 Centre for Child and Adolescent Health, International Centre for Diarrhoeal Disease Research, Bangladesh; 49 University of Bergen and Norwegian Institute of Public Health, Norway; 50 Japan International Cooperation Agency (JICA), Bangladesh; 51 Save the Children, USA; 52 Zomba Central Hospital, Ministry of Health, Malawi; 53 Saint Louis University, USA; 54 Paediatrics and Head of Department Shaheed Suhrawardy Medical College, Bangladesh; 55 Centre for Pharmacoepidemiology, Karolinska Institute, Sweden; 56 Departments of Pediatrics and of Epidemiology, Biostatistics and Occupational Health, McGill University Faculty of Medicine, Montreal, Quebec, Canada; 57 Global Alliance to Prevent Prematurity and Stillbirth (GAPPS), USA; 58 University of Manchester School of Nursing Midwifery & Social Work, University of Manchester, UK; 59 Indonesian Midwives Association, Indonesia; 60 Save the Children, Malawi; 61 Save the Children, USA; 62 Department of Paediatrics, Dalhousie University, Canada; 63 Departments of Paediatrics & Physiology, University of Toronto; Physiology & Experimental Medicine program, Hospital for Sick Children, Toronto, Canada; 64 Department of Obstetrics and Gynaecology, Academic Medical Centre Amsterdam, the Netherlands; 65 Paediatrics and Child Health, College of Medicine Malawi; 66 International Baby Food Action Network, Uganda; 67 STRIDES for Family Health, Management Sciences for Health, Uganda; 68 Faculty of Infectious and Tropical Diseases, London School of Hygiene and Tropical Medicine, UK; 69 Kampala Children's Hospital Limited and Childhealth Advocacy International, Uganda; 70 United States Agency for International Development /Maternal and Child Health Integrated Program, USA.; 71 Centre for Healthy Start Initiative, Nigeria; 72 Wellcome Trust Senior Research Fellow in Clinical Science, Institute for Global Health, University College London, UK; 73 All India Institute of Medical Sciences, India; 74 University of Tubingen, Germany; 75 Eunice Kennedy Shriver National Institute of Child Health and Human Development National Institutes of Health, USA; 76 Center for American Indian Health, Johns Hopkins Bloomberg School of Public Health, USA; 77 Save the Children, Bangladesh; 78 Department of Obstetrics and Gynecology, University of Tennessee Health Science Center, USA; 79 University of Calgary, Canada; 80 Environmental Health Science Department, University of Georgia, USA; 81 Tropical Epidemiology Group, London School of Hygiene & Tropical Medicine, London, UK; 82 Department of Pediatrics & Child Health, Women & Child Health Division, Aga Khan University, Pakistan; 83 Eunice Kennedy Shriver National Institute of Child Health and Human Development, National Institutes of Health, USA; 84 National Institute of Population Research and Training (NIPORT), Ministry of Health and Family Welfare, Bangladesh; 85 Kinshasa School of Public Health, School of Medicine, University of Kinshasa, Democratic Republic of Congo; 86 Department of Neonatology, University of Utrecht, the Netherlands; 87 Institute for Global Health Technologies Rice University, USA; 88 Division of Global Health, Karolinska Institutet, Sweden; 89 School of Medical Sciences, Edith Cowan University, Australia and School of Public Health, Capital Medical University, China; 90 Save the Children UK; 91 Eunice Kennedy Shriver National Institute of Child Health and Human Development National Institutes of Health, USA; 92 Aga Khan University, Pakistan; 93 Department of Integrated Early Childhood Development, Capital Institute of Paediatrics, China; 94 Developmental Genetics Laboratory, New York State Institute for Basic Research in Developmental Disabilities, USA; 95 Médecins sans Frontičres, Belgium

## Abstract

**Background:**

In 2013, an estimated 2.8 million newborns died and 2.7 million were stillborn. A much greater number suffer from long term impairment associated with preterm birth, intrauterine growth restriction, congenital anomalies, and perinatal or infectious causes. With the approaching deadline for the achievement of the Millennium Development Goals (MDGs) in 2015, there was a need to set the new research priorities on newborns and stillbirth with a focus not only on survival but also on health, growth and development. We therefore carried out a systematic exercise to set newborn health research priorities for 2013–2025.

**Methods:**

We used adapted Child Health and Nutrition Research Initiative (CHNRI) methods for this prioritization exercise. We identified and approached the 200 most productive researchers and 400 program experts, and 132 of them submitted research questions online. These were collated into a set of 205 research questions, sent for scoring to the 600 identified experts, and were assessed and scored by 91 experts.

**Results:**

Nine out of top ten identified priorities were in the domain of research on improving delivery of known interventions, with simplified neonatal resuscitation program and clinical algorithms and improved skills of community health workers leading the list. The top 10 priorities in the domain of development were led by ideas on improved Kangaroo Mother Care at community level, how to improve the accuracy of diagnosis by community health workers, and perinatal audits. The 10 leading priorities for discovery research focused on stable surfactant with novel modes of administration for preterm babies, ability to diagnose fetal distress and novel tocolytic agents to delay or stop preterm labour.

**Conclusion:**

These findings will assist both donors and researchers in supporting and conducting research to close the knowledge gaps for reducing neonatal mortality, morbidity and long term impairment. WHO, SNL and other partners will work to generate interest among key national stakeholders, governments, NGOs, and research institutes in these priorities, while encouraging research funders to support them. We will track research funding, relevant requests for proposals and trial registers to monitor if the priorities identified by this exercise are being addressed.

About 2.9 million newborns died in 2011, accounting for 44% of the world’s under-5 child deaths [1]. The proportion of neonatal mortality continues to increase because the neonatal mortality rate is declining at a slower rate than the mortality rates for older children [1]. Moreover, 2.7 million stillbirths occur each year, at least 40% of which occur during labour [2]. The leading killers of newborns are preterm birth complications, intrapartum–related events and neonatal infections such as pneumonia, sepsis or meningitis [3]. A high proportion of stillbirths, neonatal and also maternal deaths happen at birth and during the first days after birth – a total of over 3 million deaths [4]. This is also a critical time window to address acute morbidity and long–term impairment associated with preterm birth, intrauterine growth restriction (IUGR), congenital abnormalities, and perinatal or infectious insults [5,6].

With the approaching deadline for the achievement of the Millennium Development Goals (MDGs) in 2015, and the creation of new framework for development goals [7], there is an increasing need to guide the limited research capacity and funding to obtain the maximum impact on maternal and child health. Hence the World Health Organization (WHO) has initiated a set of global research priority–setting exercises in 2007–2008 for improving health of mothers, newborns, children and adolescents [8–12]. The five–year evaluation of that exercise from the perspective of donors, policy–makers and researchers is currently under way and it is showing an increased focus on identified research priorities from all three groups of stakeholders – in terms of investments by the donors [13,14], initiatives launched by policy–makers [15–19] and publication output from researchers [2,20–23], respectively. As part of this initiative, the Department of Maternal, Newborn, Child and Adolescent Health undertook this exercise for setting research priorities in newborn health and stillbirth, in collaboration with Saving Newborn Lives (SNL), a program of Save The Children. The time frame for the expected impact of the research extends to 2025 to allow for medium term and long–term research investments to also be considered. Alongside the persisting urgency of reducing mortality and the findings from previous research priority exercises the group believed that the research should also address morbidity, development, and long–term sequelae of preterm birth, small for gestational age as well as other hypoxic or infectious insults in the neonatal period (**Box 1**). In the exercise, we focused on intrapartum stillbirth as a high proportion of stillbirths occurs during the laboure.

Box 1 **The purpose and remit of this research priority setting exercise**

**Population of interest:**

Newborns and stillbirths, survival and health, preterm birth, growth and impairment–free development

**Time frame:**

2013–2025, reaching beyond the timeframe of the Millennium Development Goals

**Research domains:**

DISCOVERY (new interventions)

DEVELOPMENT (improved interventions)

DELIVERY (implementation of existing interventions)

(note: not including description eg, epidemiology)

**Audience (stakeholders):**

Governments, researchers in low and middle–income countries, international donors

## METHODS

A working group that managed the agenda–setting process consisted of staff responsible for newborn health in WHO and Saving Newborn Lives. The group defined the scope of the priority setting exercise (**Box 1**). Methodology developed by the Child Health and Nutrition Research Initiative (CHNRI) was adapted and used for this priority setting exercise, to enable systematic listing and transparent scoring of many competing research questions [24–26]. This methodology had been used in the previous priority setting exercises by the WHO on five major causes of child deaths: pneumonia, diarrhea, preterm birth and low birth weight, neonatal infections, and birth asphyxia [8–12]. The previous exercise coordinated by the WHO was sharply focused on short–term gains, ie, within the MDG4 target of the year 2015. In addition, the CHNRI methodology has been used by many other subject groups and multiple organizations [27–33]. **Box 2** shows the steps we followed during this priority setting process.

Box 2 **Adapted Child Health and Nutrition Research Initiative's (CHNRI) methodology applied to set newborn research priorities**

*1.* Selection of individuals to submit ideas and to score questions:

Individuals representing a wide range of technical expertise in the area of newborn health and birth outcomes were selected by including

• Top 100 most productive researchers in the previous 5 years (2008–2012), according to the Web of Science®, in any research that involved neonates anywhere in the world, including (but not limited to) fundamental research, obstetrics and gynaecology, social science, and other fields;

• Top 50 most productive researchers in the previous 5 years (see above) in research specifically involving neonates in low and middle income countries (LMICs);

• Top 50 most productive researchers in the previous 5 years (see above) in any research involving stillbirths;

• 400 program experts in newborn health, who were contacted through the Healthy Newborn Network Database, representing mainly national–level health programme managers in LMICs.

2. Identification of questions to be scored:

All the identified individuals were approached and asked to submit their three most promising ideas for improving newborn health outcomes by 2025. An expert group meeting was convened to review the 396 questions received from 132 experts. After removing or merging seemingly duplicate ideas, the submissions were consolidated into a set of 205 research questions and clarity of the questions was improved.

3. Scoring of research questions:

A set of 5 criteria to assess the proposed 205 research questions was agreed on.

The scoring criteria were based on CHNRI methodology [8–12]

i. Likelihood of answering the question in an ethical way

ii. Likelihood of efficacy

iii. Likelihood of deliverability and acceptability

iv. Likelihood for an important disease burden reduction

v. Predicted effect on equity

During the preliminary meeting, 14 experts invited from the larger pool of responders completed their scoring to test the methodology. The remaining experts were asked independently to answer a set of questions via an online survey on all the chosen criteria for all listed research options. Scores from a total of 91 experts were received.

4. Computation of scores for competing research options and ranking:

The intermediate scores were computed for each of the five criteria and they could potentially range between 0–100%. Those scores indicate the “collective optimism” of the group of scorers that a given research question would fulfil each given criterion. The overall research priority score for each research question was then computed as the mean of the intermediate scores. The average expert agreement scores were also calculated (**Online Supplementary Document(Online Supplementary Document)**).

A large group of researchers and program experts were identified and asked to submit three ideas for improving newborn health outcomes by 2025 (**Box 2**). Two hundred of the most productive researchers, representing a broad range of technical expertise and regional diversity, identified through Web of Science® ranking tools, were invited by email to propose research questions on newborn health and birth outcomes. A further 400 program experts in newborn health programmes were also invited to propose research questions.

The proposed research questions and scoring criteria were refined by a small group of 14 experts who were invited by the WHO to participate in a two–day workshop. Each question was assigned to a domain and a technical area. The first of the three domains was “discovery”, which included research aimed at finding new solutions such as new medicines, vaccines or other preventive interventions, or new diagnostics. The second domain was “development”, which included research questions aimed at improving existing interventions, reducing their costs or making them simpler to deliver. The third domain was “delivery”, which included research questions that would help deliver existing interventions to more mothers and newborns with high quality. The five separate technical areas included: (i) preterm birth; (ii) intrapartum–related events including intrapartum stillbirths; (iii) newborn infections; (iv) congenital malformations and other specific conditions; and (v) integrated care including the care for mothers and neonates;

The final list of research questions and scoring criteria were sent to the original group of 600 experts with an invitation to score them. Each research question was assessed by the expert and received a score of 1.0, 0.5 or 0 for five preset criteria, with the option of not assigning any score in case the expert did not feel confident to decide on that criterion. Scoring took place over eight weeks and was conducted and returned to the coordinators at the WHO by 91 experts.

Intermediate scores for each research question against the 5 criteria were computed as the sum of the scores for that particular criterion divided by the total number of scorers. This resulted in a number between 0–100% that captured the “collective optimism” of the group of 91 scorers that a given research question would fulfill each given criterion. The overall research priority score (RPS) for each research question was then computed as the mean of the intermediate scores calculated for each of the five criteria: RPS = [(Criterion 1 score %)+(Criterion 2 score %)+(Criterion 3 score %)+(Criterion 4 score %)+(Criterion 5 score %)]/5. The confidence interval was calculated using the bootstrapping methods in STATA version 11.2.

## RESULTS

**Figure 1:**
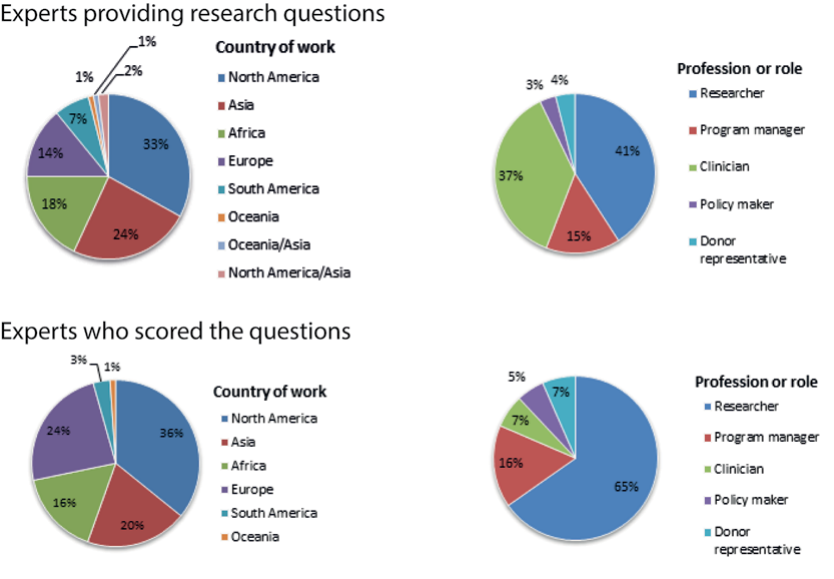
Background characteristics of 132 experts who provided questions and 91 experts who scored the questions.

In total, 132 of the 600 invited experts proposed a total of 396 research questions, which were then checked for similarity and consolidated in a final list of 205 questions to be scored. The characteristics of respondents are summarized in **Figure 1**. The 205 research questions were then scored by 91 experts. About 40% of the scorers were based in low and middle income countries (LMICs) in Africa, Asia, and South America. About two–thirds (65%) worked in academic or research institutions and the remainder was divided between program managers (16%), clinicians (7%), donor representatives (7%) and policy makers (5%) (**Figure 1**).

The overall research priority scores given to the 205 proposed questions ranged from 90% (high) to 47% (low; full list of scored questions is presented in the **Online Supplementary Document(Online Supplementary Document)**). The level of agreement between the 91 experts ranged from 77% (high) to 34% (low), suggesting that on average, for each research question of interest, between three–quarters and one–third of the scorers were in agreement in their responses to each criterion.

The overall scores for the highest priority questions ranged from 79% to 90% (**Table 1**). Agreement scores indicated that more than two thirds of the experts had a common view towards the list of research priorities. Nine of the ten top priorities were in the domain of “delivery”, with simplified neonatal resuscitation programs and clinical algorithms and improved skills of community health workers leading the list. Among the 11 priorities shown in this table, three addressed preterm birth, four addressed intrapartum–related events and four addressed newborn infections.

**Table 1:** Top ten research priorities for improving newborn health and birth outcomes by 2025 as ranked by 91 experts

Rank	Research questions	Domain	Total score (confidence interval)	Agreement between scorers	Answerable?	Efficacy?	Deliverability?	Impact?	Equity?
1	Can simplified neonatal resuscitation program delivered by trained health workers reduce neonatal deaths due to perinatal asphyxia?	Delivery	90 (85–91)	77	96	91	94	77	92
2	How can the health worker's skills in preventing and managing asphyxia be scaled up?	Delivery	88 (83–89)	74	96	91	89	75	86
3	Can simple clinical algorithms used by CHW identify and refer neonates with signs of infection and consequently reduce newborn mortality?	Delivery	86 (83–89)	72	92	92	92	66	88
4	How can exclusive breastfeeding in low–resource contexts be promoted to reduce neonatal infections and mortality?	Delivery	85 (79–89)	72	94	89	86	69	86
5	Can the training of CHWs in basic newborn resuscitation reduce morbidity and mortality due to perinatal asphyxia?	Delivery	83 (78–86)	67	94	84	84	64	88
6	How can the administration of injectable antibiotics at home and first level facilities to newborn with signs of sepsis be scaled up to reduce neonatal mortality?	Delivery	82 (78–86)	64	89	88	88	59	84
7	Can community–based initiation of Kangaroo Mother Care reduce neonatal mortality of clinically stable preterm and low birth weight babies?	Development	80 (74–84)	66	86	87	81	69	77
8	How can facility based initiation of Kangaroo Mother Care or continuous skin–to–skin contact be scaled up?	Delivery	80 (71–84)	62	90	82	84	62	81
9	How can chlorhexidine application to the cord be scaled up in facility births and in low NMR setting to reduce neonatal infections and neonatal mortality?	Delivery	80 (70–83)	67	91	85	89	52	81
10	How can quality of care during labour and birth be improved to reduce intrapartum stillbirths, neonatal mortality and disability?	Delivery	79 (71–82)	65	83	84	82	72	75
11*	Can community based “extra care” for preterm/LBW babies delivered by CHWs reduce neonatal morbidity and mortality in settings with poor accessibility to facility care?	Delivery	79 (70–82)	63	87	87	81	62	81

*The overall and criterion specific scores ranged from 0% to 100%. The 11^th^ question added to complete the list of top 10 priorities in the domain of “delivery”. The question originally ranked 5^th^ was omitted from this table because it was a variant of question that already received a higher overall score.

In the domain of “development”, the top 10 priorities (**Table 2**) were ranked between 8^th^ and 50^th^ on the list of all research questions (displayed in full in **Online Supplementary Document(Online Supplementary Document)**). They were led by ideas on improved Kangaroo Mother Care, improve accuracy of diagnosis by community health workers, and perinatal audits. Two priorities among the leading ten in this domain were identified in each of the areas of preterm birth, intrapartum related events and newborn infections, while the remaining 4 priorities related to integrated care.

**Table 2:** Top ten development research priorities for improving newborn health and birth outcomes by 2025 as ranked by 91 experts

Rank	Research questions	Total score (confidence interval)	Agreement between scorers
8*	Can community–based initiation of Kangaroo Mother Care reduce neonatal mortality of clinically stable preterm and low birth weight babies?	82 (78–86)	64
26	How can the accuracy of community health workers in detecting key most important high risk conditions or danger signs in pregnant women be improved?	77 (70–80)	61
35	Can perinatal audits improve quality of care in health facilities and improve fetal and neonatal outcomes?	74 (67–79)	58
37	Can intrapartum monitoring to enhance timely referral improve fetal and neonatal outcomes?	74 (67–79)	57
38	Can training community health workers to recognize and treat neonatal sepsis at home with oral antibiotics when referral is not possible reduce neonatal mortality?	74 (62–78)	57
40	Can oral amoxicillin at home for treatment of neonatal pneumonia reduce neonatal mortality?	73 (64–78)	58
43	Can models for strengthening capacity of health Professionals in caring for neonates in peripheral hospitals improve neonatal outcomes?	73 (63–77)	54
44	Can intervention package for CHWs to prevent and manage perinatal asphyxia be delivered by community health workers?	72 (64–77)	55
47	Can low–cost devices for facility care of newborns be developed and tested for the effectiveness at various levels of the health system (eg, CPAP devices, syringe drivers, IV giving sets, phototherapy units, oxygen concentrators, oxygen saturation monitors incubators, ventilators, therapeutic hypothermia technology) ?	72 (65–76)	53
50	Can surfactant reduce preterm morbidity and mortality in low and middle income countries?	72 (65–78)	56

*Also in the overall top 10 priorities.

The 10 leading priorities for discovery research (**Table 3**) ranked between 58^th^ and 129^th^ on the list of all research questions (see **Online Supplementary Document(Online Supplementary Document)**) and they focused on stable surfactant with novel modes of administration, ability to diagnose fetal distress and novel tocolytic agents. Agreement scores for the ten leading questions ranged from 42% to 49%. Three priorities were identified in each of the areas of preterm birth and newborn infections, two on preventing intrauterine growth restriction and one each on intrapartum–related events and antepartum stillbirths.

**Table 3:** Top ten discovery research priorities in discovery for improving newborn health and birth outcomes by 2025 as ranked by 91 experts

Rank	Research questions	Total score (confidence interval)	Agreement between scorers
55	Can stable surfactant with simpler novel modes of administration increase the use and availability of surfactant for preterm babies at risk of respiratory distress syndrome?	71 (62–73)	49
71	Can the method to diagnose fetal distress in labour be more accurate and affordable?	66 (57–71)	49
97	Can strategies for prevention and treatment of intrauterine growth restriction be developed?	64 (51–68)	46
105	Can novel tocolytic agents to delay or stop preterm labour be developed in order to reduce neonatal mortality and morbidity?	63 (54–68)	42
116	Can major causal pathways and risk factors for antepartum stillbirth be identified?	61 (52–66)	43
118	Can novel point of care diagnostics for congenital syphilis be identified in low resource setting to improve management?	60 (53–64)	49
120	Can novel antibiotic or other biological agents be identified?	60 (51–65)	40
121	Can the new method identify intrauterine growth restriction at the early stage (including biomarkers) and predict abnormal postnatal growth and body composition?	60 (52–63)	43
125	Can novel vaccines for maternal immunization be developed and evaluated to prevent newborn infections (eg, GBS, Klebsiella, *E coli*, Staph)?	60 (51–64)	41
129	Can preterm birth be delayed or averted with antioxidant and/or nutrient supplementation (eg, Vitamin D, omega–3 fatty acids)?	58 (48–63)	42

GBS – group B streptococcus, Staph – staphylococcus

There was a remarkable similarity in the scoring pattern between experts from a research background and those from a program background for the top 10 ranked priorities (**Table 4**). The programme experts had a tendency to assign somewhat higher overall scores to “delivery” questions, which was mediated through their higher scoring of maximum potential impact and equity criteria. Among “development” questions, the scorers with a background in research gave higher scores for efficacy and deliverability, while programme experts gave higher scores for impact and equity criteria. Surprisingly, the scoring pattern of both groups of experts for “discovery” questions was very similar, both for overall score and for each of the 5 criteria.

**Table 4:** Overall scoring pattern by profile of experts

	Median (IQR)		
	**All scorers (n = 91)**	**Researchers (n = 61)**	**Programme experts (n = 30)**
**TOTAL SCORE**			
Delivery	82 (80–86)	83 (78–86)	86 (81–87)
Development	74 (72–74)	75 (71–76)	75 (68–79)
Discovery	61 (59–64)	62 (60–62)	63 (58–65)
**AGREEMENT**			
Delivery	67 (65–72)	68 (64–73)	70 (65–75)
Development	57 (55–58)	58 (56–60)	55 (54–62)
Discovery	43 (42–49)	45 (42–47)	44 (39–49)
**ANSWERABLE?**			
Delivery	92 (87–94)	92 (88–95)	91 (90–94)
Development	84 (82–89)	87 (81–90)	84 (78–89)
Discovery	76 (73–78)	76 (74–79)	76 (70–79)
**EFFICACY?**			
Delivery	87 (84–91)	87 (83–91)	88 (84–90)
Development	81 (77–83)	84 (79–84)	78 (76–81)
Discovery	68 (64–70)	68 (65–72)	69 (59–72)
**DELIVERABILITY?**			
Delivery	85 (82–89)	86 (82–91)	87 (82–89)
Development	77 (75–80)	79 (77–81)	74 (70–84)
Discovery	68 (66–72)	69 (64–72)	70 (64–72)
**IMPACT?**			
Delivery	68 (62–72)	65 (58–70)	73 (69–80)
Development	56 (53–57)	53 (52–58)	62 (52–65)
Discovery	46 (39–50)	46 (38–48)	44 (36–54)
**EQUITY?**			
Delivery	84 (81–88)	84 (76–89)	87 (79–88)
Development	74 (66–77)	71 (65–76)	76 (75–80)
Discovery	54 (50–59)	52 (50–58)	53 (50–65)

## DISCUSSION

In this paper, we present global research priorities that have the potential to impact mortality, morbidity, child development, and long–term health outcomes among neonates in the period between 2013–2025. Despite the broad focus on these outcomes and a 12–year timeline, “delivery” questions received highest scores, followed by “development” and “discovery” questions, as was the case in previous exercises with shorter time lines focusing only on reducing mortality [8–12].

The major emerging themes in the domain of “delivery” included simplifying intervention delivery to implementation at lower levels of the health system, evaluating delivery of interventions by community health workers, developing strategies to improve quality of care during labour and childbirth, and addressing barriers in the scaling up of high impact interventions. It is interesting to note that 5 of the questions were related to neonatal resuscitation. This could be related to neonatal resuscitation being the most dramatic intervention in newborn care. The major themes in the domain of “development” were adapting known interventions to make them deliverable at the community level, adapting effective interventions to increase deliverability in health facilities in low and middle income countries, and approaches such as perinatal audits to improve quality of care to mothers and newborns. The themes in the domain of “discovery” included new, more effective and less expensive medicines for preventing preterm birth and treating sepsis, point of care diagnostics for infections, maternal vaccines to prevent newborn infections, and basic science work on causal pathways for identifying intervention targets and biomarkers for preterm birth, IUGR, and antepartum stillbirths. It is noteworthy that preterm prevention was not ranked highly, even though it may have the largest impact. This appears to be the result of these questions being scored low in answerability.

The relatively lower scores for the “development” and “discovery” groups of research questions may have several possible explanations. First, more than 95% of the neonatal deaths occur in low and middle–income countries (LMICs). Therefore, research addressing neonatal health issues that are relatively more important in wealthy countries may be perceived to contribute less to global reduction in mortality and morbidity, explaining some of the lower scores received by potentially promising research on novel interventions based on high technologies. Second, “discovery” research often takes longer to be translated into measurable benefits in terms of mortality burden reduction, and by definition the link to reduction in mortality and inequity is less direct. One specific example is research on prevention of preterm birth – while it was likely to have high impact, it was ranked only 129^th^ among the 205 questions. Thereby, respondents sent a message that this research question would likely be difficult to answer given the current stage of knowledge. Third, the process of delivery of novel interventions usually requires specific funding mechanisms, such as PEPFAR or Advance Market Commitment (AMC), which require time for a political agreement [34,35].

The CHNRI process we followed for setting priorities has several strengths. The methodology is transparent, replicable, and feasible to apply via e–mail [8–12, 27–33]. The output is intuitive and easily understood, and it has been refined and improved through many exercises over the past several years [36]. In this particular exercise, further improvements have been introduced to the process. We chose a large number of experts based on their productivity in the previous five years using Web of Science®, thus transparently identifying the group that was most likely to understand the field and its present research challenges and gaps. A very wide global network of programme experts in the Saving Newborn Lives’ Network was also invited. Moreover, we used online data collection tools, such as Survey Monkey® and Google Analytics®, which allowed monitoring of the progress of the exercise in real time, ensured adequate representation of experts by their background and region, and increased the efficiency of data management. Finally, 132 experts proposed research questions and 91 scored all the questions in this exercise; this is considerably more then in previous priority setting exercises using CHNRI methodology, where we typically involved fewer scorers, research ideas, and criteria scored by each expert.

There may be concern that the results derived from the CHNRI approach might represent only the collective opinion of the limited group of people who were included in the process. However, we were able to obtain questions and scores from a large number of experts worldwide, who were selected in a transparent and replicable manner, based on their research productivity in the field. The large number of participants and the protection against potential bias provided by the CHNRI approach make our results more credible, although it remains apparent that the highest scored questions may still be biased towards those that researchers are most familiar with and so may bias reflect research already in progress. This issue may be particularly relevant in view that only about a quarter of originally invited researchers, policy makers and programme experts eventually contributed to generating research questions, and only about one in six completed the scoring process, making response bias an important potential concern. Second, even though the list of proposed questions was reviewed and refined before sending for scoring, there were still overlaps in some research questions, possibly creating confusion in scoring such questions. Those and other possible strengths and limitations of CHNRI methodology are described and discussed in greater detail in **Online Supplementary Document(Online Supplementary Document)**.

A recent analysis of funding committed globally to improving neonatal health and birth outcomes has shown that donor mention of the “newborn” has increased quite sharply since 2005. However, given a total of only 10% of all donor aid to RMNCH mentioning the word “newborn”, and only 0.01% referring to interventions expected to reduce newborn deaths, it still seems unlikely that donor aid is commensurate with the large burden of 3.0 million newborn deaths each year, or with the burden of morbidity, developmental and long–term health outcomes [37]. The word “stillbirth” occurred only twice in the OECD database between 2002 and 2010, suggesting even lower attention for the world’s 2.7 million stillbirths.

Large inequities in current research funding support exist not only in the amounts invested in newborn health in comparison to other diseases globally, but also between different neonatal conditions themselves. Conditions that affect newborns in high–income countries receive more funding and attention than conditions that largely affect newborns in low–income countries. For instance, the research on care of preterm babies in neonatal intensive care units has received considerably more funding over the past several years in comparison to intrapartum–related birth outcomes or newborn sepsis [38].

The results presented in this paper will assist both the donors and the researchers in setting evidence based priorities to address the key gaps in knowledge, that could make the most difference in saving newborn lives and preventing stillbirth. In addition, attention to many of these questions could also improve maternal and child health outcomes. Likewise, research priorities to address other related areas such as maternal, child and adolescent health and health system issues may have substantial effect on newborn health. Complementary exercises are under way to identify research priorities in these areas. Using the identified research priorities, WHO, SNL and other partners, that are linked to the Every Newborn action plan launched in 2014 [39], will work to generate research interests among key national stakeholders, governments, NGOs, and research institutes, while encouraging research funders to support these priorities. We will track research funding, relevant request for proposals and trial registers to monitor if the priorities identified by this exercise are being addressed, and highlight those that are not being addressed.
