# Supplementary material for: Setting research priorities to improve global newborn health and prevent stillbirths by 2025
Source: J Glob Health. 2015 Aug 30;6(1):010508. doi: 10.7189/jogh.06.010508 (PMC4576458; doi:10.7189/jogh.06.010508)
Supplement: Online Supplementary Document [file jogh-06-010508-s001.pdf]

Online Supplementary Document

Yoshida et al. Setting research priorities to improve global newborn health and prevent stillbirths by 2025

J Glob Health 2016;6:010508

Supplementary online material

S1. Identifying technical experts who were invited to participate in the CHNRI exercise

The following Web of Science® search criteria were used to identify Group 1 technical experts:

(neonatal OR newborn* OR neonate*) AND document type (Article) AND year published (2008-2012). In total, 39,377 names were found and the top 100 were selected.

For Group 2 technical experts, the same criteria were used with the addition of all the list of countries fall under the category of low and middle-income countries (LMIC). In total, 3,775 names were identified and the top 50 were selected.

For the Group 3 technical experts, we conducted a search using the following criteria:

(stillbirth*) AND document type (Article) AND year published (2008-2012). Of the 1,494 names returned in our search, the top 50 were selected.

The 400 program experts were identified through databases maintained by Save the Children, Saving Newborn Lives (SNL). These experts were carefully chosen based on their experience in newborn health related programs in LMICs. Efforts were made to achieve a strong representation of government, UN and program experts working in LMICs. Each expert was only included once in either the most productive researcher or program expert group.

S2. Characteristics of the expert group contributing the research questions

Among the 600 invited experts, a total of 132 agreed to participate in the CHNRI exercise. The background characteristics are described in the main paper.

S3. Compiling and refining questions

A total of 394 research questions were submitted online. About half of the questions were related to intervention delivery, and a quarter to the domains of development and discovery, respectively. All questions were reviewed and edited, if necessary, by seven technical experts (SY, RB, JL, IR, SW, JPS, JM). During the process, efforts were made to maintain the sense of the original questions. A total of 41 questions were deleted because they were merely descriptive, unclear, too broad, too narrow, similar to another question, or already included among research outcomes that have been addressed in previous studies.

Finally, 14 technical experts chosen from a pool of responders were invited to join 7 members of the management group for a two-day meeting in September 2012 to review and revise the questions. The meeting participants were divided into four groups, where they reviewed, edited to clarify, added any missing research questions, and/or deleted repetitive questions. The technical areas assigned to each group were on: (i)

neonatal infections, congenital abnormalities and non-classified questions; (ii) integrated care across maternal newborn and child health; (iii) “birth asphyxia” and stillbirth-related questions; and (iv) preterm birth. This process resulted in a total of 205 refined questions. Day 2 of the meeting was mainly devoted to finalizing the questions and the scoring criteria. After an agreement was given to the scoring criteria, 14 participants were asked to score the questions. Details of scoring criteria are provided in the main text.

S4. Characteristics of the expert group scoring the research questions

Among the 600 invited experts contacted, a total of 78 experts participated in the scoring exercise. The background characteristics are described in the main paper.

S5. The CHNRI methodology for setting priorities in health research investments.

STAGE 1: Defining the context and criteria for priority setting
Specifying the context a priori is a critical part of the CHNRI process, because priority scores for many research investment options may change substantially according to different contexts. The context for this exercise was defined to address research investment priorities beyond MDG4, i.e. by the year 2025.<br><br>The context was specified by the management group as follows:<br><br>• Burden of disease of interest: mortality, morbidity, birth outcomes, early development, short-term and long-term health outcomes;<br><br>• Population of interest: newborns (until 28 days);<br><br>• Existing policy/target: improving newborn health and birth outcomes globally;<br><br>• Level of urgency: high (because the relative importance of newborn deaths among child deaths is continuously increasing)<br><br>• Time frame: to achieve detectable improvement in newborn health and birth outcomes by 2025;
**STAGE 2: Choice of technical experts, systematic listing and scoring of research investment options**<br><br>The process of the selection of the experts is described in the main paper and Supplementary online material (**Sections S1-S4**). The first task of the technical experts was to propose a large spectrum of research questions in a systematic way, belonging to three broad research themes: “Delivery”, “Development” and “Discovery”. We did not invite research ideas on “Description”, because we agreed that over the past several years most of the research issues from that domain have been addressed satisfactorily and gained global acceptance. The management group based at the WHO collected all the proposed ideas from 132 responders (of 600 invited) independently by e-mail. The process was open-ended and it initially yielded about 400 research questions. Then the list of research questions was consolidated and narrowed down to a manageable size for the scorers – i.e. 205 questions. During the pilot exercise, the management group limited the overlap between proposed ideas and ensured that the research questions were phrased in a way that would make the expected new knowledge apparent. We feel that the final list of 205 questions covers the wide spectrum of all possible questions.<br><br>The second task for the experts was to score all research questions independently, according to the five agreed criteria (presented in **Box 1**). For each of the 205 research questions and each criterion, each expert answered a questions targeted to assess the likelihood of the proposed research to comply with the priority-setting criterion (see **Box 1**). This task was completed by all experts. The entire process was conducted and completed via e-mail between October and December 2012. Further information on methods related to this part of the

priority-setting process were presented elsewhere in greater detail.[1,2]

STAGE 3: Community involvement - input from larger group of stakeholders

CHNRI methodology ensures community involvement through incorporating the opinions and values from a broader group of stakeholders (e.g. expected recipients of the research, taxpayers who fund health research, health workers, journalists and media, experts in ethics, law, political science, etc.).[1] Stakeholders lack expertise to directly decide research priorities, but their opinions and values can still be incorporated by weighing the chosen priority-setting criteria according to their perceived importance. In this exercise, we decided not to apply weights provided by the group of stakeholders most appropriate to this exercise (see **Methods** section in the main text). The reason for this is that this exercise is so broad, general and far-reaching, that it would be difficult to identify an appropriate group of stakeholders that would have authority to represent all possible stakeholders. Instead, we presented the scores as they were computed, and allowed different possible users of the results of this exercise to apply weights and thresholds that would reflect their own views and values after the exercise is completed. Those weights and thresholds could be applied to intermediate scores, which we provided in all the tables with results, to adjust the overall priority scores and the ranking list. More detailed explanations on the rationale and methods for including stakeholders' opinions in the process are presented elsewhere (**Kapiriri L**).

STAGE 4: Computation of "research priority scores"

For each of the 205 research ideas, all the experts answered the questions listed in **Box 1** by 'Yes' (1 point) or 'No' (0 points). They were also allowed to declare an informed but undecided answer (0.5 points) or declare themselves insufficiently informed to answer the question (missing input). Thus, the proposed research questions received intermediate scores for each of the five criteria as "the proportion of maximum possible points scored when an answer was given" (i.e., excluding the missing input). Intermediate scores represent a direct measure of collective optimism of the 92scorers. Each of the 205 listed research questions received five intermediate scores (each ranging between 0-100%). The overall research priority score (RPS) was then computed as the mean of all five intermediate priority scores. The final list of research priorities with intermediate scores and overall research priority scores for all 205 proposed research questions is presented in **Supplementary online material - S6**. The exact scores given to all 205 research questions by individual experts to generate the ranking list are presented in **Supplementary online material – S7**.

STAGE 5: Assessment of agreement between scorers

CHNRI methodology has the ability to expose the issues of greatest agreement and controversy. This allows more focused discussion among experts following this exercise, and informs the investors and policy makers about the amount of controversy that surrounds each research question. The datasets that CHNRI methodology produces are not amenable to the usual Kappa agreement statistics, which has been discussed in detail elsewhere1.

For each evaluated research investment option, AEA is informing us, for an average question, what proportion of scorers gave the same most frequent answer. This parameter satisfactorily accounts for missing answers, is unaffected by responses of 'undecided', and is also unaffected by the varying number of scorers per criterion and differences in scorer composition for the different criteria.

STAGE 6: Assessing the advantages and limitations of the CHNRI methodology

The applied CHNRI methodology proved to be helpful to systematically list and score a very large number of specific research questions, as shown recently in exercises conducted at national level in South Africa, and at global level for mental health research issues, childhood pneumonia, childhood diarrhoea, neonatal infections, "birth asphyxia", primary health care, disability groups, etc.[3-9] Other advantages of the CHNRI process include

its systematic nature, transparency, well defined (a priori) context and criteria chosen for discriminating between research investment options, a highly structured way in which relevant information is obtained from the scorers, independent scoring that limits influence of strong-minded individuals on the rest of the scorers, its informative and intuitive quantitative outputs and ability to expose points of greatest agreement and controversy.

Still, the methodology is not free of several possible biases. Although the advantages mentioned above represent a serious attempt to deal with many issues inherent to a highly complex process of research priority setting, there are still concerns over the validity of the CHNRI approach and related biases. One of them is related to the fact many possible good ideas (“research questions”) may not have been included in the final list of proposed research questions that was scored by the experts. There is also a potential bias towards items that get the greatest media coverage. The spectrum of research questions listed initially in this exercise was derived through a systematic process, but it is not endless and it cannot cover every single research idea. Specific research methodologies (i.e. randomized clinical trials, etc.) are not mentioned because the research questions listed in that exercise are unlikely to be answered by a single well-defined study. Therefore, the CHNRI process aims to achieve reasonable coverage of the spectrum of possible ideas. After the completion of the exercise, approximate scores and ranks for some specific research questions that are missing in the initial systematic list could still be estimated – either by relating them to the most similar questions on the list, or by having those missed questions scored by a single expert (or by a group), using the CHNRI framework and then comparing the computed score to all other scores received for the originally listed research options.

Another concern over the CHNRI process is that its end product represents a possibly biased opinion of a very limited group of involved people. In theory, a chosen group of experts can have biased views in comparison to any other potential groups of experts. However, the number of people globally who possess enough experience, expertise and knowledge on this issue to be able to judge a very diverse spectrum of research questions is rather limited (although certainly much larger than the group that we eventually selected). If one thinks of this “global pool of technical experts” as the whole population that could theoretically be used to solicit expert opinion on the questions that need to be asked, we then selected a “sample” from that population, based on their track record in research on newborn health and birth outcomes. Given that the “sample” of the experts chosen for this exercise (92 persons) was the largest and the most diverse to ever conduct a CHNRI exercise, while the number of experts in this neglected health problem globally is not large, we doubt that there would be considerable differences in the composition of the initial list of questions (or results of the scoring process) if some other group of experts had been selected.

Apparently, CHNRI methodology is not free of bias that results from the choice of the experts, and different groups of experts may indeed have quite different opinions. However, the larger and more diverse the group of chosen experts (as in this case), the less likely is that the results of their scoring would significantly deviate from the output of any other large and diverse expert group, chosen from a limited “pool of global technical experts on newborns”.

STAGE 7: Validation of the CHNRI methodology

Fundamentally, the CHNRI methodology combines two ideas:

(i) “Principal component analysis” - a statistical technique which reduces a very complex system of large number of variables to a smaller number of relatively independent “principal components” which still capture a sizeable proportion of variation in the system; by defining a set of 5 “criteria”, CHNRI process effectively reduces a notoriously complex and multi-dimensional task of priority setting, which could be approached through an almost infinite number of “lenses”, into an exercise where the 5 most important (and reasonably independent) criteria for priority setting are clearly defined. They can even be weighted afterwards, in order of

their importance to the users.

(ii) “The wisdom of crowds” – this refers to the process of taking into account collective opinion of a group of individuals rather than a single expert (or small number of experts) to answer a question, because it has been shown that the average of collective guesses are nearly always closer to the truth than any expert judgement. The pre-requisites for this process to work are: (i) Diversity of opinion (each person should have private information even if it's just an eccentric interpretation of the known facts); (ii) Independence (people's opinions aren't determined by the opinions of those around them); (iii) Decentralization (people are able to specialize and draw on local knowledge); and (iv) Aggregation (some mechanism exists for turning private judgments into a collective decision – in this case, the CHNRI method).

The validation of the CHNRI method based on the exercises conducted to date showed: (i) extraordinary stability (correlation coefficients of over 90%) of scores given to same questions by the same experts in different points in time; (ii) almost identical scores of the same question scored by a larger group multiple times (score always falls within +1.7 points on a scale 0-100); and (iii) Monte Carlo simulations in random sub-samples of the larger group of scorers showed that the probability that the outcomes of the exercise could be substantially different if another group of experts conducted the scoring becomes incredibly small as soon as each criterion is scored by more than 17-23 rational persons with some knowledge of the problem; (iv) change of the context of the exercise leads the same group of experts to assign significantly different scores to the same research questions.

In this paper, we used 92 technical experts to score each criterion. Thus, given the well-defined context for this CHNRI exercise and a set of simple YES/NO questions, it is entirely improbable that any other group of rational individuals with some knowledge of the problem, no matter of their background or selection, would ever reach dramatically different conclusions than our group did.

Although this may seem counter-intuitive to some critics, this is the basic property of the „wisdom of crowds“ phenomenon (for more details please see an excellent book by James Surowiecki: The Wisdom of Crowds: Why the Many Are Smarter Than the Few and How Collective Wisdom Shapes Business, Economies, Societies and Nations), which CHNRI uses as its fundamental principle. Once that each individual gets a right to express judgement that is treated equally as the judgement of any other individual, then the personal biases that those individuals bring into the process tend to cancel and dilute each other regardless who the participants are. What is left is an information based on accumulated knowledge, lifetime experience and common sense of those who took part – which is the result of the CHNRI process.

In comparison to other methods for setting priorities, in “expert panel”-type processes one very loud vote has a potential to heavily bias the process, resulting in shameful inequity and snowballing support for some issues at the expense of the others, a situation which we are observing today. We recently conducted Delphi and CHNRI exercises in parallel to compare them. This happened during the large GAPPS meeting (“Global action plan for prematurity and stillbirth”) sponsored by The Gates Foundation. Nine working groups were defining priorities using Delphi-type process, while three working groups were using CHNRI method. At the end of the conference, the rapporteurs from Delphi groups realised that it was simply unfeasible to have a discussion on all possible research options and keep in mind all their pros and cons at all times. Eventually, the group leaders ended up forwarding the ideas which they originally brought to the table and gained support for them from the rest of the group. In CHNRI groups, however, a process highlighted pros and cons of many competing ideas. More importantly, after the scoring was conducted, the top priorities were often surprising to the group - because they were sometimes the issues which have not been discussed at all, and few experts would have highlighted them as the leading priorities.

S6. An in-depth analysis of the reasons for divergence in overall research priority scores received by the similar research ideas.

After the scoring was completed, we focused on several pairs (or triplets) of questions from the initial list that had similarities or overlap in their content, but received different overall scores. Those examples were particularly informative to understand what makes a research question more or less attractive as a research priority for the scorers. In this panel, we discuss several examples that are informative about the nature of the received scores and their sensitivity to the way the question is formulated. **Supplementary table S8** highlights twelve pairs and three triplets of questions which have clear similarities in content, but also some differences in the way that the question was asked, that eventually led to different scores for particular criteria. We considered the difference in score for individual criterion by 5.0 points worth discussing.

Example 1. The questions ranked 15 and 39 both suggested delivery research to improve postnatal care in the community, but while the first one was more general, the second one specifically mentioned traditional birth attendants as providers (TBA). Because of this, the latter question received lower scores for deliverability and potential impact, revealing the concerns of the scorers towards the capacity of TBAs to be engaged at larger scale and make a true difference.

Example 2. The questions ranked 43 and 59 both suggested development research to increase capacity of health professionals at the facility level to improve neonatal outcomes; the first one did not specify the modes of training, while the second one suggested interned-based and mobile phone-based approaches. Because of this, the second question scored higher on answerability – presumably because it was easier for the scorers to envisage the mode of training; however, it scored lower on impact and equity, because the scorers didn't see those specific tools as potentially most feasible in low-resource settings.

Example 3. The questions ranked 10 and 61 both proposed delivery research to improve scale up of Kangaroo mother care for preterm and low birth weight newborns; the first was relevant to all contexts, while the second implied access to facilities and proposed studying long-term outcomes such as school performance. Because of those two differences, the latter question received less collective optimism towards effectiveness, deliverability, potential impact and the effect on equity.

Example 4. The questions ranked 12 and 23 both suggested to evaluate effectiveness and cost of "follow-up" care ("extra care") for preterm and low birth weight newborns; the first one was focused on the community level, and the second one on newborns that were discharged from a facility. As the basic underlying idea is the same, and it is delivered in community in both questions, the scores hardly differ – apart from the equity criterion, where it is clear that the latter research would only benefit newborns with access to facilities.

Example 5. The questions ranked 72 and 92 both suggested research to improve quality of facility-based newborn care; the first one suggested doing this at the referral-level facility, and the second one at the district hospital facility. Both questions received very similar scores, but the former one was considered more answerable than the latter one.

Example 6. The questions ranked 1 and 9 both suggested delivery research to simplify neonatal resuscitation program to reduce perinatal asphyxia; the first offered to study the role of education, implementation, quality improvement and system changes, while the latter one suggested a specific intervention of making bags and masks accessible to trained birth attendants. The first question out-performed all other suggested research questions, while the second one was also ranked very highly; however, it was inferior in terms of answerability, efficacy, deliverability and potential impact, but it did better in its predicted effect of equity – presumably because the intervention is particularly suitable for low-resource settings.

Example 7. The questions ranked 38 and 40 both suggested evaluating effectiveness and cost of training community health workers to treat neonatal sepsis in the community with oral antibiotics – the only difference

was that the first question suggested 3rd generation cephalosporins and the second one suggested amoxicillin. This difference was clearly insufficient for the scorers to provide a notably different score against any of the criteria – although amoxicillin was considered slightly more deliverable, presumably because it is more widely available.

Example 8. The questions ranked 73 and 87 both suggested nutritional interventions to prevent congenital disorders, but the first specified folic acid and iodine, while the latter didn't mention any specific nutritional intervention. Clearly, the only difference was in the likelihood of efficacy related to proposed research; the first question had mentioned two specific intervention of proven efficacy, so it scored higher against that criterion.

Supplementary table S8 offers several further examples where it becomes more apparent where did the differences in research priority scores between the 205 research questions arise from. In **Tables 1-3** in the main text, we decided to omit research questions from these similar pairs of questions that received the lower score, in order to promote more ideas at the top of the rankings, rather than presenting the same idea with a slight modification twice. This is indicated by the asterisk in the column with overall research priority scores and a footnote.

S7. Full list of 205 research priorities ranked by overall research priority score.

RANK	RESEARCH QUESTIONS	DOMAIN	TOTAL SCORE	CONFIDENCE INTERVAL			AGREEMENT BETWEEN SCORERS	ANSWERABLE?	EFFICACY?	DELIVERABILITY?	IMPACT?	EQUITY?
1	Simplified neonatal resuscitation program delivered by trained health worker in reducing neonatal deaths due to perinatal asphyxia	Delivery	90	85	-	91	77	96	91	94	77	92
2	Approaches to scale up worker skills in preventing and managing asphyxia	Delivery	88	83	-	89	74	96	91	89	75	86
3	Simple clinical algorithms to identify and refer neonates with signs of infection by CHW and consequently reduce newborn mortality	Delivery	86	83	-	89	72	92	92	92	66	88
4	Promote exclusive breastfeeding in low-resource contexts to reduce neonatal infections and mortality	Delivery	85	79	-	89	72	94	89	86	69	86
5	Training of CHWs in basic newborn resuscitation in reducing morbidity and mortality due to perinatal asphyxia	Delivery	83	78	-	86	67	94	84	84	64	88
6	Community-based initiation of Kangaroo Mother Care to reduce neonatal mortality of clinically stable preterm and low birth weight babies	Development	82	78	-	86	64	89	88	88	59	84
7	Scale up administration of injectable antibiotics at home and first level facilities to newborns with signs of sepsis to reduce neonatal mortality	Delivery	80	74	-	84	66	86	87	81	69	77
8	Facility based initiation of Kangaroo Mother Care or continuous skin-to-skin contact	Delivery	80	71	-	84	62	90	82	84	62	81

9	Impact of chlorhexidine application to the cord in facility births and in low NMR setting to reduce neonatal infections and neonatal mortality	Delivery	80	70	-	83	67	91	85	89	52	81
10	Quality of care during labour and birth in reducing intrapartum stillbirths and reduce neonatal mortality and disability	Delivery	79	71	-	82	65	83	84	82	72	75
11	Evaluate the effectiveness and cost of "extra care" for preterm/LBW babies at community level by trained community health workers including identification, additional home visits, supportive referral etc. in reducing neonatal morbidity and mortality in settings with poor accessibility to facility care	Delivery	79	70	-	82	63	87	87	81	62	81
12	Evaluate the effectiveness and cost of including newborn care as part of integrated community case management (ICCM) programs in improving newborn survival	Delivery	79	70	-	83	57	90	85	82	61	77
13	Evaluate approaches to improve community based postnatal care (e.g. home visits) within the context of existing health system	Delivery	79	71	-	81	63	86	82	85	59	82
14	Evaluate the effectiveness and cost of parental education on practices to prevent newborn infections at home, including recognition of neonatal infection and care seeking for it	Delivery	79	70	-	82	64	85	81	85	60	81
15	Develop and evaluate the effectiveness and cost of a minimum package of essential interventions and commodities that must be available at every birth to ensure safety of babies	Delivery	79	72	-	84	63	82	82	82	66	80
16	Evaluate the effectiveness and cost of approaches (eg notification systems) to improve early postnatal home visit coverage (in the first 24 hours of life) in odrer to improve maternal and newborn outcomes	Delivery	78	69	-	82	62	90	84	84	51	84
17	Evaluate the effectiveness and cost of community midwives or other 'skilled' community-based cadres to reduce overall neonatal mortality in settings	Delivery	78	72	-	82	64	86	88	82	56	80

	where facilities are commonly overcrowded or inaccessible											
18	Evaluate effectiveness and cost of provision of clean delivery kits and capacity building of traditional birth attendants in reducing incidence of neonatal infections	Delivery	78	71	-	83	66	85	80	84	56	85
19	Evaluate effectiveness, cost, safety and sustainability of provision of antenatal corticosteroid for reducing preterm deaths at lower levels of the health system either pre-referral or where referral is not possible	Delivery	78	72	-	83	60	91	83	80	60	75
20	Evaluate the effectiveness and cost of approaches to increase coverage of clean delivery practices in facilities	Delivery	78	71	-	80	63	86	83	89	64	66
21	Develop and evaluate the effectiveness and cost of a package of community follow-up care for preterm and low birth weight newborns after discharge from a health facility	Delivery	77	69	-	81	60	88	87	82	58	73
22	Evaluate innovations (e.g. mobile technology) that would enable community based workers to reach newborns promptly after birth for assessment of danger signs and referral	Delivery	77	70	-	81	63	93	83	80	56	74
23	Design and evaluate different approaches for training to standardize and sustain skills in identification and case management of newborns with infections	Delivery	77	70	-	81	60	89	84	81	62	69
24	Evaluate the accuracy of community health workers in detecting a limited number of high risk conditions / danger signs in pregnant women that require delivery at a health facility	Develop ment	77	70	-	80	61	91	83	81	53	77
25	Evaluate the effectiveness, cost and sustainability of conditional cash transfers in increasing coverage of antenatal care, skilled birth attendance and postnatal care	Delivery	77	67	-	79	52	87	85	76	57	78

26	Evaluate approaches to scale up effective community-based participatory approaches to promote maternal and newborn health (through increased awareness, preventive health strategies, home-based monitoring and improved care-seeking)	Delivery	76	69	-	84	59	84	84	78	57	78
27	Evaluate approaches to improve quality of facility-based intrapartum and immediate/early postnatal care for mothers and newborns (e.g., audit, “drills,” intrapartum and pre-discharge checklists)	Delivery	76	68	-	80	60	88	85	86	59	63
28	Identify and address the main barriers for implementation of programmes to prevent mother to child transmission of HIV in different low-resource settings	Delivery	76	70	-	80	63	88	85	83	47	77
29	Compare the effectiveness and cost of improving families' illness recognition and care-seeking for newborn illness versus active home visits in increasing the coverage of treatment for sick newborns	Delivery	75	68	-	79	59	87	76	81	54	79
30	Evaluate the effectiveness of a program in improving pregnancy outcomes through reducing the time between identification of a danger sign and arrival at a health facility e.g. using mobile technology, establishing a district-wide transport plan, and coordinating an appropriate referral response	Delivery	75	67	-	81	60	84	82	72	59	79
31	Identify and address the main barriers for implementation of programmes to promote use of impregnated bednets in different low-resource settings to reduce the burden of congenital malaria, stillbirth, preterm and low birth weight	Delivery	75	68	-	79	59	86	86	85	45	72
32	Identify the components of the referral mechanism for women requiring emergency obstetric care which have a key impact on perinatal outcomes (For example mode of transport, woman's position during transfer, alertness of the receiving facility before her arrival, accompaniment by health professional)	Delivery	75	64	-	78	58	90	77	78	55	73

33	Evaluate effectiveness and cost of perinatal audits to improve quality of care in health facilities and improve fetal and neonatal outcomes	Develop ment	74	67	-	79	58	90	83	80	53	63
34	Design and evaluate a "First 1000 day" nutritional package (preconception, pregnancy, and first two years after birth) targeted for the poorest families to increase maternal weight and the linear growth of the infants through 2 years of age.	Delivery	74	66	-	77	59	85	79	70	53	81
35	Develop and evaluate simple, cost effective strategies for intrapartum monitoring to enhance timely referral to improve fetal and neonatal outcomes	Develop ment	74	67	-	79	57	88	83	76	56	66
36	Evaluate the effectiveness and cost of training community health workers to recognize and treat neonatal sepsis at home with oral antibiotics (such as third generation cephalosporins) when referral is not possible, on neonatal mortality.	Develop ment	74	62	-	78	57	85	77	75	54	77
37	Evaluate the effectiveness of Traditional Birth Attendants carrying out early postnatal home visits for newborns who were born at home in improving newborn health outcomes	Delivery	73	66	-	78	59	85	78	74	48	80
38	Evaluate effectiveness of oral amoxicillin at home, with supervised home visits, for treatment of neonatal pneumonia	Develop ment	73	64	-	78	58	82	76	80	52	76
39	Evaluate the effectiveness and cost of various approaches (direct observation, mannequins, scenarios with hyper-local videos, scenarios alone, among others) for sustaining newborn resuscitation skills of CHWs	Delivery	73	65	-	77	55	87	79	79	50	69
40	Evaluate the effectiveness and cost of using mobile technology (mHealth) to improve quality of care of mothers and newborn babies at peripheral centres and in the community	Delivery	73	66	-	77	57	88	78	76	51	70

41	Develop and evaluate the impact of models for strengthening capacity of health professionals in caring for neonates in peripheral hospitals and lower level health facilities on improving neonatal outcomes	Develop ment	73	63	-	77	54	79	79	77	57	72
42	Evaluate effectiveness and cost of an intervention package to prevent and manage perinatal asphyxia delivered by community health workers to reduce newborn mortality and morbidity	Develop ment	72	64	-	77	55	81	74	75	56	75
43	Evaluate the effectiveness and cost of approaches to increase coverage of clean delivery practices in homes	Delivery	72	66	-	79	54	78	73	78	57	76
44	Develop low-cost devices for facility care of newborns (e.g. CPAP, syringe drivers, IV giving sets, phototherapy, oxygen condensors, saturation monitoring, incubators, ventilators, therapeutic hypothermia technology) and test their effectiveness at various levels of the health system	Develop ment	72	65	-	76	53	82	78	73	61	67
45	Evaluate the effectiveness and cost of community health workers or TBAs to facilitate referral and increase families' compliance with referral	Delivery	72	63	-	75	56	80	76	75	52	78
46	Evaluate behavioural change communication approaches to promote messages on early initiation of breastfeeding and exclusive breastfeeding up to 180 days	Delivery	72	61	-	76	57	83	75	76	53	73
47	Evaluate the effectiveness and costs of surfactant in reducing preterm morbidity and mortality in low and middle income countries	Develop ment	72	65	-	78	56	83	83	75	56	63
48	Evaluate effectiveness and cost of using community health workers to act as the first link in the referral chain connecting women in labour to the formal health care system in increasing skilled attendance and reducing intrapartum stillbirths and deaths/disability due to perinatal asphyxia	Delivery	72	64	-	75	55	83	73	70	57	77

49	Develop and evaluate effectiveness of needle free antibiotics delivery to treat neonatal sepsis	Develop ment	72	64	-	76	53	88	77	80	49	64
50	Develop and evaluate strategies to increase correct use of partograph, linked to appropriate action	Delivery	71	63	-	76	49	82	81	80	51	63
51	Evaluate the effectiveness, cost and safety of CPAP to decrease neonatal mortality and morbidity in preterm infants in health care facilities in low resource settings	Delivery	71	62	-	76	53	86	83	78	53	57
52	Evaluate the effectiveness of strategies to integrate neonatal care with immunization programs to improve newborn survival and health	Delivery	71	63	-	74	57	83	73	77	49	71
53	Design and evaluate an intervention package for identification and treatment of neonatal jaundice in resource-limited settings	Delivery	71	66	-	75	62	90	85	82	34	62
54	Evaluate the effectiveness and cost of innovative dosing devices (e.g. needle free, nasal, sublingual) for antenatal corticosteroids in preventing mortality and morbidity due to preterm birth	Develop ment	71	61	-	74	50	83	75	78	51	66
55	Develop low cost, stable surfactant with novel modes of administration (e.g. nasal delivery) in order to increase use and availability of surfactant for preterm babies at risk of RDS	Discover y	71	62	-	73	49	82	82	77	51	61
56	Develop and test effectiveness and cost of innovative methods of training (such as combinations of internet-based plus face to face training, simulators, mobile applications) to increase the capacity and skills of newborn and maternal care personnel in developing countries and improve maternal, fetal and neonatal outcomes	Develop ment	70	64	-	75	55	84	80	76	49	63
57	Evaluate community-based pregnancy registration system to promote safe home delivery and immediate newborn care practices, including appropriate referral for life-threatening	Delivery	70	63	-	73	53	81	73	74	51	72

	complications											
58	Evaluate the effect of post-discharge Kangaroo Mother Care for preterm and low birth weight babies on health and survival outcomes in low and middle income countries, including long-term outcomes such as school performance	Delivery	70	62	-	74	53	86	73	75	48	67
59	Develop and evaluate easy-to-use devices for community-level neonatal resuscitation (to be delivered by community health workers/ traditional birth attendants)	Develop ment	70	60	-	74	50	76	74	74	52	72
60	Evaluate the effectiveness and cost of approaches involving parents in caring for their preterm babies in hospitals to improve quality of care, reduce time to discharge and improve newborn outcomes	Delivery	70	63	-	74	57	85	77	82	43	62
61	Evaluate approaches to improve effectiveness of integrated maternal and newborn care at facility and community levels (e.g. reducing distance between delivery room and neonatal care areas, team approach) in reducing adverse birth outcomes	Delivery	70	62	-	74	52	82	75	72	53	67
62	Evaluate effectiveness of approaches for early identification of complications of labour and real-time communication of birth attendants with skilled practitioners at regional facilities for advice, referral and transport	Develop ment	69	59	-	74	50	82	73	72	49	71
63	Evaluate acceptability and effectiveness of intermittent facility based kangaroo mother care (KMC) for preterm and low birth weight babies compared to continuous KMC or 24 hour incubator care in improving neonatal outcomes.	Develop ment	69	60	-	73	53	82	77	78	47	62
64	Evaluate effectiveness and cost of strategies for simplified antibiotics treatment of sepsis at the facility to allow an earlier discharge with further outpatient care	Develop ment	68	62	-	74	54	81	75	81	45	60

65	Develop and evaluate effectiveness and costs of easy-to-use low-cost devices to monitor maternal and newborn health (for example, blood pressure and glucose during pregnancy, warming devices, diagnostics) or facilitate provision of care in facilities and homes	Develop ment	68	62	-	76	54	80	76	76	44	65
66	Develop and evaluate a simplified algorithm for resuscitation of preterm babies in low resource settings	Develop ment	68	59	-	74	52	76	76	73	51	66
67	Evaluate the impact of programs designed to reduce anaemia in pregnant women (e.g. iron-folic acid supplementation, food fortification and growing and eating green leafy vegetables) in reducing the incidence of prematurity, intrauterine growth restriction and other adverse birth outcomes	Delivery	68	60	-	74	54	81	68	77	42	73
68	Evaluate the effectiveness and cost of a package of referral-level facility quality improvement in reducing neonatal mortality and long-term disabilities	Delivery	68	60	-	73	50	80	75	75	54	56
69	Evaluate methods to reduce nosocomial infections for all newborns in facility care	Delivery	68	60	-	72	55	83	80	81	49	46
70	Develop and evaluate improved technologies for large-scale food fortification programmes (e.g. folic acid, iodine) to prevent congenital disorders	Develop ment	68	60	-	72	51	80	79	74	39	66
71	Evaluate the impact of setting up sustainable neonatal and paediatric intensive care units in resource poor settings using simplified devices (CPAP, Inhaled surfactant, sepsis prevention and treatment) on neonatal outcomes	Develop ment	67	59	-	73	50	76	81	63	58	58
72	Evaluate the impact of family planning on reducing preterm births, small for gestational age, congenital disorders, stillbirths, and neonatal mortality	Delivery	67	57	-	72	52	75	72	67	53	70

73	Evaluate the effectiveness, cost and safety of different maternal immunization delivery strategies with existing vaccines (e.g. flu, PC, Hib, Hep B, etc.) for prevention of newborn infections	Develop ment	67	61	-	71	52	78	75	83	42	57
74	Develop and evaluate low-cost devices for measurement of serum bilirubin at the point of care in resource-limited settings to improve management	Develop ment	67	59	-	71	58	82	82	76	30	64
75	Evaluate the effectiveness of a low cost locally produced alcohol or other antiseptic-based hand rub for use at home and in health facilities on incidence of neonatal infections	Develop ment	67	59	-	71	50	82	71	73	42	65
76	Develop and validate tools for identification of preterm infants in community settings by families and community health workers that are acceptable, feasible and cost-effective	Develop ment	66	54	-	72	51	76	73	74	42	67
77	Develop and evaluate regionalized perinatal service models, including detection of high-risk pregnancies and births to promote management at appropriate levels of care through a continuum of care between home, primary, and referral level	Delivery	66	58	-	70	47	75	75	66	54	62
78	Evaluate the effectiveness of community mobilization for "emergency preparedness" in improving neonatal outcomes.	Develop ment	66	58	-	74	48	76	68	69	51	67
79	Develop and evaluate optimal feeding strategies beyond the period of exclusive breastfeeding for preterm and LBW infants	Develop ment	66	59	-	72	49	85	76	73	41	55
80	Evaluate the potential adverse effects of community-based treatment of neonatal infections, particularly changes in susceptibility of microorganisms	Delivery	66	59	-	71	53	81	68	70	46	65
81	Evaluate effectiveness and costs of maternal antibiotic prophylaxis to prevent early newborn infections	Develop ment	66	58	-	71	51	84	73	74	44	54
82	Design and evaluate accurate, yet affordable, methods to diagnose fetal distress in labour	Discover y	66	57	-	71	49	78	71	73	50	56

83	Evaluate the effectiveness of low-cost electricity-free portable fetal heart monitors to improve identification and management of perinatal asphyxia and prevent stillbirths and early neonatal deaths	Development	65	58	-	71	53	78	74	73	43	59
84	Assess effectiveness and cost of implementing a package of screening and treatment of syphilis in women of reproductive age to prevent stillbirths	Delivery	65	59	-	70	57	80	78	74	30	62
85	Evaluate approaches to improve nutritional intakes of women of reproductive age to prevent congenital disorders and improve other maternal and neonatal outcomes	Development	65	59	-	70	53	79	71	73	36	64
86	Quantify the contributions of bacterial vaginosis, chorioamnionitis and sexually transmitted diseases (e.g. syphilis) to the burden of preterm birth, and evaluate the effect of treatment of these infections on prevalence of preterm birth and other adverse birth outcomes	Development	65	57	-	68	47	77	70	71	45	61
87	Determine the effectiveness of oral neonatal vitamin A supplementation in reducing the incidence and/or severity of neonatal infections	Development	65	55	-	68	50	81	67	78	36	61
88	Evaluate the feasibility and effectiveness of a lab-on-a-chip diagnostics in reducing sepsis-specific neonatal mortality in low-income settings	Development	65	57	-	68	45	78	76	68	46	55
89	Evaluate the effectiveness and cost of a package of district hospital interventions for improved care of newborn babies	Delivery	64	53	-	69	49	73	73	71	51	54
90	Determine and assess the minimum criteria (e.g., qualifications, training, job aid etc.) for frontline community health worker to be competent in managing neonatal resuscitation	Delivery	64	56	-	69	51	77	70	69	39	66
91	Evaluate the effectiveness of filtered sunlight to treat neonatal jaundice in communities with limited or no access to conventional phototherapy	Development	64	54	-	69	56	80	73	75	28	64

92	Develop and evaluate simple tools to predict (pre-delivery) and identify (post-delivery) preterm birth and low birth weight for use by community health workers	Delivery	64	55	-	69	51	77	67	70	43	62
93	Evaluate effect and cost of strategies to deliver maternal and newborn care in humanitarian emergency contexts such as refugee and internally-displaced populations	Delivery	64	53	-	68	52	71	74	69	32	72
94	Develop and evaluate new strategies for prevention and treatment of intrauterine growth restriction	Discovery	64	51	-	68	46	77	70	67	45	59
95	Evaluate the effectiveness and cost of individual on-job mentorship/tutorship on skills acquisition and retention for frontline (first level health facility) health workers with respect to maternal and newborn care	Delivery	63	55	-	70	46	74	69	66	46	62
96	Develop and evaluate the effectiveness and cost of a package of preterm birth prevention interventions that can be implemented at scale	Delivery	63	54	-	70	46	70	65	64	57	60
97	Determine the optimal time for cord clamping in preterm infants, considering neonatal and maternal outcomes	Development	63	55	-	68	54	75	69	80	36	56
98	Develop and evaluate behaviour change strategies to improve community involvement in prevention of stillbirths and neonatal deaths	Delivery	63	53	-	70	47	71	64	65	45	69
99	Evaluate the effectiveness of different types of partographs, including pictorial partograph, in improving maternal fetal, and neonatal outcomes	Development	63	54	-	71	47	81	69	70	40	53
100	Evaluate effectiveness and cost of incentives to clinical caregivers to provide high quality care to mothers and newborns from socially disadvantaged and marginalized groups	Delivery	63	53	-	67	46	74	69	60	40	70
101	Develop novel tocolytic agents to delay or stop preterm labour, in order to reduce death/disability	Discovery	63	54	-	68	42	72	69	72	48	51

102	Evaluate possible antenatal supplementation strategies to prevent early newborn sepsis e.g., zinc, vitamin D, vitamin A	Development	62	53	-	66	47	79	64	78	37	54
103	Evaluate the effect of zinc as an adjunct to treatment of neonatal sepsis	Development	62	51	-	64	45	80	65	78	36	53
104	Evaluate the effectiveness and cost of Maternity Waiting Facilities, particularly for women at higher risk of preterm birth, for increasing coverage of perinatal care and improving maternal and neonatal outcomes	Development	62	52	-	66	44	72	71	66	42	60
105	Evaluate effectiveness and cost of a school-based program for adolescent girls to reduce known risks for future pregnancy (age at first pregnancy, anaemia reduction, awareness of risk factors, birth preparedness) in improving birth outcomes	Development	62	54	-	69	50	75	64	71	38	62
106	Evaluate the effectiveness and cost of text messages to improve management of newborn complications during the first week of life	Delivery	62	55	-	67	47	79	66	72	42	50
107	Evaluate impact of combined (integrated) educational programs in antenatal care, obstetric care, newborn resuscitation, essential newborn care and sick newborn care, compared with separate training on these topics, on maternal, fetal and newborn outcomes	Development	62	52	-	66	47	74	66	68	46	54
108	Develop and evaluate a small, portable oxygen concentrator integrated with a pulse oximeter that allows automated regulation of oxygen flow to maintain oxygen concentration within a specified therapeutic range	Development	62	53	-	66	45	74	76	67	41	51
109	Evaluate approaches to prevent pre-lacteal feeding	Delivery	61	55	-	71	46	75	74	68	34	56

110	Evaluate the effectiveness of approaches to reduce inappropriate use of broad-spectrum antibiotics to treat newborn infections	Delivery	61	53	-	67	52	79	75	68	39	46
111	Evaluate the effectiveness and cost of different health education models (e.g. school-based, community-based) in delivering the curriculum for promoting sustained health changes in young women and their families	Delivery	61	52	-	69	44	73	66	64	44	59
112	Improve the basic newborn resuscitation algorithm (decision points and timing) through a series of randomized trials	Develop ment	61	51	-	67	44	68	69	68	45	58
113	Evaluate the effectiveness and cost of training birth attendants on the appropriate use of uterotonics prior to birth in reducing intrapartum stillbirths, neonatal deaths and adverse maternal outcomes	Develop ment	61	52	-	67	45	74	71	66	40	55
114	Identify major causal pathways and risk factors for antepartum stillbirth to find potential intervention targets	Discover y	61	52	-	66	43	73	65	64	46	56
115	Investigate the pharmacokinetics of existing and emerging antibiotics so that they can be used to treat newborn infections in community and facility settings in the 1st week of life, particularly for preterm and LBW babies	Develop ment	61	49	-	63	43	71	66	82	38	46
116	Develop and evaluate effectiveness of novel point of care diagnostics for congenital syphilis that could be used in a low resource setting to improve management	Discover y	60	53	-	64	49	79	70	70	24	59
117	Evaluate the effectiveness of antenatal care clinics in promoting timely identification and care-seeking behaviour for newborns with jaundice.	Delivery	60	55	-	69	54	79	69	67	31	57
118	Identify novel antibiotic or other biological agents that could be used widely in treatment of neonatal infections	Discover y	60	51	-	65	40	72	63	68	50	49

119	Evaluate the programmes of training of community health workers in identification, management and referral of congenital disorders	Delivery	60	54	-	65	58	80	70	65	27	56
120	Evaluate approaches to overcome financial barriers to the delivery of integrated newborn services	Delivery	59	50	-	65	41	69	63	56	47	61
121	Develop and evaluate new methods for the early identification of intrauterine growth restriction (including biomarkers), and predict abnormal postnatal growth and body composition	Discovery	59	52	-	63	43	78	67	66	36	48
122	Develop and evaluate the novel vaccines for maternal immunization (e.g. GBS, Klebsiella, E. Coli, Staph, etc.) for prevention of newborn infections	Discovery	59	51	-	64	41	73	64	64	44	50
123	Evaluate the effectiveness and cost of most feasible implementation strategies for the existing point of care diagnostics for congenital syphilis in the antenatal and newborn period that could be used in a low resource setting	Development	59	51	-	63	51	73	71	66	24	60
124	Develop and evaluate strategies to prevent inappropriate use of potentially teratogenic medications during preconception, pregnancy and lactation	Development	58	46	-	##	49	70	102	59	22	39
125	Evaluate the effectiveness of strategies to encourage women to monitor fetal movements and seek care if reduced movements, in reducing stillbirths	Development	58	51	-	65	46	76	64	63	36	52
126	Evaluate if preterm birth can be delayed or averted with antioxidant and/or nutrient supplementation(s) e.g. Vitamin D, omega-3 fatty acids	Discovery	58	48	-	63	42	74	62	66	39	50
127	Evaluate the effectiveness and cost of administering an oral antibiotic to all neonates born at home who are considered to be high-risk in reducing early neonatal mortality	Development	58	49	-	65	42	66	60	59	42	63
128	Develop and evaluate the effectiveness of strategies to reduce childhood blindness due to retinopathy of prematurity, particularly through safe oxygen	Development	58	49	-	61	49	73	71	68	28	47

	management, in low resource settings											
129	Evaluate the effectiveness of oral probiotic supplementation (e.g. Bifidobacterium infantis and Lactobacillus acidophilus) in reducing incidence, duration and/or severity of neonatal infections	Develop ment	58	48	-	62	44	79	65	68	35	41
130	Examine the pathways by which pre eclampsia is associated with adverse birth outcomes including stillbirth and preterm birth and explore innovative moderating factors to delay or prevent these adverse outcomes	Discover y	57	46	-	63	41	72	62	59	43	50
131	Evaluate the effectiveness of teaching mothers appropriate ways of holding the baby for breastfeeding in improving initiation of exclusive breastfeeding and newborn survival and health in resource poor, high mortality settings	Delivery	57	49	-	63	50	81	55	64	33	52
132	Develop and evaluate the impact of strategies to reduce elective induction of labour and C-section without medical indication on prevalence of preterm birth	Delivery	57	51	-	61	49	76	72	67	33	37
133	Evaluate the impact of a program of promoting exclusive breast milk feeding to preterm babies, with provision of human donor milk if mother's own milk own breast milk is unavailable, on neonatal and child growth outcomes	Delivery	57	50	-	62	47	74	68	57	36	51
134	Evaluate the effectiveness of reduction of environmental exposures (e.g. indoor air pollution, agricultural products, occupational exposures) on adverse birth outcomes, including congenital conditions, stillbirth, low birthweight, and preterm birth	Develop ment	56	49	-	61	44	71	64	60	36	52
135	Examine interactions between preterm birth and pro-inflammatory processes of pregnancy (e.g. malaria, HIV, enteric disease) in order to inform intervention.	Discover y	56	48	-	62	41	76	61	62	37	45

136	Evaluate the effectiveness and cost of varying cadres and skills mix for health workforce teams (e.g. physicians, midwives, nurses, patient attendants etc.) to effectively deliver facility based interventions for newborn care	Delivery	56	45	-	61	41	63	63	63	44	45
137	Evaluate approaches to make therapeutic hypothermia for treatment of perinatal asphyxia (neonatal encephalopathy) safe and effective in low resource settings	Delivery	55	46	-	61	42	67	68	58	39	46
138	Evaluate the effectiveness of maternal lactoferrin supplementation, a preventative strategy for perinatal infection/inflammation, in preventing preterm birth	Develop ment	55	46	-	60	38	75	62	64	31	45
139	Evaluate the effectiveness and cost of strategies to enhance social accountability and community demand for better quality of care at birth including in the context of conditional cash transfers	Develop ment	55	46	-	61	34	60	57	58	37	63
140	Evaluate safety and efficacy of fortification of breast milk for preterm and low birth weight babies in improving growth, survival and cognitive development	Develop ment	55	46	-	62	47	72	64	63	33	43
141	Evaluate the effectiveness and cost of a kit containing basic life saving equipment given to women who are likely to deliver at home on stillbirths, neonatal mortality and morbidity.	Develop ment	55	43	-	60	44	71	57	59	35	54
142	Evaluate efficacy of various progestational compounds on spontaneous preterm birth, including evaluations of different agents and modes of delivery (e.g. vaginal micronized progesterone, gel, IM 17-alpha hydroxyprogesterone caproate), in various geographic and risk populations (e.g. short cervix, prior preterm birth, reproductive tract infections, systemic infections)	Develop ment	55	46	-	58	38	72	65	63	36	41
143	Evaluate the effectiveness of BCG vaccine at birth to newborns (and to the subset of LBW newborns) in reducing neonatal and under five mortality	Develop ment	55	45	-	62	45	70	62	67	29	48

144	Evaluate the effectiveness, cost and safety of different rubella immunization delivery strategies for prevention of congenital rubella infections	Develop ment	55	48	-	63	52	71	67	69	20	47
145	Evaluate the effect of timing (preconception period vs. first or second trimester of pregnancy) for micronutrient supplementation to prevent congenital disorders	Develop ment	55	47	-	61	47	70	64	63	29	49
146	Evaluate whether preconception nutritional supplementation of mothers leads to long-term improvements in health and development in the children	Develop ment	55	46	-	62	45	68	58	62	33	53
147	Develop new formulations of iron (or new medicines which when given with iron) that reduce side effects and increase the bioavailability when given as supplement to preterm babies	Develop ment	55	48	-	62	42	70	59	68	30	46
148	Evaluate efficacy of treating neonatal/congenital malaria with injectable artesunate given orally (with and without a first phase of intravenous treatment) to prevent mortality and long term disability	Delivery	55	47	-	59	38	71	63	62	27	50
149	Evaluate the acceptability, benefits and risks of introducing portable ultrasound scanning into the community	Develop ment	55	46	-	59	47	72	59	59	32	51
150	Evaluate the effectiveness and cost of implementing newborn screening and management for common inherited metabolic disorders in preventing mortality and long term disability	Delivery	54	48	-	59	58	80	70	60	21	40
151	Evaluate and compare the effectiveness and cost of pre-service vs. in-service training on newborn care skills acquisition, use and retention	Delivery	54	42	-	59	42	67	66	65	32	40
152	Evaluate the effect of improved management of neonatal sepsis on long term developmental outcomes	Develop ment	54	46	-	63	43	63	62	63	34	49
153	Develop strategies for early identification of newborns at risk of neglect and evaluate interventions to prevent adverse outcomes in these	Delivery	54	45	-	58	45	70	57	54	30	58

	newborns											
154	Evaluate which components of neonatal resuscitation (stimulation, suction and ventilation) are most effective in reducing intrapartum-related deaths when performed by first level care providers	Develop ment	53	41	-	59	41	59	57	63	37	51
155	Determine the factors that shape the amount of public financing that the governments of countries with high neonatal mortality devote to newborn survival	Delivery	53	45	-	59	36	66	51	52	42	55
156	Evaluate methods to improve diagnosis and care of babies born with common congenital disorders in developing countries	Delivery	53	46	-	58	56	76	62	61	24	44
157	Develop and evaluate novel approaches to prevent mother to the baby infection transmission, e.g., maternal showering, vaginal douche, antiseptic wash of baby	Develop ment	53	46	-	59	43	72	62	56	32	45
158	Identify the mechanisms behind the socioeconomic gradient in the risk of preterm birth, intrauterine growth restriction and other adverse birth outcomes in order to inform strategies to reduce inequalities	Discover y	53	44	-	57	38	61	53	53	36	64
159	Develop new adjuvant therapies to therapeutic hypothermia for management of asphyxiated newborns	Discover y	53	44	-	59	40	71	66	54	32	40
160	Evaluate the effect of interventions to reduce prenatal exposure to environmental tobacco smoke (second-hand smoke) on the risk of preterm birth, intra uterine growth restriction, stillbirth and neonatal death	Develop ment	52	44	-	58	44	65	58	58	32	49
161	Identify the facilitating and inhibiting factors that govern the financial support of international donor agencies to newborn health in order to increase the amount and sustainability of funding	Delivery	52	42	-	60	34	62	53	53	44	50

162	Evaluate the effectiveness and cost of lifestyle modification interventions in the mother (e.g. diet, smoking and exercise) in reducing stillbirths and improving neonatal outcomes	Develop ment	52	42	-	58	39	64	59	54	36	49
163	Evaluate strategies for control of alcohol use in women of reproductive age to prevent fetal alcohol spectrum disorders	Delivery	52	44	-	57	52	72	65	62	19	43
164	Evaluate the effectiveness of community-based use of pulse oximetry to reduce mortality and morbidity of sick and/or small newborns.	Develop ment	52	44	-	58	41	66	58	55	28	53
165	Identify and validate early novel biochemical markers of perinatal asphyxia and hypoxic ischemic encephalopathy in order to inform earlier targeted interventions	Discover y	52	43	-	59	39	69	61	54	35	41
166	Evaluate the efficacy of a package of interventions for intrauterine fetal resuscitation in low and middle income countries in reducing intrapartum stillbirths and early neonatal deaths	Develop ment	51	40	-	57	37	63	56	57	38	42
167	Develop and evaluate the feasibility of using novel wireless pulse oximeter probes to measure fetal oxygenation	Develop ment	51	42	-	56	46	67	60	59	27	44
168	Evaluate the effectiveness and cost of using ultrasound devices with automatic estimation of gestational age in ANC on perinatal outcomes	Delivery	51	41	-	55	49	78	57	52	28	38
169	Describe political and bureaucratic (rather than scientific or health systems) factors that facilitate or obstruct the scale-up of newborn survival interventions in countries with high neonatal mortality	Delivery	51	41	-	55	36	59	51	45	41	57
170	Develop simplified assessment tools for mother's mental health state, delineate the effects on newborn health and test interventions to improve neonatal and child development outcomes	Delivery	50	42	-	55	44	64	56	56	24	53

171	Evaluate the impact of home pregnancy testing on gestational age at enrolment for ANC and seeking care from skilled health providers	Develop ment	50	43	-	58	46	66	59	53	25	47
172	Investigate appropriate indications for use of vaginal pessary for the prevention of spontaneous preterm birth and evaluate its efficacy	Develop ment	50	42	-	53	34	65	55	53	34	42
173	Develop and evaluate low cost tools to identify manageable congenital disorders at community and primary health care level	Develop ment	50	42	-	57	50	64	63	58	19	44
174	Evaluate efficacy of erythropoietin added to therapeutic hypothermia, to enhance neuroprotection in late preterm and full term infants with neonatal encephalopathy, in improving neonatal outcomes	Discover y	49	40	-	55	36	65	62	53	28	37
175	Evaluate the effectiveness and cost of licensure/relicensure schemes for providers and accreditation/reaccreditation schemes for healthcare facilities to improve quality of services provided through both public and private systems	Delivery	49	39	-	54	37	65	53	52	35	40
176	Develop neuroprotective and neuroregenerative therapies to reduce developmental brain injury in preterm infants	Discover y	49	40	-	55	36	63	53	49	38	41
177	Evaluate the effect of reduced physical workload during pregnancy on the occurrence of adverse birth outcomes	Develop ment	48	43	-	56	44	63	55	49	26	48
178	Identify novel adjuvants / immunomodulators that could be used widely in treatment of neonatal infections	Discover y	48	37	-	53	36	65	54	51	33	38
179	For pathogens that cross the placental barrier, determine if there is a common mechanism(s) used by several pathogens that could become a target for development of future interventions to prevent neonatal infections	Discover y	48	37	-	53	37	57	57	52	34	40

180	Evaluate the effectiveness of administering an oral antibiotic to all neonates born at home in reducing early neonatal mortality	Develop ment	47	38	-	54	41	58	48	44	35	53
181	Validate the use of most promising candidate biomarkers that can guide the initiation and/or duration of antibiotic therapy for neonatal sepsis (e.g. CD64, interleukins, etc.) to improve management	Develop ment	47	39	-	51	41	68	57	47	30	32
182	Evaluate the effectiveness of allopurinol, given to pregnant women with signs of fetal hypoxia, to improve newborn morbidity and later morbidity	Discover y	47	36	-	55	36	62	53	56	28	35
183	Develop and evaluate the effect of community and facility level strategies that raise the profile of the neonate as a human being with a right to live to change the rooted social norm that accepts neonatal deaths	Develop ment	46	36	-	52	40	53	46	51	31	51
184	Develop novel molecular tests to assess placental function, including that in post-date pregnancies in odrer to reduce stillbirths, early neonatal deaths and later disability	Discover y	46	36	-	49	41	64	56	45	30	35
185	Evaluate whether pre-conceptional nutritional supplementation of mothers leads to epigenetic changes in offspring DNA (measured in placenta, umbilical cord and blood).	Discover y	46	37	-	52	40	67	53	47	26	37
186	Develop and utilize non-invasive assessments to image and characterize the normal development of the placenta across gestation and changes in adverse pregnancy states	Discover y	45	36	-	50	40	65	49	45	29	38
187	Develop and evaluate novel micronutrient strategies to reduce cerebral damage related to perinatal asphyxia in order to reduce death and disability	Discover y	45	35	-	51	38	58	42	54	29	39
188	Identify and validate genomic, proteomic, and epigenetic biomarkers and biochemical markers in maternal blood, amniotic fluid, saliva, vaginal secretions etc. for prediction of preterm birth,	Discover y	44	36	-	49	36	56	52	48	31	35

	intrauterine growth restriction, and other adverse pregnancy outcomes											
189	Evaluate the effectiveness of strategies to promote safe sleep environments to reduce the incidence of SIDS, accidental suffocation, and other sudden unexpected deaths in infancy	Delivery	44	36	-	49	48	62	55	53	18	31
190	Understand the changes in the normal microbiome in pregnancy and contrast to that in pregnancies complicated by adverse outcomes (preterm birth, stillbirth, preeclampsia)	Discover y	44	35	-	49	39	64	49	48	25	32
191	Discover unique inflammatory signature of most common pathogens associated with neonatal infections to develop and evaluate novel tailored interventions to prevent pathogen specific inflammatory response	Discover y	43	34	-	49	35	54	52	47	28	34
192	Evaluate the effectiveness of a novel, fish oil based lipid emulsion, in reducing liver damage associated with parenteral nutrition in neonates with congenital gastrointestinal disorders	Discover y	43	34	-	48	41	64	52	51	18	29
193	Evaluate effectiveness and cost of cervical cerclage for prevention of preterm birth in low- and middle-income countries, including development and assessment of ultrasound technologies that are affordable and appropriate for use in low-resource settings	Develop ment	42	33	-	47	37	55	51	44	28	34
194	Discover unique inflammatory signature of most common pathogens associated with neonatal infections in order to tailor the existing interventions to prevent pathogen specific inflammatory response.	Discover y	41	31	-	48	37	54	47	46	25	35
195	Evaluate geographic variation in the microbiome of pregnant women and validate potential biomarkers that may predict preterm birth and specific phenotypes	Discover y	41	33	-	47	39	58	44	43	25	36

196	Evaluate the effectiveness of use of room air compared to 100% oxygen during resuscitation of term newborns born at home in improving short-term newborn outcomes	Develop ment	40	32	-	47	45	51	46	43	21	40
197	Develop and evaluate the efficacy of growth stimulating factors or other novel approaches to prevent broncho-pulmonary-disease for preterm babies	Develop ment	40	32	-	46	40	54	49	47	21	27
198	Evaluate public educational strategies for prevention of inherited inborn errors of metabolism and single-gene disorders	Develop ment	38	33	-	47	44	56	42	45	15	34
199	Develop and evaluate interventions to reduce oxidative stress to prevent placental senescence and evaluate their effects on stillbirth and other adverse birth outcomes	Discover y	38	28	-	44	38	54	44	40	24	28
200	Find biomarkers to identify newborns who do not benefit from therapeutic hypothermia for perinatal asphyxia	Discover y	34	26	-	42	42	51	39	37	19	23
201	Evaluate the effectiveness of promoting 5 instead of 3 years birth interval on preventing stillbirths and improving newborn survival and health	Develop ment	34	25	-	41	47	45	36	33	22	33
202	Develop and evaluate interventions to reduce psychosocial stress to prevent placental senescence and evaluate their effects on stillbirth and other adverse birth outcomes	Develop ment	34	26	-	40	42	44	37	41	18	30
203	Assess the validity of maternal blood nucleic acid testing for detection of fetal genetic anomalies to improve management	Develop ment	34	28	-	40	47	53	42	35	14	23

The questions originally ranked 5th and 12th were omitted from this table because it was a variant of question that already received a higher overall score.

S8. Composition of the group of technical experts

Name	Affiliation
Peter Aaby	Prof, DMSc, Bandim Health Project, Indepth Network, Guinea-Bissau
Ishag Adam	Prof, MD, PhD, Faculty of Medicine, University of Khartoum, Sudan
Ramesh Kant Adhikari	Prof, MD, Kathmandu Medical College, Nepal
Namasivayam Ambalavanan	MD, Department of Pediatrics, University of Alabama at Birmingham, Birmingham, AL, USA
Shams El Arifeen	Dr, DrPH, Centre for Child and Adolescent Health, International Centre for Diarrhoeal Disease Research, Bangladesh
Dhana Raj Aryal	Prof, Department of Neonatology Paropakar Maternity and women's Hospital, Nepal
Sk Asiruddin	MD, MSc, TRAction Bangladesh Project, University Research Co., LLC
Abdullah Baqui	Prof, PhD Johns Hopkins Bloomberg School of Public Health, USA.
Aluisio JD Barros	Centro de Pesquisas Epidemiológicas, Universidade Federal de Pelotas, Brazil
Christine S. Benn	Prof, MD, PhD, DMSc, Research Center for Vitamins and Vaccines, Statens Serum Institut, and University of Southern Denmark/Odense University Hospital, Denmark
Vineet Bhandari	MD, Program in Perinatal Research, Yale University School of Medicine, USA
Shinjini Bhatnagar	PhD, Pediatric Biology Centre, Translational Health Science and Technology Institute, India
Sohinee Bhattacharya	Dr MBBS PhD University of Aberdeen, UK
Zulfiqar A Bhutta	MB,BS, PhD, Center of Excellence in Women and Child Health, the Aga Khan University, Karachi, Pakistan
Robert E Black	Prof, MD, Institute of International Programs, Johns Hopkins Bloomberg School of Public Health, USA.
Hannah Blencowe	MRCPCH, The London School of Hygiene & Tropical Medicine, UK
Carl Bose	Prof, MD, The University of North Carolina at Chapel Hill School of Medicine, USA
Justin Brown	Thrasher Research Fund, USA
Christoph Bührer	Prof, MD, Department of Neonatology, Charité University Medical Center, Germany
Wally Carlo	University of Alabama at Birmingham, USA
Jose Guilherme Cecatti	Prof, MD PhD, Department of Obstetrics and Gynaecology, School of Medical Sciences, University of Campinas, Brazil
Po-Yin Cheung	Prof, PhD, FRCP (Can&Edin), Departments of Pediatrics, Pharmacology & Surgery, University of Alberta, Canada
Robert Clark	MD MPH, LDS Charities, USA
Tim Colbourn	PhD, University College London Institute for Global Health, UK

Agustin Conde-Agudelo	MD, MPH, PhD, Perinatology Research Branch, Eunice Kennedy Shriver National Institute of Child Health and Human Development/National Institutes of Health/Department of Health and Human Services, Bethesda, Maryland and Detroit, Michigan, USA
Erica Corbett	MSc, Independent consultant maternal health research, Rwanda
Andrew E Czeizel	Prof, MD, DoctSci, Foundation for the Community Control of Hereditary Diseases, Hungary.
Abhik Das	PhD, Biostatistics and Epidemiology, RTI International, USA
Louise Tina Day	Dr, MRCPCH MRCOG, LAMB Integrated Rural Health & Development, Bangladesh
Carolyn Deal	Division of Microbiology and Infectious Diseases, National Institute of Allergy and Infectious Diseases National Institute of Health, USA
Ashok Deorari	All India Institute of Medical Sciences, India
Uğur Dilmen	Prof, Pediatrics and Neonatology, Yıldırım Beyazıt University Medical Faculty, Turkey
Mike English	MD, Nuffield Department of Medicine & Department of Paediatrics, University of Oxford, UK and KEMRi-Wellcome Trust Research Programme, Nairobi, Kenya
Cyril Engmann	MD, Newborn Health, Family Health Division, The Bill & Melinda Gates Foundation and the University of North Carolina Schools of Medicine and Public Health, USA
Fabian Esamai	Prof, PhD, Moi University, School of Medicine, Kenya
Caroline Fall	Prof MD, International Paediatric Epidemiology; Affiliations: Medical Research Council Lifecourse Epidemiology Unit, University of Southampton, UK
Donna M Ferriero	Prof, MD, Department of Pediatrics, UCSF Benioff Children's Hospital, USA
Peter Gisore	School of Medicine, Child Health and Pediatrics, Moi University, Kenya
Tabish Hazir	Prof, MD Children's Hospital, Pakistan Institute of medical sciences, Pakistan
Rosemary D. Higgins	MD, Eunice Kennedy Shriver NICHD Neonatal Research Network, Pregnancy and Perinatology, Branch, National Institute of Health, USA
Caroline SE Homer	Prof, Centre for Midwifery, Child and Family Health, University of Technology, Sydney, Australia
Dewan Md Emdadul Hoque	Dr, MPH, Centre for Child and Adolescent Health, International Centre for Diarrhoeal Disease Research, Bangladesh
Lorentz Irgens	University of Bergen and Norwegian Institute of Public Health, Norway
Mohammad Tajul Islam	MPH, Japan International Cooperation Agency (JICA), Bangladesh
Joseph Johnson	DrPH Save the Children, USA
Martias Joshua	MB,BS MPH, Zomba Central Hospital, Ministry of Health, Malawi

William Keenan	MD, Saint Louis University, USA
Soofia Khatoon	Paediatrics and Head of Department Shaheed Suhrawardy Medical College, Bangladesh.
Helle Kieler	Centre for Pharmacoepidemiology, Karolinska Institute, Sweden
Michael S. Kramer	Prof, MD, Departments of Pediatrics and of Epidemiology, Biostatistics and Occupational Health, McGill University Faculty of Medicine, Montreal, Quebec, Canada
Eve M Lackritz	MD, Global Alliance to Prevent Prematurity and Stillbirth (GAPPS), USA
Tina Lavender	Prof, University of Manchester School of Nursing Midwifery & Social Work, University of Manchester, UK
Laurensia Lawintono	MSc Indonesian Midwives Association, Indonesia
Richard Luhanga	Save the Children, Malawi
David Marsh	MD, MPH, Save the Children, USA
Douglas McMillan	Prof, MD, Department of Paediatrics, Dalhousie University, Canada
Patrick J McNamara	MD, Departments of Paediatrics & Physiology, University of Toronto; Physiology & Experimental Medicine program, Hospital for Sick Children, Toronto, Canada
Ben Willem J Mol	Prof,. MD PhD. Department of Obstetrics and Gynaecology, Academic Medical Centre Amsterdam, the Netherlands
Elizabeth Molyneux	Prof, Paediatrics and Child Health, College of Medicine Malawi
G. K. Mukasa	International Baby Food Action Network, Uganda
Miriam Mutabazi 0	MD, STRIDES for Family Health, Management Sciences for Health, Uganda
Luis Carlos Nacul	Dr MD PhD, Faculty of Infectious and Tropical Diseases, London School of Hygiene and Tropical Medicine, UK
Margaret Nakakeeto	Kampala Children's Hospital Limited and Childhealth Advocacy International, Uganda
Indira Narayanan	MD, United States Agency for International Development /Maternal and Child Health Integrated Program, USA.
Bolajoko Olusanya	FRCPCH, Centre for Healthy Start Initiative, Nigeria
David Osrin	MRCP PhD, Wellcome Trust Senior Research Fellow in Clinical Science, Institute for Global Health, University College London, UK
Vinod Paul	Prof, PhD, All India Institute of Medical Sciences, India
Christian Poets	University of Tubingen, Germany
Uma M. Reddy0	Eunice Kennedy Shriver National Institute of Child Health and Human Development National Institutes of Health, USA
Mathuram Santosham	Center for American Indian Health, Johns Hopkins Bloomberg School of Public Health, USA.
Rubayet Sayed.	Save the Children, Bangladesh
Natalia E. Schlabritz-	MD, PhD, Department of Obstetrics and Gynecology, University of Tennessee Health Science Center, USA

Loutsevitch	
Nalini Singhal	Prof, FRCPC, University of Calgary, Canada.
Mary Alice Smith	Environmental Health Science Department, University of Georgia, USA
Peter G. Smith	Prof, DSc, MRC Tropical Epidemiology Group, London School of Hygiene & Tropical Medicine, London , UK.
Sajid Soofi	MD, Department of Pediatrics & Child Health, Women & Child Health Division, Aga Khan University, Pakistan
Catherine Y. Spong	Eunice Kennedy Shriver National Institute of Child Health and Human Development, National Institutes of Health, USA
Shahin Sultana	National Institute of Population Research and Training (NIPORT), Ministry of Health and Family Welfare, Bangladesh
Antoinette K. Tshefu	Prof, PhD, Kinshasa School of Public Health, School of Medicine, University of Kinshasa, Democratic Republic of Congo
Bel F. Van	Prof, Department of Neonatology, University of Utrecht, The Netherlands
Lauren Vestewig Gray	MA, Institute for Global Health Technologies Rice University, USA
Peter Waiswa	MD, DrPH, Division of Global Health, Karolinska Institutet, Sweden
Wei Wang	Prof, MD, PhD, FFPH, School of Medical Sciences, Edith Cowan University, Australia and School of Public Health, Capital Medical University, China
Sarah L A Williams	Save the Children UK
Linda L Wright	MD, Eunice Kennedy Shriver National Institute of Child Health and Human Development National Institutes of Health, USA
Anita Zaidi	Prof, Aga Khan University, Pakistan
Yanfeng Zhang	MSc, Department of Integrated Early Childhood Development, Capital Institute of Paediatrics, China.
Nanbert Zhong	Prof, MD, Developmental Genetics Laboratory, New York State Institute for Basic Research in Developmental Disabilities, USA
Isabel Zuniga	MD MPH, Médecins sans Frontières, Belgium

Supplementary table S9: Pairs (or triplets) of questions from the initial list that had similarities or overlap in their content, but received different overall scores. They were used of an in-depth analysis of the reasons for divergence in overall research priority scores received by the similar research ideas. The yellow cells highlight the differences in intermediate scores greater than 5.0 points, which were discussed in **Box 1**.

RANK	RPS	RESEARCH QUESTIONS	ANSWERABLE?	EFFICACY?	DELIVERABLILITY?	IMPACT?	EQUITY?
**13**	79	Evaluate approaches to improve community based postnatal care (e.g. home visits) within the context of existing health system	85.6	81.5	84.5	59.2	82.3
**37**	73	Evaluate the effectiveness of Traditional Birth Attendants carrying out early postnatal home visits for newborns who were born at home in improving newborn health outcomes	84.8	77.6	74.4	48.0	80.2
**41**	73	Develop and evaluate the impact of models for strengthening capacity of health professionals in caring for neonates in peripheral hospitals and lower level health facilities on improving neonatal outcomes	78.7	79.0	77.2	56.6	71.8
**56**	70	Develop and test effectiveness and cost of innovative methods of training (such as combinations of internet-based plus face to face training, simulators, mobile applications) to increase the capacity and skills of newborn and maternal care personnel in developing countries and improve maternal, fetal and neonatal outcomes	83.9	80.0	75.6	49.1	63.1
**8**	80	Facility based initiation of Kangaroo Mother Care or continuous skin-to-skin contact	90.5	81.9	84.4	61.6	81.0
**58**	70	Evaluate the effect of post-discharge Kangaroo Mother Care for preterm and low birth weight babies on health and survival outcomes in low and middle income countries, including long-term outcomes such as school performance	86.3	72.6	75.3	48.2	67.3
**11**	80	Evaluate the effectiveness and cost of "extra care" for preterm/LBW babies at community level by trained community health workers including identification, additional home visits, supportive referral etc. in reducing neonatal morbidity and mortality in settings with poor accessibility to facility care	87.2	86.9	81.0	62.2	81.0
**21**	78	Develop and evaluate the effectiveness and cost of a package of community follow-up care for preterm and low birth weight newborns after discharge from a health facility	88.0	86.6	81.9	57.6	73.5
**68**	68	Evaluate the effectiveness and cost of a package of referral-level facility quality improvement in reducing neonatal mortality and long-term disabilities	79.5	75.3	75.3	53.7	55.8
**89**	64	Evaluate the effectiveness and cost of a package of district hospital interventions for improved care of newborn babies	72.9	72.6	71.3	50.1	53.9

**76**	66	Develop and validate tools for identification of preterm infants in community settings by families and community health workers that are acceptable, feasible and cost-effective	75.6	73.2	74.1	42.0	67.1
**92**	64	Develop and evaluate simple tools to predict (pre-delivery) and identify (post-delivery) preterm birth and low birth weight for use by community health workers	77.3	67.0	70.5	42.5	62.2
**1**	90	Simplified neonatal resuscitation program delivered by trained health worker in reducing neonatal deaths due to perinatal asphyxia	96.1	91.0	93.8	77.0	92.1
(omitted)	82	Evaluate the effectiveness and cost of making a bag and mask accessible to trained individuals or teams attending births in reducing neonatal mortality	85.9	80.6	86.1	58.8	96.2
**2**	88	Approaches to scale up worker skills in preventing and managing asphyxia	96.0	91.5	89.3	75.1	85.7
(omitted)	84	Evaluate the effectiveness and cost of training nurses/midwives in intrapartum fetal monitoring and neonatal resuscitation in reducing stillbirths and deaths/disability due to perinatal asphyxia	93.6	89.0	91.4	65.1	80.0
**5**	83	Training of CHWs in basic newborn resuscitation in reducing morbidity and mortality due to perinatal asphyxia	94.3	84.1	84.1	63.8	87.8
**39**	73	Evaluate the effectiveness and cost of various approaches (direct observation, mannequins, scenarios with hyper-local videos, scenarios alone, among others) for sustaining newborn resuscitation skills of CHWs	86.8	79.4	78.6	50.2	69.1
**93**	64	Evaluate effect and cost of strategies to deliver maternal and newborn care in humanitarian emergency contexts such as refugee and internally-displaced populations	77.1	69.6	68.5	39.3	65.6
**10**	79	Quality of care during labour and birth in reducing intrapartum stillbirths and reduce neonatal mortality and disability	82.6	84.3	81.8	72.1	74.7
**50**	71	Develop and evaluate strategies to increase correct use of partograph, linked to appropriate action	81.6	80.7	80.4	50.9	63.2
**33**	74	Evaluate effectiveness and cost of perinatal audits to improve quality of care in health facilities and improve fetal and neonatal outcomes	89.9	83.1	80.2	52.9	62.8
**35**	74	Develop and evaluate simple, cost effective strategies for intrapartum monitoring to enhance timely referral to improve fetal and neonatal outcomes	88.4	82.6	75.6	55.9	65.7

**82**	68	Design and evaluate accurate, yet affordable, methods to diagnose fetal distress in labour	78.3	70.7	72.6	50.2	67.9
**83**	65	Evaluate the effectiveness of low-cost electricity-free portable fetal heart monitors to improve identification and management of perinatal asphyxia and prevent stillbirths and early neonatal deaths	78.2	73.8	72.9	43.3	58.9
**14**	79	Evaluate the effectiveness and cost of parental education on practices to prevent newborn infections at home, including recognition of neonatal infection and care seeking for it	84.8	81.3	85.5	60.5	81.0
**29**	76	Compare the effectiveness and cost of improving families' illness recognition and care-seeking for newborn illness versus active home visits in increasing the coverage of treatment for sick newborns	86.9	76.5	81.5	53.9	78.7
**18**	78	Evaluate effectiveness and cost of provision of clean delivery kits and capacity building of traditional birth attendants in reducing incidence of neonatal infections	84.7	79.7	83.9	55.6	85.5
**43**	72	Evaluate the effectiveness and cost of approaches to increase coverage of clean delivery practices in homes	77.8	72.7	77.8	56.7	76.2
**36**	74	Evaluate the effectiveness and cost of training community health workers to recognize and treat neonatal sepsis at home with oral antibiotics (such as third generation cephalosporins) when referral is not possible, on neonatal mortality.	84.7	77.1	75.0	53.7	77.2
**38**	73	Evaluate effectiveness of oral amoxicillin at home, with supervised home visits, for treatment of neonatal pneumonia	81.8	76.2	79.5	51.5	75.9
**70**	68	Develop and evaluate improved technologies for large-scale food fortification programmes (e.g. folic acid, iodine) to prevent congenital disorders	80.1	79.3	74.1	38.6	65.8
**85**	65	Evaluate approaches to improve nutritional intakes of women of reproductive age to prevent congenital disorders and improve other maternal and neonatal outcomes	78.7	71.4	73.5	35.7	64.5

References

1. Rudan I, Gibson JL, Ameratunga S, El Arifeen S, Bhutta ZA, Black M, et al. Setting priorities in global child health research investments: Guidelines for implementation of the CHNRI method. Croat Med J 2008;49:720–733.

2. Rudan I, Chopra M, Kapiriri L, Gibson J, Lansang MA, Carneiro I, Ameratunga S, et al. Setting Priorities in Global Child Health Research Investments: Universal Challenges and Conceptual Framework. Croat Med J 2008; 49:307–317.

3. Fontaine O, Kosek M, Bhatnagar S, Boschi-Pinto C, Chan KY, Duggan C, Martinez H, et al. Setting research priorities to reduce global mortality from childhood diarrhoea by 2015. PLoS medicine 2009;6(3).

4. Rudan I, El Arifeen S, Bhutta ZA, Black RE, Brooks A, Chan KY, Chopra M, et al. Setting research priorities to reduce global mortality from childhood pneumonia by 2015. PLoS medicine 2011, 8(9).

5. Lawn JE, Bahl R, Bergstrom S, Bhutta ZA, Darmstadt GL, Ellis M, English M, et al. Setting research priorities to reduce almost one million deaths from birth asphyxia by 2015. PLoS medicine 2011;8(1).

6. Bahl R, Martines J, Ali N, Bhan MK, Carlo W, Chan KY, Darmstadt GL, et al. Research priorities to reduce global mortality from newborn infections by 2015. Pediatr Infect Dis J 2009;28:S43-8.

7. Bahl R, Martines J, Bhandari N, Biloglav Z, Edmond K, Iyengar S, Kramer M, et al. Setting research priorities to reduce global mortality from preterm birth and low birth weight by 2015. J Glob Health 2012;2:10403.

8. Tomlinson M, Swartz L, Officer A, Chan KY, Rudan I, Saxena S. Research priorities for health of people with disabilities: an expert opinion exercise. Lancet 2009 Nov 28;374: 1857–62.

9. Tomlinson M, Rudan I, Saxena S, Swartz L, Tsai AC, Patel V. Setting priorities for global mental halth research. Bulletin of the World Health Organization 2009; 87:438–446.

Raw data

CRITERION 1: ANSWERABILITY Based on:(i) the clarity of the question and its endpoints; (ii) the level of required research capacity (iii) the size of the gap from current level of knowledge; (iv) ethical considerations: Would you say that the question can be answered in ethical way?

Scores (n=91), columns 1–46

Research Questions (n=205)	1	2	3	4	5	6	7	8	9	10	11	12	13	14	15	16	17	18	19	20	21	22	23	24	25	26	27	28	29	30	31	32	33	34	35	36	37	38	39	40	41	42	43	44	45	46
1	1	1	1	1	1	1	1	1	1	1	1	1	0.5	0	1		0.5		1	1	0.5	1	0	1	0.5	1	0.5	0.5	0	0	1	1	1	1	1	0.5	1	1	1	1	0	1	0	1	1	1
2	1	1	1	1	0	1	1		1	1	1	1	1	1	1	0	0.5		1	1	1	1	0.5	1	1	1	1	1	1	1	1	1	1	1	0.5	0.5	0.5	1	0	1	0.5	1	1	1	1	0.5
3	1	0	1	1	1	1	1	1	1	0.5	0	1	0.5	1	0	0	1		1	1		0	0.5	0	0.5	0.5	1	1	1	1	1	1		1	0	1	0.5	1	0.5	1	1	0		0.5	1	1
4	1	0	1	1	1	1	1	1	1	1	1	1	1		1	0	1		1	0	0	1	1	1	1	1	0.5	0.5	0	0	0.5	1	1	1	1	0	1	1	1	1	1	1	1	1	1	1
5	1	0	1	1	1	1	1		1	1	1	1	1	1	1	1	1	1	1	0	0.5	1	1		1	1	1	0.5	0	0	1	1	1	1	1	0.5	1	0	1	1	0.5	1		1	1	1
6	1	0	1	1	0	1	1		1	1	1	1	0	1	1	1	1	1	1	0	0.5	1	1	1	1	1	1	0.5	0	0	1	1	1	1	1	1	0	0	1	1	0.5	0	1	1	1	1
7	1	1	1	0	1	1	1	1	1	1	1	1	1	1	1	1	1	0	1	0	1	1	0		0	1	0.5	1	1	1	0.5	1	1	1	0		1	0	1	1	0	1	1	1	0.5	1
8	1	0	1	0.5	1	1	1	1	1	1	1	1	0		1	1	1	1	1	1	1	1	1	1	1	1	1	1	0	0	1	1	1	1	1	1	1	0	0.5	1	1	1	0.5	1	1	1
9	1	1	0.5	0	1	1	1	1	1	1	1	1	0.5	1	1		1	0	1	1	0.5	1	1		0	1	0.5	1	0	0	1	1	1	1	0	1	0	0	0.5	1	1	0	1	0	1	1
10	1	0	1	1	1	1	1	1	1	1	1	1	1	1	1		1	1	1	1	1	1	0.5	1	1	1	1	1	0	1	0.5	1	1	1	1	1	1	0	1	1	1	1	1	1	1	1
11	1	0	1	1	1	1	1	1	1	1	1	1	1	1	1		1		1	1	1	1	1		1	1	0.5	1	0	0	1	1		1	1	1	1	0	1	0.5	1	1	1	1	1	1
12	1	1	1	1	1	1	1	1	1	1	1	1	1	1	1		1		1	1	1	1	1		1	1	1	1	0	0	1	1		1	0	1	1	0	1	1	1	0	0.5	0	1	0.5
13	1	1	1	1	1	1	1		1	1	1	1	1	0	1	1	1	1	1	1	1	1			1	1	1	1	1	1	1	1	1	1	0.5	1	1	0	1	1	0	1	0	1	1	1
14	1	0	1	1	0	1	1		1	1	1	1	0	1	1	1	1	1	1	1	1	1	1	1	1	1	0.5	1	0	0	1	1	1	1	0	1	0	0	0.5	1	0.5	1		0	1	1
15	1	1	1	1	1	1	1	1	1	1	1	1	1	1	1	1	1	1	1	1	1	1	1	1	1	1	1	0.5	1	1	1	1	1	1	1	1	1	0	1	0.5	1	0	0.5	0.5	1	
16	1	0	1		1	1	1		1		1	1	1		1	1	0.5	0	1	0		1	0		1		1	0.5	1	1		1		1	0	0.5	1	0		1	0.5	0		1	0.5	
17	1	0	1	0.5	1	1	1	1	1	0.5	1	1	1		1	1	1	1	1	1	1	1	1	0	0.5	1	1	0	0	0		1	0.5	1	1	1	0.5	0	0	1	1	0	0.5	1	1	1
18	1	1	1	1	0	1	1	1	1	1	1	1	1	1	1	1	1		1	1	1	1		1	1	1	0.5	1	1	1	1	1	1	1	1	1	1	0	1	1	1	0	0.5	1	1	1
19	1	0	0.5	0.5	1	1	1	1	1	1	1	1	1		1	1	1	1	1	1	1	1	1		1	1	1	1	0	0	1	1	1	1	0	1	0	0	0.5	1	1	1	1	0	1	1
20	1	0	1	1	1	1	1	1	1	0.5	1	1	0.5		1	1	0.5	1	0.5	1	1	1	0		1	1	0.5	0	0	0	1	1	1	1	0	0.5	0	0	0.5	0.5	0.5	1	1	0.5	1	1
21	1	0	1	0	1	1	1	1	1	1	0	1	1	1	1	1	1		1	1	0	1	1	1	1	1	1	1	1	1	1	1	0.5	1	1	1	1	0	1	1	1	0	1	1	1	
22	1	0	1	0	1	1	1	1	1	1	0	1	1	1	1	1	0.5	1	1	1	1	1	1		1	1	0	1	1	1	1	1	1	1	1	1	1	0	1	1	1	1		1	1	
23	1	1	1	0	1	1	1	1	1	1	1	1	0.5	1	1	1	1	1	1	1	1	1	1	1	1	1	1	1	1	1	1	1	1	1	1		1	0	1	1	0.5	1	1	1	1	1
24	1	1	1	1	1	1	1	1	1	1	1	1	1		1	0	0	1	1	1	0.5	1	1	1	1	1	1	1	1	1	1	1	1	1	1	1	1	0	1	0.5	0	0		1	1	1
25	1	0	1	0	0	1	1	1	1	0	1	1	0.5	1	1	1	1	1	1	1	1	1	1	1	0.5	1	1	1	0	0	1	1	1	1	0.5		0	0	0.5	1	0.5	1	0.5	1	1	1
26	1	0	1	0	0	1	1		1	1	1	1	1		1	1	1			0	1	1			1	1	0.5	0.5	0	0	1	1		1	0	0.5		0	1	0.5		1	1	0.5	1	1
27	0.5	0	1	1	0	1	1	1	1	0.5	0.5	1	1		1		1		0.5	1	1	1			1		0	0.5	0	1		1		1	0	0.5	0	0	0.5	1	0	0			0.5	0.5
28	1	0.5	1	1	1	1	1	1	1	1	1	1	1		1	1	1	1	1	1	1	1			1	1	1	1	1	1	1	1	1	1	0.5	1	1	0	1	0.5	1	1	1	1	1	1
29	1	1	1	1	1	1	1	1	1	1	1	1	1		1	1	1	1	1	1	1	1			1	1	1	1	1	1	1	1	1	1	0.5	1	1	0	0.5	1	0	1	1	1	1	1
30	1	1	1	0	1	1	0.5	1	1	1	1	1	1	1	1	1	1	1	1	1	1	1			1	1	1	1	1	1	1	1	1	1	1	1	1	0	1	1	1	1	1	1	1	1
31	1	0	1	1	0	1	1	1	1	1	1	1	0	1	1		1		1	1	0	1		1	1	1	0.5		0	0	0.5	1	1	1	1	0	1	0	1	0.5	0	0	1	1	1	1
32	1	0	0.5	1	1	1	1	1	1	1	1	1	1	1	1		1	1	1	1	0.5	1	0.5	1	0.5	1	0.5	0.5	0.5	0	1	1	1	1	1	1	1	0	1	1	0.5	1		1	1	1
33	1	1	1	1	1	1	1	1	1	1	1	1	1	1	1	1	1		1	1	1	1	1	1	1	1	1	0.5	1	1	1	1	1	1	1	1	0.5	0	1	1	0	1		1	1	1
34	1	0.5	1	1	1	1	0.5		1	1	0	1	1	1	1	1	1	1	1	0	0	1	1	0	0.5	0	0.5	0.5	0	0	1	1		0.5	1	0	0	0		0.5	0	0		1	1	
35	1	0	0.5	0	0.5	1	1	1	1	0	0	1	0	1	1		1	1	1	0	0.5	1	10.5	1	1	1	0	1	0.5	0	1	1	0	1	1	1	1	0	1	0	1	1	1	1	1	1
36	1	1	1	0	1	1	1	1	1	1	0	1	1	1	0		1	1	1	0	0.5	1	0		1	1	1	1	1	1	1	1	1	1	0	1	1	0	1	1	0	1		1	1	
37	1	0.5	1	1	1	1	0.5	1	1	1	1	1	0	1	1		1	1	1	1	0.5	1	1	1	1	1	1	1	1	1	1	1	1	1	0		0	0	1	0.5	0.5	1	0.5	0.5	1	1
38	1	0.5	1	0.5	0	1	1	1	1	0.5	1	1	1	1	1		1	1	1	1	0.5	1	0	1	0.5	0.5	0.5	1	1	1	1	1	1	1	0	1	1	0	1	1	0	1	1	1	1	1
39	1	0	1	1	0	1	1		1	1	0	1	1	1	1		1	1	1	0	0.5	0	0	1	1		1	1	1	1	1	1	1	1	0	1	1	0	1	0.5	1	1		0.5	1	
40	1	0	1	0	1	1	1		1	1	0	1	1	1	0		1	1	1	1	0.5	1	0.5		1	1	1	1	1	1	0.5	1	1	1	1	1	1	0	1	1	1	0	0.5	0.5	1	
41	1	0	1	0.5	0.5	1	0.5	1	1	1	1	1	0	1	1		1	0.5	1	0	0.5	1	1	0		1	0	1	0	0	1	1	0.5	1	0	1	1	0	1	0.5	1	1	1	0.5	1	
42	1	0.5	1	0.5	1	1	1		1	1	1	1	1	1	1		1		1	1	0.5	1	1		0.5	1		1	0		1	1		1	0	1		0	1	-	1	0			0.5	1
43	1	0	1	0.5	1	1	0.5	1	1	1	0	1	1	1	1		1	0.5	1	1	0.5	1	1	1		1		1	0.5	0.5	0.5	1	1	1	0.5	1	0	0	1	0.5	1	0	1	1	1	1
44	1	0.5	1	1	1	1	1	1	1	1	1	1	1		1	1	1		1	1	0.5	1	1	1	1	1		1	0	0	1	1	1	1	1	1	0	0	1	0.5	1	1	1	0.5	1	
45	1	0.5	1	0	0	1	1	1	1	0.5	0.5	1	0		1	1	1		1	1		1	1	1	1	0.5	0.5		0	0		1		0	0	1	0	0	0.5	0.5	1	1	1	0	1	
46	1	1	0.5	0.5	1	1	1	1	1	1	1	1	1		1	1	1	1	0.5	1	0.5	1	1	1	1	1	1	1	0	0	1		1	1	1	1	1	0	1	0.5	1	0	1	1	1	1
47	1	0.5	1	0.5	0	1	1	1	1	1	1	1	1	1	1	1	1		1	0	0.5	1	1		1	1	0.5	1	0	0	1	1	1	1	0	1	1	0	1	0.5	1	1	1	0.5	1	1
48	1	0	1	1	0.5	1	0.5	1	1	1	1	1	0		1	0	1	1	0.5	1	1	1	0.5	0	0.5		0	1	0	0		1	0.5	1	1	0.5	1	0	0.5	0.5	1	1	1	0	1	
49	1	0.5	1	0	0	1	1	1	1	1	0	1	1	1	1	0	1		1	1	0.5	1	1		1	1		1	0	0	1	1	1	1	0	1	0	0	0.5	0.5	1	0	0.5	1	1	
50	1	0	1	1	0	1	1	1	1		1	1	0	0	1		1	1	1	1	0	0	0.5		1	1	0.5	0.5	0	1		1	1	1	0.5	0.5	0	0	0.5	0.5	1	1		1	0	
51	1	0	1	0	0	1	1	1	1	0.5	1	0	0.5	0	1	0	1		0	0	0.5	0	00.5	1	1	0		0.5	0	1		1		1	0	1	0	0	0.5	0.5	1	1		1	1	
52	1	1	1	0.5	0.5	1	1	1	1	1	0	1	1	1	1	1	1		1	1	0.5	1	1		1	1	0.5	1	1	1		1	1	1	1	1	1	0	1	1	1	0		1	1	1
53	1	1	1		0	1	1	1	1	1	1	1	1	1	1	1	1	1	1	1		0	1	1	1	1	1	1	1	1	1	1	0.5	1	0.5	0.5	0	0	1	1	1	1	1		1	1
54	1	1	1	0.5	0.5	1	1	1	1	1	1	1	1	1	1	1	1		1	1	0.5	1	0	1	0	1	1	1	1	1	1	1	1	1	1	1	1	0	1	1	1	1	0	1	1	1
55	1	0.5	1	0.5	0	1	1	1	1	1	1	1	1	1	1	1	1	1	1	1	0.5	1	1	1	0.5	1	1	1	1	1	1	1	1	1	1	1	1	0	1	1	1	0	1		1	
56	1	0	1	0	0	1	0.5	1	1	0.5	0.5	0	0	0	1			0.5	1	1	0.5	0	1	1	1	1	0	0.5			0.5	1	0.5	1	0.5	0.5	0.5	1	0.5	0.5	1	1	1		1	
57	1	0	1	0	0.5	1	1	1	1	1	1	1	1	1	0		1		1	1	0	0.5	0.5		1	1		0.5			0.5	1	0.5	1	0	1		0	1	0.5	1	0	0.5		1	1
58	1	1	1	0	0.5	1	1	1	1	1	1	1	1	0.5	0		1	1	1	0	0.5	1	0	1	1	1		1	1	1	1	1	1	1	1	1	1	0	1	1	1	1	1	1	1	1
59	1	1	0.5	0.5	1	1	1	1	1	1	0	0	1	0	0		1	1	1	0	0	0	0.5		0.5	1	1	0.5	0.5	0.5	1	1	1	1	0	1	1	0	1	0.5	1	1	0.5	1	1	
60	1	1	1	0	0	1	1	1	1	1	0.5	1	1	1	1		1		1	0	0.5	1	0	1	1	1		1	0	1	1	1	1	1	0	1	0.5	0		0.5	1	1	1	1	1	1
61	1	0	1	1	0.5	1	0.5	1	1	1	0	1	1	1	1		1	1	1	1	0.5	1	0	1	1	1	1	0.5	1	1	0.5	1	1	1	1	1	1	0	1	0.5	1	0	1		1	1
62	1	0	0.5	1	1	1	0.5	1	1	1	1	1	1	1	1		1	1	1	1	0.5	1	1	1	1	1	0.5	0.5	0	0	1	1	1	1	1	1	0.5	0	1	1	1	1	0.5	0.5	1	1
63	1	1	1	0	1	1	1	1	1	1	1	1	0	0	1		1		1	1		0	0	1	1	1	1	1	0	0	1	1	1	1	1	1	1	0	1	0.5	1	1	1	1	1	1
64	1	0	0.5	0.5	0.5	1	0.5		0	0.5	1	1	1	0	0	1	1		1	1	0.5	0	0.5	1	0.5	0.5	0.5	0.5	1	1	0.5	1	1	1	1	0	1	0	1	1	1	1	1	1	1	1
65	1	0	1	0	0	1	1	1	0	0.5	1	1	1	0.5	0	1	1	1	1	0	0	1	1	0	0.5	1	1	0.5	0	0	0.5	1	1	1	1	1	1	0	1	0.5	1	1	0	1	1	1
66	1	1	1	0.5	1	1	1	1	1	1	0	1	0.5	1	1		1	1	1	0	0.5	0	0.5	1	1	1		1	0	0	1	1	0.5	1	1	1	1	0	1	0.5	1	1	1	1	1	1
67	1	1	1	1	1	1	1	1	0	0	1	1	1	1	0	1	1		1	1	0.5	1	1	1	1	1	0	1	0.5	0.5	1	1	1	0	1	1	0	0	1	0.5	1	0		1	1	1
68	1	0	1	0	0	0	1	1	1	1	0	1	1	0	1	1	1		1	1	0.5	1	0	1	1	1		1	0	0		1	1	1	0	1	0	0	1	0.5	1	1		0	1	1
69	1	0	1	1	1	1	1	1	1	1	1	1	1	0	1		1		0.5	1	0.5	1	0.5	1	0.5	1	1	1	1	1	0.5	1	1	1	0	1	0	0	0.5	1	1	1		1	1	
70	1	1	1	1	1	1	1	1	1	1	1	1	1	0	1		1	1	0.5	1	1	1	0.5	0	1	1	1	1	1	1		1		1	0	0.5	0	0	0.5	1	1	1		1	1	
71		0	1	1	1	1	1	1	1	0.5	1	1	0	0	1	1	1	1	1	0	0.5	1	1	1	1	1	1	1	1	1	1	1	1	1	1	1	1	0	1	1	1	1	0	1	1	1
72	1	1	1	0.5	0	0	0.5	1	1	0.5	0	1	0.5	1	1	0.5	1	1	1	0	0.5	0	0		0.5	1	1	1	0	0	0.5		1	0	1	1	1	0	1	0.5	1	0	1	0	1	
73	1	0	0.5	1	0.5	1	0.5	1	1	1	1	1	1	0	1	1	1	1	0.5	0	0.5	1	0	1	1	1	1	1	1	1	1	1	1	1	1	0.5	1	0	1	1	1	0	1	1	1	
74	1	0	1	1	0	1	1	1	1	1	1	1	1	1	1	1	1	1	1	0	1	1	0		1	1	0.5	1	0	1	0.5	1	1	1	0	0.5	1	0	1	0.5	0	1	1	1	1	
75	1	1	1	1	0	1	1		1	1	1	1	1	1	1	1	1	1	1	1	0.5	1	0.5	1	1	1	1	0.5	0.5	0.5	1	1	1	1	0	1	1	0	1	0.5	1	1		1	1	
76	1	1	1	0.5	0	1	0.5	1	1	1	1	1	1	1	1	1	1	1	1	1	0.5	1	1		1	1	0.5	1	0	0	0.5	1		1	0	1	1	0	1	0.5	1	1	1	0	1	
77	1	0	0	1	1	1	1	1	1	0.5	0	1	1	1	1	0	1	1	1	1	0	1	0.5		0.5	1	1	1	0	0	1	1		1	0	1	0	0	0.5	0.5	1	0.5	0	0	1	
78	1	1	1	1	1	1	1	1	1	1	1	1	1	0	1	1	1	1	1	0	0.5	1	0.5		1	1	1	1	1	1	1	1	1	1	0	1	1	0	1	0.5	1	1	1	1	1	1
79	1	1	1	1	1	1	1	1	1	1	1	1	1	1	1	1	1	1	1	1	1	1	0.5		1	1	1	1	0	0	1	1		1	1	1	1	0	1	0.5	1	0	1	1	1	1
80	1	0	0.5	1	0	0.5	1	1	1	1	1	1	0.5	0.5	1		1	1	1	1	0.5	1	0.5			1	1	1	0.5		0.5	1	1	1	1	0.5	1	0	1	0.5	0	0		1	1	
81	1	1	1	0.5	1	1	0.5	1	1	1	0	1	0.5	0	1	1	1	1	1	0	0.5	1	0.5		1	1	1	0.5	0	0	1	1	1	1	0		0	0	1	0.5	1	1	0	1	1	
82	1	0	1	1	1	1	0.5	1	1	1	0.5	1	1	1	1	1	1	1	1	0	1	0	0.5		1	1	1	0.5	1	1		1		1	1	1	1	0	1	1	1	1		1	1	
83	1	1	1	0	0	1	0.5	1	1	1	1	1	1	1	1	1	1	1	1	1	0.5	0.5	1		0.5	1	1	1	0	0	1	1		1	0	1	0	0	1	0.5	1	1	1	0	1	1
84	1	0	1	0.5	1	1	0.5	1	1	1	0	1	1	0	1	1	1		0.5	0	1	1	0.5		0	1	0.5	1	1	1	0.5	1	1	1	1	0.5	0	0	1	0.5	1	1	0	1	1	1
85	1	0	1	0.5	1	1	1	1	1	1	1	1	1	0	1	1	1		0.5	1	0	1	0.5		0.5	1	0.5	0.5	1	1	0.5	1	1	1	1	1	0	0	1	0.5	1	1	1	1	1	1
86	1	1	1	1	1	1	0.5	1	1	1	1	1	1	1	1	1	1	1	1	1	0.5	1	1		1	1	1	1	0.5	0	1	1		1	0	1	1	0	1	0.5	1	1	0	1	1	
87	1	0	0.5	1	1	1	1		1	1	0	1	0.5	0	0	0	1		1	0	0	1	0		.	1	1	1	0	0	1	1	1	1	0	0.5	1	0		1	1	1	0		1	
88	1	0	0.5	1	1	1	1	1	1	1	1	1	1	1	1	1	1		1	1	1	0	0.5		1	1	0.5	1	0	0	1	1	1	1	0	1	0	0	0.5	0.5	1	0			1	1
89	1	0	0.5	1	0.5	1	1	1	1	1	1	1	1	0	1	0	1		1	0	0.5	1	0.5		1	1	1	1	0	0	1	1	1	1	1	0.5	0.5	0	0.5	0.5	1	1	1	0	1	
90	1	0	0.5	1	1	1	0.5	1	1	1	1	1	1	1	1	0	1	1	0.5	0	0	1	0.5		1	0	0.5		0	0		1	1	1	0	0.5	1	0	1	0.5	1	1		1	1	
91	1	0	1	1	1	1	1		1		1	1	1	1	1				1	0	0	0	0			1	1	0				1	1	1	0	0.5	0	0	0.5	1	0	0		1	0.5	
92	1	0	1	1	1	1	1		1	1	0.5	1	1	0.5	1	0	1		1	1	0.5	1	0.5		0.5	1	0.5	0.5	0		0.5	1	1	0.5	0	0.5	1	0	1	0.5	1	1	0	0	1	
93	1	1	0	1	1	1	1	1	1	1	1	1	0.5	0	1	0	1		1	0	0.5	0	0		1	1	1	0.5	0	0		1		1	0	0.5	1	0	1	0.5	0	1	0	0.5	1	
94	1	0.5	0.5	1	1	1	1		1	0	1	1	1	0	1	0	1		0.5	1	0	0	0.5		0	0	0.5	0.5	0	0		0.5	0.5	0	0	0.5	0	0	1	0.5	1	0	0		1	
95	1	0	1	0	0.5	1	1		1	0.5	1	1	1	1	1	1	1	0.5	1	1	0.5	0	0.5		1	1	1	0.5	1	1	1	1	1	0	0	0.5	1	0	1	0.5	1	0	1	1	1	
96	1	0	0.5	1	0.5	1	1	1	1	0.5	1	1	1	1	1		1		1	1	0.5	0.5	0.5	1		1	1	0.5	1	1		1	1	1	0		1	0		1	0.5	0		1	1	
97	1	0	1	0	1	1	1		1	1	1	0	1	1	1	1	1		0.5	1	0.5	1	0.5	1	0	1	0.5	0.5	0.5	0.5	1	1	1	1	1	1	1	0	1	1	1	0	0	1	1	1
98	1	0	1	1	1	1	1		1	1	1	1	1	1	1	1	1	0.5	1	1	0	0	0.5		0.5	1	1	0.5	0.5			0.5	1	1	0	0.5	0	0	0	1	1	0		1	0.5	
99	1	1	1	1	1	1	1			1	1	0.5	1	0.5	1	1	1	0.5	1	1	0.5	1	0.5		1	1	1		1	1		1	1	1	0	0.5	1	0		1	0.5	0		1	1	
100	1	1	1	0	1	1	1		1	1	0	1	1	1	1	1		1	1	1	0	1	0.5		1	0.5	1	0.5	1	1	1	0.5	1	1	0.5	1	1	0		0.5	1	0	1	1	1	
101	1	0.5	1	1	1	1	1	1	1	1	1	1	1	1	1	1			1	1	0	1	0.5		1	0.5	1	1	1	1	1	0	1	1	1	1	1	0	0.5	0.5	1	0		1	1	
102	1	1	1	1	1	1	1	1	1	1	1	0.5	1	1	1	1	1	1	1	0	1	1	0.5		0.5	1	1	1	1	1	1	0	1	1	1	0.5	1	0	1	0.5	0	0	0	1	1	
103	0.5	0.5	1	0	0	1	0	1	1	0	1	1	0	0	1	1		0.5	0.5	1	0	1	0		0.5	0	0.5	1	1	1		0	0.5	0.5	1	0	1	0	1	0.5	1	0	1	0	0.5	
104	1	0	1	1	1	1	1		1	1	1	1	1	1	1	1		0.5		1	0	1	0.5		1	1	1	1	1	1		0	1	1	0	1	1	0	1	0.5	1	0	0	1	1	
105	1	0.5	1	0	0.5	1	1		1	1	1	1	0.5	1	1	1	1	1	1	1	0.5	1	0		0	1	1	1	1	1	1	1	1	1	0	1	1	0	1	0.5	1	0	0	1	1	1
106	1	0	1	1	1	1	1		1	1	0	1	1	0.5	1	1	1		1	0	0.5	0	0.5		0.5	1	0.5	1	0	0	1	1	1	1	0	1		0	1	0.5	0.5	0			1	
107	1	0	0.5	1	0	1	0	1	1	0.5	0.5	1	0.5		1	1	1		0.5	0	0	1	0.5		1	1	1	1	1	1	0.5	1	1	1	0	0	0	0	1	0.5	1	0	1	0	0.5	
108	1	0	1	1	0	1	1	1	1	0.5	1	1	1	1	1	1	1		1	1	0	1	0.5		0.5	1	1	1	0.5	0.5	1	0.5	0.5	1	1	0.5	1	0	1	0.5	1	0		1	0.5	
109	1	0	0.5	0	1	1	1	1	1	0.5	0.5	1	0.5	1	1	0		1	1	0	0	0	0.5		0	1	0.5	0.5	1	1		1	1	0.5	0	0.5	1	0	1	0.5	1	0	1		1	
110	1	0	0.5	1	1	1	1	1	1	0.5	1	1	1		1	1		1	1	0	1	1	0.5		1	0	0.5	0.5	1	1	1	1	1	0.5	1	1	1	0	0.5	0.5	1	0	0		1	
111	1	1	0	0	1	1	1	1	1	1	1	1	1	1	1	1	1	1	0.5	1	0	1	0.5		0.5	1	1	0.5	1	1	0.5	1	0.5	1	0.5	0.5	1	0	1	0.5	1	0		1	1	
112	1	0	1	1	0.5	1	1	1	1	1	1	1	1	1	1	1	1	1	0	1	0.5	1	0.5		1	1	1	1	0	0	0.5	0.5	1	1	0	1	0	0	1	0.5	1	0	0.5	1	1	1
113	1	0	1	1	1	1	1	1	1	1	1	1	1	1	1	1	1		1	0	0	1	0.5		1	1	1	1	1	1		1	0.5	1	0	1	1	0	0.5	0.5	1	0		1	1	1
114	1	1	0.5	0	0	1	0	1	1	1	1	1	1		1	1			0.5	1	0	1	0.5		0.5	1	1	1	1	1	1	0	1	0.5	0	1	1	0	1	1	0	0		1	0.5	

Scores (n=91), columns 47–91

Research Questions (n=205)	47	48	49	50	51	52	53	54	55	56	57	58	59	60	61	62	63	64	65	66	67	68	69	70	71	72	73	74	75	76	77	78	79	80	81	82	83	84	85	86	87	88	89	90	91
1	1	1	0	0.5	1	1	1	0	1	1	1	1	1	1	1	0.5	1	0.5	1	1	0.5	1	0	1	0	0	0.5	0	1	1	0.5	0.5	1	1	0	0.5	1	1	0.5	1	1		1	1	1
2	0.5	1	1	1	1	1	1		1	1	0	1	1	0.5	0.5	1	1	0	1	1	0.5	1	0	1	0	1	0.5	0	1	1	0.5	0.5	1	1	1	1	0	1	0.5	1	0.5	1	0.5	1	0.5
3	1	1	0	1	1	1	1		0.5	1	1	1	1	1	0	0.5	0	1	1	1	0	1	1	1	1	1		1	0.5	1	0.5	0.5		1	0	0.5	1	1		1	0.5	0	0.5	1	0.5
4	1	1	0	1	1	1	1	0	1	1	1	0	1	1	1	0.5	1	1	1	0.5	0.5	0.5	1	0.5	1	1	0.5	0	1	1	0.5	0.5	1	1	1	1	1	0.5		1	1	1	1	1	1
5	1	1	0	0	1	1	1	1	1	0.5	1	1	1	0	1	0.5	1	1	1	1	0	1	1	1	1	1	1	1	1	1	1	1	1	1	1	1	1	1	1	1	1		1	1	1
6	1	0.5	1	0.5	1	0.5	0.5	1	1	1	0.5	1	1	1	1	0.5	0	1	1	1	0.5	1	1	1	1	1	0.5	1	1	1	1	1	1	1	1	1	1	1	1	1	1		0.5	1	0.5
7	0	1	1	1	1	1	1	0.5	1	1	1	1	1	1	0	0.5	1	1	1	1	0.5	1	1	1	1	1	1		1	1	1	1	1	1	1	1	1	1	1	1	1		1	1	1
8	1	1	1	1	1	0.5	0.5	1	1	0.5	0.5	1	1	1	1	0.5	1	0	1	1	0.5	1	1	1	1	0	1	0	1	1	1	1	1	1	1	1	1		1	1	1		0.5	1	1
9	0	1	1	1	1	0.5	0.5	0.5	1	0.5	1	0.5	1	1	1	1	1	0	1	1	0.5	1	1	0.5	1	0	1	1	0.5	1	1	1		1	1	1	1	1	1	1	1		1	1	1
10	1	1	0	1	1	1	1	0.5	1	0.5	1	1	1	1	1	0.5	1	1	1	1	1	1	1	1	1	1	1	1	1	1	1	1	1	1	1	1	1	1	1	1	1		1	1	1
11	1	1	1	1	1			1	1	0.5		0.5	1	1	1	1	1	1	1	0.5	1	1	1	1	1	0.5	1	1	0.5	1	1	0.5	1	1	1	1	1	1	1	1	1		1	0	1
12	1	1	1	1	1	1	1	1	1	0.5	1	1	1	1	1	0.5	1	0	1	1	1	1	1	1	1	1	1	1	1	1	1	1	1	0	0	1	0	1	1	1	1		1	1	1
13	1	1	0	1	1	1	1	1	1	1	1	1	1	0.5	1	0	1	1	1	1	0.5	1	1	1	1	1	1	1	1	1	1	1	1	1	1	1	1	1	1	1	1		1	1	1
14	1	1	1	1	1	1	1	1	0.5	0.5	0.5	1	1	1	1	0.5	1	1	1	1	1	1	1	1	1	1	1	1	1	0.5	0.5	0.5	1	1	1	0	0.5	1	1	1	1		1	1	1
15	1	1	0	1	1	0.5	0.5	1	1	1	1	1	1	1	1	0.5	1	1	1	0.5	1	1	1	1	1	1	1	1	0.5	1	1	1	1	1	1	1	1	1	1	1	1		1	1	1
16	0.5	1	0.5		1	1	1		1		1		1	0	0	1	1	0.5			1		0	1	0	0.5		0	1	1	1	0.5	0.5	1	0	1	0.5		1	1	1		1	1	0
17	1	0	0		1			1	1	1	1	1	1	1	1	1	1	0.5	1	1	1	0.5	0	1	1	1	1	1	1	1	1	0	1	1	0	0.5	0.5	1		1	1		1	1	1
18	1	1	0	1	0.5			1	1	1	1	1	1	1	1	0.5	1	1	1	0.5	1	1	1	0.5	1	0	1	0	1	1	1	1	1	1	1	1	0	1	1	1	1		0.5	1	1
19	1	1	1	1	1	0.5	0.5	1	1	1	0.5	1	1	1	0.5	0.5	1	1	1	1	1	1	1	1	0	0	1	1	0.5	1	0.5	0.5	1	1	1	1	0	1	1	1	0		0.5	1	0.5
20	1		1	1	1				1	0.5		1	1	0.5	0.5	0.5	1	0	1	0	0.5	1	1	0.5	1	0	1	1	0.5	1	1	1	1	1	0	0.5	1		1	1	0		0	1	0
21	1	1	0	1	0.5	1	1	1	1	1	1	1	1	1	0.5	0.5	1	1	1	1	0.5	1	1	1	1	1		1	1	1	1	0.5	1	1	1	1	1	1	1	1	1		1	0.5	1
22	1	1	0		1	1	1		1	1	1	1	1	1	0.5	0.5	1	1	1	1	0	1	1		0	0.5		1	1	1	1	1	1	1	1	1	1	1	1	1	0.5		1	0.5	1
23	1	0.5	1	1	1	1	1	1	1	1	1	1	1	1	1	1	1	1	1	1	1	1	1	1	1	1	1	1	1	1	1	0.5	1	1	1	1	1	1	1	1	1		0.5	0.5	1
24	1	0.5	1		1	1	1	1	1		1	1	1	1	1	1	1	1	1	1	0.5	1	1	1	1	1	1	1	1	1	0.5	0	1	1	1	1	0.5	1	1	1	1		1	1	1
25	1	0.5	1		1	1	1	1	1		1	1	1	1	1	0.5	1	0	1	0	0.5		1	1	1	1	1	1	1	1	1	0.5	1		0	1	0	1	1	1	1		1	1	1
26	1	1	0	1	1			1	1				1	0	1	0.5	1	1	1	1	1		1	0.5	1	0	1	1	1	1		0.5	1	1	0	1	0		1	1	1		0.5	1	1
27	1		0	0	1				1	1	1		1	0	0.5	0.5	0	1	1	0	1	1	0	0	1	0		1	0.5	1	1	1	1	1	1	0	0.5		1	0	0.5		0.5	0.5	0
28	1	1	1	1	1	1	1	1	1	1	1	0.5	1	1	1	1	1	1	1	1	1	1	1	1	1	1	1	1	1	1	1	1	1	1	1	1	1	1	1	1	1		0.5	1	1
29	0.5	1	1	1	0.5	1	1	1	1	1	1	1	1	1	1	1	1	1	1	1	0.5	1	1	1	1	1	1	1	0.5	1	1	1	1	1	1	1	1	1	1	1	1		1	1	1
30	1	0.5	1	1	1	1	1	1	1	1	1	1	1	1	1	1	1	0.5	1	1	1	1	1	1	1	1	1	1	1	1	1	1	1	1	1	1	1	1	1	1	1		1	1	1
31	1	1	0	1	1	1	1		0.5	0.5		1	1	0	1	1	1	1	1	0.5	1		1	1	1	1	1		1	1	1	1	1	1	0	0	0	1	1	1	1		1	1	1
32	1	1	0	1	0.5	1	1	1		1		0.5	1	1	1	0.5	1	0.5	1	0.5	1	1	1	1	1	1	1	1	1	1	1	1	1	1	1	1	0	1	1	1	1		1	1	1
33	1	1	0	1	1	1	1	1	1	1	1	1	1	1	1	1	1	0.5	1	1	0.5	1	1	1	1	1	1		1	1	1	1		1	1	1	0.5		1	1	1		1	1	1
34	0.5	0.5	1	1	1	0.5	0.5			0.5	1	1	1	1	0.5	1	1	1		1	1		1	1	1	0	1		1		0.5	1	1	0	1	0.5	0		1	1	0.5		0.5	0.5	0.5
35	1	1	1		1	0.5	0.5	1	1	1	1	0.5	1	1	1	0.5	1	0	1	1	1	1	1	1	1	1	1	1	0.5	1	1	1	1	1	1	1	1	1	1	1	1		0.5	0.5	1
36	1	1	1		1	1	1	1	1	1	1	1	1	1	1	0.5	1	0	1	0.5	0	1	1	1	1	1	1	1	1		1	1	1	1	1	1	1	1	1	1	1		1	1	1
37	1	0.5	1	1	1			1	0	1	1	0.5	1	1	1	0.5	0	1	1	1	0.5	1	1	1	1	1	1		1	1	0.5	1	1	1	0	1	1	0.5	1	1	1		0.5	1	1
38	0	1	0		1			0.5	0.5	1	0.5	1	1	1	1	0.5	1	0.5	1	0.5	1	1	1	1	1	1	1	1	1	1	1	0.5	1	0	1	1	1	1	1	1	1		1	1	1
39	1	1	1	1	1	1	1	1	1	1	1	0.5	1	1	0	0.5	1	0.5	1	1	0	1	0	1	1	1		1	1	1	1	0.5	1	1	0	1	1	1	1	1	1		1	1	0.5
40	1	1	1		1	0.5	0.5	1	1	1	1	1	1	1	0.5	0.5	0	0	1	1	0	1	1	1	1	1	1		1	1	1	1	1	1	1	0.5	1	1	1	1	0.5		0.5	1	1
41	1	1	1	0.5	1			1	0.5	0.5	0	0.5	1	1	0.5	0	1	1	1	0.5	1	0	1	1	1	1	1	0	0.5	1	0.5	1	1	1	0	0	1		1	1	0.5		1	0.5	1
42	1	0.5	0	1	1			1	1	1	0	0	1	1	1	0	1	1	1	0.5	1	1	1		1		1	1	1	1	1	1	1	1	1	1	1		1	1	1		1	1	0.5
43	1	1	1		1			0.5	0.5	1	1	0.5	1	1	1	0.5	1	1	1	0.5	0	1	1	1	1	1	1		1	1	1	1	1	1	0	1	0.5	1	1	1	1		0.5	1	1
44	1	1	1	1	1			1	1	1	1	1	1	0.5	1	0.5	1	1	1	1	1	1	1	1	1	1	1	0	1	1	1	1	1	1	0	1	0.5	1	1	1	1		0.5	1	1
45	1	1	1		1				1	0.5		0	1	1	0.5	0.5	0	0.5	1	1	0.5		1	0.5	1	0	1	1	0.5	0.5	1	1	1	1	0	0	1		1	1	1		0.5	1	1
46	1	0.5	0	1	1	0.5	0.5	1	1	1	1	1	1	1	1	1	1	1	1	1	1		0	0.5	1	1	1		1	1	1	1	1	1	0	1	1	1	1	1	1		0.5	1	1
47	1	1	1	1	1			1	1	1		0	1	1	1	0.5	1	1	1	0.5	1		1	1	1	1	1	1	0.5	1	1	1	1	1	0	0	1	1	1	1	1		0.5	1	1
48	0.5	1	0	0	0.5	0	0	1	0.5	1	0	0.5	0.5	0.5	0	0	1	0.5	0.5	1	0.5	1	0	0.5	1	1	1	1			1	0.5	1	0	0	0.5	1	1	1	1	0.5		0.5	1	1
49	1	1	1	1	0.5	0	0	1		1		0.5	1	0.5	0.5	0.5	1	1	1	0.5	1	1	0	1	1	1	1	1	1	1	1	1	1	1	0	1	0		1	1	1		0.5	1	1
50	1		1	0	1	0	0					1	1	1	0.5	1	1	0.5	1	1	0.5	1	1	1	1	0	0.5	1	0	1	1	0.5	1	0	0	0	1		1	1	1		0.5	0.5	0
51	0	1	1	0	1	0	0		1		0	1	1	1	0.5	0	0	0.5	1	0.5	0.5	0.5	0	1	1		0.5	1	0	0.5	1	1	1	1	1	1	1		1	1	0.5		0.5	1	0
52	1	1	1	0	1			1	1		1	1	1	1	0	0	1	1	1	1	1		1	1	1	1	1	1	1	1	1	1	1	1	1	1	1		1	1	1		1	1	1
53	1		1		0.5							1	1	1	0.5	0	1	1	1	1	1		1		1		1	0	0.5	1	1	1	1	1	0	1	1		1	1			1	1	1
54	0	0.5	1	1	0.5	0.5	0.5	1	1	1	1	0.5	1	1	0	0	0	1	1	1	1		1	1	1	1	1	1	0	1	1	1	1	1	1	1	1	1	1	1	1		0.5	1	1
55	1	1	1	1	1	0.5	0.5	1	1	1	1	1	1	1	1	0.5	0	1	1	1	1	1	1	1	1	1	1	1	1	1	1	0.5	1	1	1	1	0.5		1	1	1		1	1	1
56	1		0	1	0.5			1				0		1	0.5	0	1	0.5	1	0.5	1	0	1	1	1	0	1	0	0.5	0.5	1	1	1	1	0	0.5	0		1	1	1		0.5	1	0
57	1	0.5	1		0.5			1				0	1	0	1	0.5	0	0	1	0.5	1		1	1	0	1	1	1	0.5	1	1	1	1	1	1	1	0		1	0	0.5		0.5	0	0
58	1	0.5	0		1			1	1	0.5	1	1	1	1	0	1	1	1	1	1	1	1	1	1	1	0	1	1	1	0.5	1	1	1		1	1	1		1	1	1		0.5	1	1
59	1	0	1		1					0.5	1	1	1	1	0	0.5	0	0.5	1	1	1	1	0	1	1	0	1	1	0.5	0.5	1	1	1	1	1	1	1		1	1	1		1	1	1
60	1	1	1		1			1	0	0.5	0	0	1	1	0.5	0.5	1	1	1	0.5	1	0	1	0.5	1	1	1	1	1	1	1	1	1	1	0	1	1		1	1	1		1	1	1
61	1	1	1	1	1	1	1	1	1		1	1	1	1	0	0.5	0	0	1	1	1	1	1	1	1	1	1	1	0.5	1	1	1	1	1	1	0.5	0		1	1	1		0.5	1	0.5
62	1	1	1	1	1	1	1	1		1	1	1	1	1	1	0.5	1	1	1	1	1	1	0	1	1	1	1	1	1	1	1	1	0.5	1	1	1	1	1	1	1	1		0.5	1	1
63	1	1	1	1	1	1	1	1		0.5	0	1	1	0.5	0.5	0	1	1	1	0.5	1	1	1	1	1	1	1	1	1	1	0.5	1	1	1	0	1	1	1	1	1	1		0.5	1	1
64	1	1	1	1	1					1	1	1	1	1	1	0.5	0	0.5	1	1	1	1	1	1	1	1	1	1	1	0.5	1	1	1	1	0	1	1	1	1	1	1		1	1	1
65	1	1			1	1	1	1	1	1	0	0.5	1	1	1	0.5	0	0	1	0	0	1	0	1	1	1	1	1	0.5	1	1	0.5	1	1	0	1	0.5	1	1	1	1		0.5	1	1
66	1	1	1	0	0.5	0.5	0.5		1	1	1	1	1	1	1	0.5	1	1	1	0.5	1	1	1	1	1	0	1	1	1	1	1	1	1	1	1	1	1	1	1	1	1		0.5	1	1
67	1	0.5	0		1	1	1	1	1	1	1	1	1	1	1	1	1	0.5	0.5	1	1	1	1	1	1	0	1	1	0.5	1	1	1	1	1	1	0.5	1	0	1	1	1		0.5	1	1
68	1		1	1	0.5			1	1			1	1	1	0.5	1	1	0	1	0	1	1	1	1	1	1	1	1		0.5	1	1	1	1	0	0.5	0		1	1	1		0.5	1	0.5
69	1	1	1	1	0.5	1	1	1	1	1	1	0.5	1	1	0	0.5	0	0.5	1	0.5	1	1	0	1	1	1	0.5	1	0.5	1	1	1	1	1	0	1	0	1	1	1	0		1	1	0
70	1	1	1		0.5			1	1		1	1	1	1	1	0.5	1	1	1	1	1		0	1	1	1	1	1	1		1	1	1	1	0	1	0	1	1	1	1		1	1	1
71	1	1	1	0	0.5			1	0.5	0.5	0	1	1	1	1	1	1	0.5	1	1	1		1	1	1	1	1	1	1	1	0.5	1	1	1	1	1	1	1	1	1	0		1	1	1
72	1	1	1	0	0.5			1	1	0.5	0	0	1	0	0	0.5	0	0.5	1	1	0.5		0.5	1	1	0	1	1	0.5	1	1	1	1	1	0	0.5	1		1	1	0		0.5	0.5	0.5
73	1	0.5	1	1	1	1	1	1	1	0.5	1	1	1	0.5	0.5	0	1	0.5	1	1	0.5	1	1	1	1	1	1	1	1	1	1	1	1	1	1	1	1		1	1	1		0.5	1	1
74	1	1	1	1	1	1	1	1	0.5	1	1	1		1	0.5	0	1	0.5	1	1	0.5	1	0	1	1	1	1	1	0	0.5	1	1	1	1	0	1	0	0	1	1	0.5		0.5	0.5	0.5
75	1	1	1	1	1	1	1	1	1	1	1	1	1	1	1	1	1	1	1	1	1	1	1	1	1	1		1	1	0.5	1	0.5	1	1	1	1	1	1	1	1	1		1	1	1
76	1	0.5	0		1			1	1	0		0	1	1	1	0.5	1	1	1	1	0.5	1	0	1	1	1	1	1	1	1	1	1	1	0	0	1	1		1	1	0.5		1	1	1
77	1	0.5	0	1	0.5	0.5	0.5	1	1	0	1	1	1	1	0.5	0.5		0.5	1	1	1	1	1	0	1	0	1	1	0.5		1	1	1	1	0	0	1		1	1	1		1	0.5	1
78	1	1	1	1		0.5	0.5	1	1	1	1	1	1	1	1	0		1	1	1	1		0	0.5	1	1	1	1	1	1	1	1	1	1	0	1	0		1	1	1		1	1	1
79	1	1	1		0.5	1	1	1	1		1	1	1	1	1	1	1	1	1	1	0.5		0	1	1	1	1		1	1	1		1	1	0	1	1	1	1	1	1		1	1	1
80	1	0	1	0	1	1	1		1	1	0.5	1	0.5	1	0	0.5	1	0	0.5	1	0	1	1	1	1	0		1	1	0.5	1		1	1	1	1	0	1	1	1	1		1	1	1
81	1	0.5	1	1					1		0	1		1	0	0	1	0	1	0.5	1	1	1	1	1	1	1	1	1	0.5	1	0.5	1	0.5	1	1	0		1	1	1		0.5	1	1
82	1	1	1		0.5	0.5	0.5	1	1	0.5	1	1	1	1	1	1	1	0.5	1	1	0.5		1	1	0	1	1	1	1	1	1	1	1	1	0	1	1	1	1	1	1		0.5	1	1
83	1	1	0		0.5	0.5	0.5	1			1	0	1	1	1	0.5	0	0	1	0.5	1		0	1	1	0	1	1	1	1	1	0.5	1	0	0	1	0	1	1	1	1		0.5	1	1
84	0	1	0	0.5	1	1	1	1	1		1	1	1	1	0.5	0.5	1	1	1	1	0.5		0	1	1	1	1	1	1	1	1	1	0.5	0	0	1	0	1	1	1	0		1	1	0
85	1	1	0	0	1	1	1	1	1	1	1	1	1	0	0.5	0.5	0	1	1	0	1	0	1	1	1	1	1	1	1	0	1	0.5	1		1	1	1	1	1	0	0.5		1	1	1
86	1	1	0	1	1			1	1		1	1	1	1	0.5	0.5	1	1	1	0	1		1	1	1	1	1	1	1	1	1	1	1	1	1	1	1		1	1	0		1	1	1
87	0.5	1	0	1		0.5	0.5			1	0.5	1		0.5	0	0.5	1	1	0.5	0.5	0		0	1	1	1		1	0.5		1	0	1	1	1	1	0	1	1	1	0		0.5	1	0
88	0	1	0	1	0.5						1	0	1	1	0.5	0	1	1	1	0.5	0.5		0	1	1	0	1	1	0.5	0.5	1	1	1	1	1	1	1		1	1	0		0.5	1	0
89	1	1	0	1	1	0.5	0.5	1	1	1	1	1	1	1	0.5	0	1	0.5	1	0.5	0.5	0.5	1	1	1	1	1	1		1	1	1	1	1	1	1	1		1	1	1		0.5	1	0.5
90	1	1	0	0	1	1	1	1	0.5	1	1	1	1	1	0.5	0	0	0	1	0.5	0	1	1	1	1	0	1	1	0.5	1	0	1	1	1	0	0.5	1		1	0.5	1		1	1	1
91	0	0.5	0	0	1	0	0		1	1	0	0		1	0	0.5	1	1		0	0	1	0	0	0	1	1		1		0	0.5	0.5	1	0	0.5	0		1	0.5	0		1	1	1
92	1	1	0	1	1				1	1	1	0	1	1	1	1	1	1	1	0.5	0.5	1	1	1	1	1	0.5	0	0.5	0.5	1	1	1	0	0	1	0	1	1	1	0		0.5	1	0
93	0.5	1	0	1	0.5			0	1	0.5	1	1	1	1	1	0.5	1	0	1	0	0.5		0.5	1	0	1	1	0	0.5	1	1	0.5	1	0	1	1	0		1	1	0		0.5	1	0
94	0	0.5	0	1	1				1	0.5	0	1	1	1	0	0.5	1	1	0	0	0		0	0.5	0	1	0.5		1	1	0	5	1	1	0	0.5	0		1	0	0		0.5	0	0.5
95	1		1	1	1			1	1	0.5	1	1	1	1	0	1	1	1	1	0.5	0.5		1	1	1	0	1	1	1	1	1	0.5	1	1	1	1	1		1	1	1		1	1	0
96	1		0		1	1	1		1		1	1		1	0	0.5	1	1	1	1	1	1	1	1	1	0	0.5	1	0.5			0	1	1	1	1	0		1	1	0		1	1	0
97	1	1	1		1	1	1		1	0	1	1	0.5	1	0.5	1	1	1	1	1	1		1	1	1	0	1	1	1	0.5	1	0.5	1	1	1	1	1		1	1	1		1	0	1
98	1	0.5	1	1	0.5	0.5	0.5	1	1		0	1	1	1	0	0.5	1	1		0	0.5		1	1	1	0	0.5	1	0.5		1	0	1	1	0	0.5	0.5		1	0.5	0		0.5	1	0
99	1	1	1	0.5	1	0.5	0.5		1		1	1	1	1	1	0	1	0		1	0		1	1	1	1	1	1	1	0.5	1	0	1	1	1	1	0		1	0.5	0		1	1	0
100	1	1	1	0.5	1	0.5	0.5	1	1		1	1	1	1	1	0.5	1	1	1	1	0.5		1	0	1	0	1	1	1	0.5	1	0	1	1	1	1	1		1	0.5	1		1	1	1
101	1	0.5	0	1	1	1	1	1	1	1	1	1	1	1	0.5	1	1	1	1	1	1		1	1	1	1	1	1	0.5		1	1	1	1	1	1	0		1	1	1		1	1	1
102	1	1	1	0.5		1	1	1	1		1	1	1	1	0.5	1	1	1	1	1	1		1	1	1	0	1	1	0	0.5	1	0.5	1	1	1	1	0		1	1	1		0.5	1	0.5
103	1	1	0	0	1	0.5	0.5		1	1		1	1	1	0	0.5	0	0	0.5	0	1		1	1	0	0	1	1	0		1	0	1	0	0	1	1		1	0.5	1		1	1	0
104	1	1	1		1	1	1	1	1		1	1	1	1	0.5	0	1	0	0.5	0.5	0.5		0.5	1	1	0	1	1		1	1	0.5	1	1	1	1	1		1	0.5	1		1	1	1
105	1	1	0	1	1	0.5	0.5	1	1	1	1	1	1	1	0	1	1	1	1	1	1		1	1	1	0	1	1	1	1	1	1	1	1	1	1	1	1	1	1	1		1	1	1
106	1	0.5	1		1	1	1	1	1		0	1	1	1	0	0	1	1	1	1	0.5	1	0		1	1		1	1	1	1	0.5	1	1	1	1	0		1	1	1		1	1	0
107	1	0	0	0	0.5			1	0.5		1	1	1	1	0	1	1	1	1	0.5	0.5		1	1	0	1	1	1	1	1	0	1	1	0	0	0.5	0		1	1	0		0	0.5	0
108	1	0.5	0	1	1	0.5	0.5	1	1		1	1	1	1	0	0.5	1	0	1	0.5	0.5	1	1	1	1	0	1	1	0.5	1	1	0.5	1	0	1	0	0		1	0.5	1		0.5	1	0
109	1	1	0		1	1	1	1	1	1	1	1	1	1	1	0.5	1	1	1	0.5	0	1	0.5	0.5	0.5	1	1	1	0	0.5	0	1	1	1	0	1	0	1	1	1	1		1	1	1
110	1	0.5	0		1	1	1		1		1	1	1	1	0	1	1	0	1	1	0	1	1	1	1	1	1	1	0.5	0.5	1	1	1	1	1	0.5	1	1	1	0.5	1		1	1	1
111	1	1	1	1	1	1	1	1	1	1	1	1	1	1	0	1	1	1	1	1	1	1	1	1	1	1	1	1	0.5		1	1	1	1	0	1	1	1	1	1	0		1	1	0
112	1	1	0	1	0.5	1	1	1	1	0.5	1	1	1	1	1	0.5	1	1	1	1	1		1	1	1	0	1	1	1	1	1	1	1	1	0	1	1		1	1	1		1	1	!
113	0.5	1	1	1		0.5	0.5	1	1	1	1	1	1	1	0.5	0.5	1	0		0.5	0.5		1	0.5	1	0	1	1	1	1	1	0.5	1	1	1	1	1		1	1	0		1	1	0
114	1	0.5	0	0		0.5	0.5	1	1	0	0	1	1	1	0	0.5	0	1	1	0.5	1		1	0.5	0	0	1	1	1	0.5	1	0.5	1	0	1	1	0		1	1	1		0.5	1	0

	1	2	3	4	5	6	7	8	9	10	11	12	13	14	15	16	17	18	19	20	21	22	23	24	25
115	1	0	0.5	1	0	1	0	1	1	0.5	0	1	0.5	1	1	0	1		0.5	0	0.5	1	0		1
116	1	0	1	0	1	1	1	1	0.5	0.5	0	1	1	1	1	1	1		1	1	0	1	0.5	1	1
117	1	0	1	1	1	1	1	1	1	1	1	1	1	1	1	0	1		1	1	0	1	0.5		1
118	1	1	0	1	1	1	1	1	1	0.5	1	1	1	1	1	1	1		0	1	0	1	0.5		1
119	1	0.5	0.5	1	1	1	1	1	1	0.5	1	1	0.5	0.5	1	1	1		1	1	0	1	0		1
120	1	0	0.5	1	0	1	1	1	1	1	1	1	1	1	1	1	1		1	0	0	1	0.5		
121	1	1	1	0.5	0	1	0.5	1	1	1	0.5	1	1	1	1	1	1		0.5	0	0	1	1		0.5
122	1	1	1	0	1	1	1	1	1	1	1	1	1	1	1	1	1		1	0	0	1	1		0
123	0	1	0	0	0	1	0	1	1	0	1	1	0.5	0	0	1	1		1	0	0	1	0.5		0.5
124	1	0	1		0	1	1		1	0	0.5	0.5	1		0	1			0.5	0	0	1	0.5		0
125	1	1	0.5	1	0	1	1		1	1	1	1	0.5	1	1	1			0.5	0	0	1	0		0
126	1	1	1	0	0	1	1	1	1		1	1	0.5		1	1	0.5	1	1	1		0	0.5		0.5
127	1	0	0.5	1	1	1	1	1	1	1	1	1	1	1	1	1	1	1	1	1	0.5	1	0.5		1
128	1	0	0.5	0.5	1	1	0.5	1	1	1	1		0	1	1	1	1	1	1	0	0	1	0.5		1
129	1	0	1	1	1	1	1	1	1	1	1	1	1	1	1	1	1	1	1	1	0.5	1	0.5		1
130	1	0	0	0	1	1	1	1	0	0.5	0.5	1	0.5	1	1	0	0.5	1	1	0	0	0	0		0.5
131	1	1	0	1	1	1	1	1	1	1	1	1	1	1	1	1	1	0.5	1	1	0	1	0.5		1
132	1	0	1	1	1	1	1		1	0.5	1	1	1		0	1	0.5	1	0	1	0	1	1		
133	1	1	0.5		0.5	1	1					1	1	1	1	0	0.5	1	1	0	0	1	0.5		.
134	1	0	1	0.5	1	1	0.5	1	1	1	1	1	0.5	1	1	0	1	1	1	0	0	1	0.5		0.5

[illegible]

CRITERION 2: EFFECT ON THE OUTCOME — Based on the best current evidence and knowledge available in public domain: Would you say that the proposed research would have a measureable effect on the proposed outcomes?

Research Questions	1	2	3	4	5	6	7	8	9	10	11	12	13	14	15	16	17	18	19	20	21	22	23	24	25	26	27	28	29	30	31	32	33	34	35	36	37	38	39	40	41	42	43	44	45	46	47	48	49	50	51	52	53	54	55	56	57	58	59	60	61	62	63	64	65	66	67	68	69	70	71	72	73	74	75	76	77	78	79	80	81	82	83	84	85	86	87	88	89	90	91

[illegible]

34	1	0.5	0.5	0.5	1	1	0.5			1	0	1	0.5		1	1	[illegible]
35	1	0.5	0	0	0.5	1	0	1	1		0	1	0		0	1	[illegible]
36	1	1	0.5	0	1	1	0.5	1	1	1	0	1	1	0	1	1	[illegible]
37	1	0.5	1	1	1		0.5	1	1	1	1	1	0	1	1	1	[illegible]
38	1	0	0	1	0	1	0.5	1	1	0.5	1	1	0.5	1	1	1	[illegible]
39	1	0	1	1	0	1	1		1	0.5	0	1	1	0	1	1	[illegible]
40	1	0	0	0	0.5	0	0.5		1	1	0	1	1	1	1	1	[illegible]
41	1	0	0.5	0.5	0	1	0.5	1	1	1	1	1	0		1	1	[illegible]
42	1	0.5	0.5	0	1	1	1		1	1	1	1	1	0	1		[illegible]
43	1	0	1	0.5	0	0	0.5	1	1	1	0	1	0.5		1	1	[illegible]
44	1	1	1	0.5	1	1	1	1	1	1	1	1	0.5		1	1	[illegible]
45	1	0	1	0	0	1	0.5	1	1	1	0	1	0		1	1	[illegible]
46	1	1	1	0.5	0.5	1	1	1	1	1	1	1	0.5		1	1	[illegible]
47	1	0	1	0.5	0	1	0.5	1	1	1	1	1	1	1	1	1	[illegible]
48	1	0	0	1	0	1	0	1	1	0.5	1	0.5	0		1	0	[illegible]
49	1	1	1	0	0	1	1	1	1	0.5	0	1	1		1	0	[illegible]
50	1	0	0	1	0	1	1	0.5	1	0.5	0.5	1	0		1	0	[illegible]
51	0.5	0	0	0	0	1	1	0.5	1	0.5	0.5	1	0		1	0	[illegible]
52	1	1	1	1	0.5	1	1	1	1	1	0.5	1	0.5		1	1	[illegible]
53	1	1	0.5		0	1	0.5	1	1	0.5	1	1	1	1	1	1	[illegible]
54	1	1	1	1	0	1	1	1	1	0.5	1	1	1		1	1	[illegible]
55	1	1	0	1	0	1	1	1	1	1	1	0.5	1	1	1	1	[illegible]
56	1	0	0.5	0	0	1	0	1	1	1	0	0	0	0	1	0	[illegible]
57	1	1	0	0	0	1	0	1	1	0.5	1	1	0.5	1	1	1	[illegible]
58	1	1	1	0	0.5	1	1	1	1	1	1	1	1		1	1	[illegible]
59	1	1	1	0.5	0.5	1	0.5	1	1	0.5	0	0.5	0.5	0	1	1	[illegible]
60	1	1	0.5	0	0	1	0.5	1	1	0.5	1	1	0.5		1	1	[illegible]
61	1	0	0.5	1	0.5	1	0.5	1	1	0.5	0	1	1	1	1	1	[illegible]
62	1	0	0.5	0	0.5	1	0.5	1	1	0.5	0.5	1	1	0	1	1	[illegible]
63	1	1	0	0	1	1	0.5	1	1	1	1	0	0	0	1	1	[illegible]
64	0	0	0.5	0.5	0	1	0.5		0	0.5	1	1	0	0	1	1	[illegible]
65	1	0	0	0	0	0	0	1	0	0	1	0.5	0	0	1	0	[illegible]
66	1	1	0.5	0.5	0.5	1	1	1	1	1	0	1	0.5	0	1	1	[illegible]
67	1	1	1	1	1	1	1	1	0	0	1	1	0.5	0	1	1	[illegible]
68	1	0.5	0	0	0	0	0.5	1	1	0.5	0	1	0	0	1	1	[illegible]
69	1	0	0	1	1	1	1	1	1	0	1	1	1	0	1	0	[illegible]
70	1	1	1	1	0.5	1	0.5	1	1	1	1	1	1	0	1	1	[illegible]
71		1	1	0	1	1	0.5	1	1	0.5	1	1	0	0	1	0	[illegible]
72	1	1	0.5	0	0	0	0.5	1	1		0	1	0.5	1	1	0.5	[illegible]
73	1	0	1	1	0	1	0.5		1	1	1	1	1	0	1	1	[illegible]
74	1	0	1	1	0	1	1		1	1	1	0.5	0.5	1	1	1	[illegible]
75	1	1	1	1	0	1	1		1	1	1	1	0.5	1	1	1	[illegible]
76	1	1	0.5	0.5	0	1	0.5	1	1	0.5	1	1	0.5	0	1	1	[illegible]
77	1	0	0	0	0.5	1	0.5	1	1	0.5	0	1	0.5	0	1	0	[illegible]
78	1	1	1	0.5	1	1	1	1	1	1	1	1	1	0	1	1	[illegible]
79	1	0.5	1	1	1	1	1	1	1	1	1	1	1	1	1	1	[illegible]
80	1	0	1	1	0	1	1		1	0.5	1	1	0.5	0	1	1	[illegible]
81	0.5	1	0.5	1	0.5	1	0.5	1	1	0.5	0	1	0.5	0	1	1	[illegible]
82	1	0	1	0.5	1	1	0.5	0.5	1	1	0	1	1	0	1	1	[illegible]
83	1	1	0.5	0	0	1	1	1	1	1	1	1	1	1	1	1	[illegible]
84	1	0	1	0	1	1	0.5	1	1	0.5	0	1	1	0	1	1	[illegible]
85	1	0	0.5	0	1	1	1	1	1	0.5	0.5	1	0.5	0	1	1	[illegible]
86	1	1	1	0	1	1	1	1	1	0.5	1	1	1		1	1	[illegible]
87	1	1	0.5	1	1	1	0.5		0	1	0	0.5	0	0	1	0	[illegible]
88	1	0	1	1	1	1	0.5	1	1	1	1	1	1	1	1	0	[illegible]
89	1	0	0.5	0	0.5	1	0.5	1	1	0.5	1	1	0.5		1	0	[illegible]
90	1	0	0.5	0	1	1	1		1	1	1	1	0		1	0	[illegible]
91	1	0	0.5	1	1	1	0.5				1	1	1	1	1		[illegible]
92	1	0	0.5	1	1	1	1		1	0.5	0	1	0		1	0	[illegible]
93	1	1	0.5	1	1	1	1	1	1	0.5	1	1	0		1	0	[illegible]
94	1	1	0.5	1	1	1	0.5		1		1	1	0.5		1	0	[illegible]
95	1	0	1	0	0.5	1	1		1	1	1	1	0.5	1	1	1	[illegible]
96	1	0	1	1	0.5	1	1		1		1	1	1	1	1	1	[illegible]
97	1	0	1	0	1	1	0.5		1	1	1	0	1	1	1	1	[illegible]
98	1	0	0.5	0	0.5	1	0.5		1	0.5	1	1	0.5	1	1	0	[illegible]
99	0.5	0	0.5	0	0.5	1	0.5			1	0.5	0	1		1	1	[illegible]
100	1	0	0.5	0	1	1	0.5		0	1	0	1	1	1	1	1	[illegible]
101	1	0	0	1	1	1	0.5		1	1	1	0.5	1	1	1	1	[illegible]
102	1	0	0	1	1	1	0.5	1	1	0.5	1		1	1	1	1	[illegible]
103	1	0	1	0	0	1	0.5	1	1	1	1	1	1	1	1	1	[illegible]
104	1	0	0	1	1	1	0.5		0	0.5	0.5	1	1	1	1	1	[illegible]
105	1	0	1	0	0.5	1	1		1	1	0.5	1	0.5	1	1	1	[illegible]
106	1	0	1	1	0.5	1	1		1	1	0	1	1		1	1	[illegible]
107	1	0	0	0	0	1	0.5	1	1	0.5	0.5	1	0.5		1	1	[illegible]
108	0	0	0.5	0	0	1	0	1	1	0.5	0.5	1	1		1	1	[illegible]
109	1	0	0	0	1	1	1		0	1	0	1	0.5	1	1	0	[illegible]
110	1	0	0	1	1	1	0.5	1	1	1	1	1	1		1	1	[illegible]
111	1	0	0	0	1	1	0		1	1	1	1	1	1	1	1	[illegible]
112	1	0	0.5	0	0.5	1	0		1	1	1	1	1	1	1	1	[illegible]
113	1	0	0	0.5	1	1	0		0	0.5	1	1	1	1	1	1	[illegible]
114	1	0	1	0	0	1	1	1	1	1	1	1	1		1	1	[illegible]
115	1	0	0	0	0	1	0	1	1	0.5	0	1	1	0.5	1	0	[illegible]
116	1	0	0.5	0	1	1	1	1	0.5	0.5	0	1	1	1	1	1	[illegible]
117	1	1	0.5	1	1	1	1	1	1	1	1	1	0.5		1	0	[illegible]
118	1	1	0.5	1	1	1	1	1	1	1	1	1	1	1	1	1	[illegible]
119	1	0.5	0	0.5	1	1	0	1	1	1	1	1	0.5		1	1	[illegible]
120	1	0	0.5	1	0	1	1	1	1	0.5	1	0.5	1	1	1	1	[illegible]
121	1	0.5	0	0	0	1	0.5	1	1	1	0.5	0.5	1	1	1	1	[illegible]
122	1	1	0.5	0	1	1	0.5	1	1	1	1	1	1	1	1	1	[illegible]
123	0	1	0	0	0	1	0	1	0	0	1	1	0.5		1	1	[illegible]
124	0.5	0	0		0	1	0.5		1	0		0.5	1	1	1	1	[illegible]
125	1	1	0.5	1	0	1	1		1	1	1	1	1	1	1	1	[illegible]
126	1	1	0	0	0	1	0.5	1	1		1	0.5	0.5		1	1	[illegible]
127	1	0	0.5	0	0.5	1	1	1	1	1	1	1	0.5	1	1	1	[illegible]
128	1	0	0		1	1	0	1	1	1	1		0	1	1	1	[illegible]
129	1	0	1	0.5	1	1	0.5	1	1	1	1	0.5	1	1	1	1	[illegible]
130	1	0	1	0	1	1	0	1	0	0.5	0.5	1	0	1	1	0	[illegible]
131	1	1	0.5	1	1	1	0.5	1	1	1	1	1	1		1	1	[illegible]
132	1	0	0	1	0.5	1	0.5		1	1	0.5	1	0.5		1	1	[illegible]
133	1	0	0		0.5	1	0.5		1			1	1		1	0	[illegible]
134	1	0	1	0	1	1	0	1	0	1	0.5	1	0.5	1	1	0	[illegible]
135	1	0	0	0	1	1	0		1	1	1	1	1	1	1	1	[illegible]
136	1	1	1	1	0.5	1	1	1	1		1	1	0.5		1	0	[illegible]
137	1	0	1	1	0.5	1	0.5	1	1	0	1	0.5	1		1	1	[illegible]
138	1	1	0	1	1	1	1	1	1	1	1	1	1		1	1	[illegible]
139	1	0	0.5	0	1	1	0.5	1	1	0.5	1	1	0.5	1	1	1	[illegible]
140	0.5	0	0.5	1	0	1	0	1	1	0.5	0.5	1	1	0	1	1	[illegible]
141	0.5	0	0	0	0	0	0.5		1	0.5	0	1	0	0	0.5	0	[illegible]
142	1	0	0.5	1	1	1	0	1	0	0	0.5	1	0		1	1	[illegible]
143	1	0	0	1	0.5	1	0.5	1	1	0.5	0.5	1	1	0	1	1	[illegible]
144	1	0	0.5	1	1	1	0.5		1	1	1	1	1	0	1	1	[illegible]
145	1	0	0	1	1	1	1		1	0.5	1	0.5	0	1	1	1	[illegible]
146	1	1	0	1	0	1	0	0.5	1	0.5	1		0.5	1	1	0	[illegible]
147	1	0	0	1	1	1	1	1	1	0.5	1	1	0.5	1	1	1	[illegible]
148	1	1	0	0	0	1	1	1	1	1	1	1	0.5	0	1	1	[illegible]
149	1	1	1	1	1	1	1	1	1	1	1	1	1	1	1	1	[illegible]
150	1	0	0.5	1	0.5	1	0.5	1	1	0.5	0.5	1	1	0	1	1	[illegible]
151	1	0	0.5		0.5	1	0.5		1	0.5	0.5	1	0.5	0	1	1	[illegible]
152	1	0	0.5	1	0.5	1	1	1	1	1	1	1	1	1	1	1	[illegible]
153	1	0	1		1	1	1		1			1	1	0	0.5	1	[illegible]
154	1	0	0.5	0	0	1	1	1	1	1	0	1	0.5	0	1	0	[illegible]
155	1	1	0.5	1	0	1	1	1	1	1	1	1	1	0	1	1	[illegible]
156	1	0	0	0	0	1	0		1	0.5	1	1	1	0	0.5	1	[illegible]
157	0.5	0	0.5		0.5	0	0.5			0.5		1	1		1	0.5	[illegible]
158	1	1	1	1	1	1	1	1	1	0.5	1	1	1	1	1	1	[illegible]
159	0.5	0	0	0	0	1	0.5			0	0.5	1	1		1	0	[illegible]
160	1	0	0	0	0	1	0.5	1	0	1	0	1	0	1	1	0	[illegible]
161	1	0	1	0	0	1	0.5	1	1	1	0	1	1		1	0	[illegible]
162	1	0	0.5	1	0	1	1		1	1	1	1	0	1	1	1	[illegible]
163	1	0	0	0	0	1	0			0.5	0	1	0		0.5	0	[illegible]
164	1	1	0	0	0	1	1	1	1		1	1	1	1	1	1	[illegible]
165	1	1	0.5	1	0.5	0	1	1	1	0.5	1	1	0.5		1	0	[illegible]
166	1	0	0	0	1	1	1		1	0.5	0.5	1	1	1	1	1	[illegible]
167	1	0	0	1	0	1	1	1	1	0	0.5	1	0		0.5	0	[illegible]
168	1	0	0	0	0	0	0.5		1		1	1	1	1	1	0	[illegible]
169	1	0	1	1	0		0.5				1	1	1		1		[illegible]
170	1	0.5	1	1	0	1	0		1		1	1	1		1	0	[illegible]
171	1	0	0.5	1	0	1	1		0	0	0.5		0.5		1	0	[illegible]
172	1	0	0	0	1	1	0		1	1	1	1	1		1	0	[illegible]
173	1	0	0.5	1	1	1	1	1	0	0.5	0.5	1	1	1	1	0	[illegible]
174	1	0	0.5	1	0.5	1	0				1	1	1	0	1		[illegible]
175	1	0		0	1	1	0.5			0.5	0	1	1		1	0	[illegible]
176	1	0	0.5	0.5	1	1	0			1	1	1	0.5		0.5	0	[illegible]
177	1	0	0.5	1	1	1	0.5				1	1	0.5	1	0.5	0	[illegible]
178	1	0	0.5	0.5	1	1	0.5		1	1	1	1	1	1	1	1	[illegible]
179	1	0	0.5	0.5	0.5	1	0		1	1	1	1	1	1	1	1	[illegible]
180	1	0	0.5	0	1	1	0			0.5	1	1	1	1	1	1	[illegible]
181	1	0	0.5	1	0.5	1	0.5			0.5	1	1	0.5		1	1	[illegible]
182	1	0	0.5	1	1	1	1			1	0	1	1		1	0	[illegible]
183	1	0	0.5		0.5	1	0.5			1		1	1		1		[illegible]
184	1	0	0.5		0	1	0			0		1	0	1	0	0	[illegible]
185	1	0.5	0	1	1	1	0.5	1	1	1	1	1	1	1	1	0	[illegible]
186	1	0	0.5	0	0.5	1	0.5			0	1	1	1	1	1	1	[illegible]
187	0.5	0	0		0	1	0.5			0.5	1	1	1		0.5		[illegible]
188	0.5	0	0.5		0	1	0			0		1	0.5		0.5	0	[illegible]
189	1	0	0.5		0	1	0.5			0.5		1	1		0.5	1	[illegible]
190	1	0	0.5	1	1	1	1			0	1	1	0.5	1		0	[illegible]
191	1	0	0		0	1	0.5			0.5		1	1			0	[illegible]
192	1	0	0.5	1	0	1	1	1	1	0.5	1	1	0.5		1	1	[illegible]
193	1	0	0.5	1	0	1	0.5			0	1	1	1		0.5	1	[illegible]
194	1	0	1	1	0.5	1	0.5		1	0.5	1	1	1		1	1	[illegible]

Research Questions	1	2	3	4	5	6	7	8	9	10	11	12	13	14	15	16	17	18	19	20	21	22	23	24	25	26–91
195	1	0	0.5	1	1	1	1		1	0	1	1	0		1	0		0.5		0	0.5	0				[illegible]
196	1	0	0.5	1	0	1	0.5	1		0	1	1	1		1	0			0.5	0	0	1	0.5			[illegible]
197	1	0	0.5	1	0	1	0.5			0.5	0.5	0.5	0.5	1	0.5	0	1		0.5	0	0	0			0.5	[illegible]
198	1	0	0.5	1	0	1	0.5	1	1	1	1	1	0.5		1	0	1	0.5	0.5	0	0	1				[illegible]
199	1	0	0	1	0.5	1	0.5			1	1	1	0.5	1	1	0	1	0.5	0.5	0	0	0	0.5		1	[illegible]
200	1	0	0	1	1	1	0			0	1	1	0		1	0			0.5	0	0	0				[illegible]
201	1	0	0.5	1	0.5	1	0.5			1	1	1	0.5		1	1		0.5	0.5	0	0	0				[illegible]
202	1	0	0	1	1	1	0			1	1	0.5	1	1	1	1		0.5	1	0	0	0	0.5		1	[illegible]
203	1	0	0	1	0	1	0			0	0	0.5	0	1	1	0			1	0	0	0			1	[illegible]
204	1	0.5	1	1	0	1	0.5			1	1	1	1	1	1	1	1	0.5	1	1	0	1	0.5		1	[illegible]
205	1	0	1	0.5	0.5	1	0.5		1	1	1	1	1	1	1	1	1	0.5	1	1	0	0				[illegible]

CRITERION 3: DELIVERABILITY AND ACCEPTABILITY **Taking into account: (i) the infrastructure and resources required to deliver the resulting intervention(s) (e.g. human, financial and logistic resources, government capacity and partnerships), and (ii) the need for change in demand, beliefs and attitudes of users to ensure broad acceptability (s), Would you say that the proposed research would lead to deliverable interventions, or improve their deliverability?**

Research Questions	1	2	3	4	5	6	7	8	9	10	11	12	13	14	15	16	17	18	19	20	21	22	23	24	25	26–91
1	1	1	0.5	1	1	1	1	1	1		0.5	1	0.5	0	1	0	0		1	1	0.5	1	0	0		[illegible]
2	1	0	0	1	0	1	1		1	0.5	1	1	0.5	0	1	0	0		1	1	0.5	1	0.5	1	1	[illegible]
3	1	0	0.5	0.5	1	1	1	1	1	1	0	1	0.5	1	1	0.5	1		1	0			0.5	0	0.5	[illegible]
4	1	0	0.5	1	1	1	1	1	1	0.5	0.5	1	1	0	1	0	1		1	1	0	1	1	1	1	[illegible]
5	1	0	0.5	1	0.5	1	1		1	1	1	1	1	0	1	1	0.5		1	1	0.5	1	1		0	[illegible]
6	1	0	0.5	1	0	1	0.5		1		1	1	0	0	1	1	1	1	1	0	0.5	1	1	1	1	[illegible]
7	1	1	1	1	1	1	1	1	1	1	1	1	1	0	1	1	1	0	1		1	1	0		0	[illegible]
8	1	0	1	1	1	1	1	1	1	1	1	1	0	0	1	1	1	1	1	1	0.5	1	1	1	1	[illegible]
9	1	0.5	0.5	1	1	1	1	1	1	1	1	1	0.5	1	1	1	1	0	1	1	0.5	1	1		0	[illegible]
10	1	0	0.5	1	1	1	1	1	1	0.5	1	1	1	0	1		1	1	1	0	0.5	1	1	1	1	[illegible]
11–144	[illegible]																									

Research Questions	Scores (columns 1–91)
145	[illegible]
146	[illegible]
147	[illegible]
148	[illegible]
149	[illegible]
150	[illegible]
151	[illegible]
152	[illegible]
153	[illegible]
154	[illegible]
155	[illegible]
156	[illegible]
157	[illegible]
158	[illegible]
159	[illegible]
160	[illegible]
161	[illegible]
162	[illegible]
163	[illegible]
164	[illegible]
165	[illegible]
166	[illegible]
167	[illegible]
168	[illegible]
169	[illegible]
170	[illegible]
171	[illegible]
172	[illegible]
173	[illegible]
174	[illegible]
175	[illegible]
176	[illegible]
177	[illegible]
178	[illegible]
179	[illegible]
180	[illegible]
181	[illegible]
182	[illegible]
183	[illegible]
184	[illegible]
185	[illegible]
186	[illegible]
187	[illegible]
188	[illegible]
189	[illegible]
190	[illegible]
191	[illegible]
192	[illegible]
193	[illegible]
194	[illegible]
195	[illegible]
196	[illegible]
197	[illegible]
198	[illegible]
199	[illegible]
200	[illegible]
201	[illegible]
202	[illegible]
203	[illegible]
204	[illegible]
205	[illegible]

CRITERION 4a: POTENTIAL IMPACT Taking into account: (i) the best available information on the burden of neonatal mortality, stillbirths, and severe long-term disability and its causes; (ii) efficacy, deliverability and acceptability of health interventions affected by the proposed research; Would you say that reaching the endpoints of the proposed research has a capacity to directly and indirectly remove at least 5% of overall global burden?

Research Questions	1–91
1	[illegible]
2	[illegible]
3	[illegible]
4	[illegible]
5	[illegible]
6	[illegible]
7	[illegible]
8	[illegible]
9	[illegible]
10	[illegible]
11	[illegible]
12	[illegible]
13	[illegible]
14	[illegible]
15	[illegible]
16	[illegible]
17	[illegible]
18	[illegible]
19	[illegible]
20	[illegible]
21	[illegible]
22	[illegible]
23	[illegible]
24	[illegible]
25	[illegible]
26	[illegible]
27	[illegible]
28	[illegible]
29	[illegible]
30	[illegible]
31	[illegible]
32	[illegible]
33	[illegible]
34	[illegible]
35	[illegible]
36	[illegible]
37	[illegible]
38	[illegible]
39	[illegible]
40	[illegible]
41	[illegible]
42	[illegible]
43	[illegible]
44	[illegible]
45	[illegible]
46	[illegible]
47	[illegible]
48	[illegible]
49	[illegible]
50	[illegible]
51	[illegible]
52	[illegible]
53	[illegible]
54	[illegible]
55	[illegible]
56	[illegible]
57	[illegible]
58	[illegible]
59	[illegible]
60	[illegible]
61	[illegible]
62	[illegible]
63	[illegible]
64	[illegible]
65	[illegible]
66	[illegible]
67	[illegible]
68	[illegible]
69	[illegible]
70	[illegible]
71	[illegible]
72	[illegible]
73	[illegible]
74	[illegible]
75	[illegible]
76	[illegible]
77	[illegible]
78	[illegible]
79	[illegible]
80	[illegible]
81	[illegible]
82	[illegible]
83	[illegible]
84	[illegible]
85	[illegible]
86	[illegible]
87	[illegible]
88	[illegible]
89	[illegible]
90	[illegible]
91	[illegible]
92	[illegible]
93	[illegible]
94	[illegible]

	1	2	3	4	5	6	7	8	9	10	11	12	13	14	15	16	17	18	19	20	21	22	23
197	0.5	0	0.5	1	0	1	0			0	0	0	0	0	1	1	1		0	0	0	0	
198	1	0	0	1	0	1	0			0	0	1	0	0	1	0	1	0.5	0.5	0	0	1	
199	1	0	0	1	0	0	0				0	1	0	0	1	0	1	0.5	0.5	0	0	0	1
200	0.5	0	0	1	0.5	1	0				0	1	0	0	1	0			0.5	0	0	0	
201	1	0	0	1	0	1	0			0	0	1	0	0	1	0		0.5	1	0	0	0	
202	0.5	0	0	1	1	1	0			0	0	0.5	0	0		1		0.5	0.5	0	0	0	0.5
203	0.5	0	0	1	0	1	0			0	0	0.5	0	0	1	0			1	0	0	0	
204	1	0	0.5	1	0	1	0			0	0.5	0	0	1	1	0	1	0.5	1	1	0	1	1
205	0.5	0	0	0.5	0.5	1	0			0	0.5	1	0	0	1	1	1	0.5	1	0.5	0	0	

CRITERION 4b: POTENTIAL IMPACT Taking into account: (i) the best available information on the burden of neonatal mortality, stillbirths, and severe long-term disability and its causes; (ii) efficacy, deliverability and acceptability of health interventions affected by the proposed research; Would you say that reaching the endpoints of the proposed research has a capacity to directly and indirectly remove at least 10% of overall global burden?

Research Questions	1	2	3	4	5	6	7	8	9	10	11	12	13	14	15	16	17	18	19	20	21	22	23
1	0	0	0	0	0	0	0	0	0	0	0	1	0	0	0	0	0		0	0	0	1	0
2	0	0	0	0	0	0	0		0	0	0	1	0	0	0	0	0		0	0	0	1	0
3	0	0	0	0	0	0	0	0	0	0	0	0	0	0	0	0	0		0	0			0
4	0	0	0	0	0	0	0	0	0	0	0	1	0	0	0	0	0		0	0	0	1	1
5	1	0	0.5	0	0.5	1	0		1	0.5	1	1	0	0	1		1	1	0.5	1	1	1	1
6	1	0	0	0	0	0	0		0		0	1	0	0	1	1	1	0.5	0.5		0.5	1	1
7	1	0	0	0	0.5	0	0	0	1	0	1	0	0	0	1	1	1	0	0.5	0	0.5	1	0
8	1	0	0	0	0	0	0	0	0	1	1	1	0	0	1	1	1	0.5	0.5	0	0.5	1	1
9	1	0	0	0	0	1	0	0.5	1	1	1	0.5	0	0	1	1	0.5	0	0.5	1	0	1	1
10	1	0	0.5	0	0	1	0	0	0	0	0	1	1	0	0.5		1	1	0.5	0	0	1	1
11	1	0	0.5	0	0.5	0	0	0.5	1	1	1	1	0	0	1	1	1		0.5	0	0	1	1
12	1	0	0	0	0.5	0	0	0.5	1	1	1		0	1	1	1	1		0.5	0	0.5	1	1
13	1	0	0.5	0	0.5	0	0		1	0.5	0.5	1	0	0	1	1	1	0.5	0	0	0	1	0
14	0	0	0.5	0	0.5	1	0		0	0	0.5	1	0	0	1	0	1	1	0	0	0.5	1	1
15	1	0	0	0	0.5	1	0	0	1	0.5	1	1	0	1	1	1	0.5	0.5	0.5	1	0.5	1	1
16	0	0	0		0.5	0	0		0	0	0	1	0	0		0	0.5	0	0			1	0
17	1	0	0.5	0	0.5	0	0	0	0	0	0	1	0	0	1	0.5	1	0.5	1	0	0	1	1
18	1	0	0	0	0	1	0	0	1		1	1	0	1	1	0	1		1	0	0	1	
19	0	0	0	0	0	1	0		1	0	0	1	0	0	1	0	1	1	0.5	0	0	1	1
20	0	0	0	0	1	0	0.5	0.5	1	0	0	1	0	0	0	0	0.5		0	0	0	1	0

45	1	0	0.5	0	0	1	1		1	0	0	1	0	0	1	0	1		0.5	0		1	1	1	0		0	
46	1	1	0	0	0.5	1	0	0.5	1	0	1	1	0	0	1	0	1	0.5	0	0	0	1	1	0	0.5	1		0
47	1	0	0	0	0	1	0	0.5	1	0	1	1	0	0	1	0	1		0		1	1	1		0.5	1	0	0
48	1	0	0	0	0	0	0	0	1	0	0	0	0	0	0	0	1	0.5	0	0	0.5	1	0			0.5	0	0
49	1	1	0	0	0	0	0	0.5	1	0	0	1	0	0	0	0	1		0.5	1	0.5	1	1		1		0	0
50	1	0	0	0	0	0	0	0.5	1	0.5	0.5	1	0	0	1	0	1	0.5	0.5	0	0		1		0.5		0.5	0
51	0.5	0	0	0	0	0	0	0.5	0		0.5	1	0	0	1	0	1		0.5	0	0		1	0	0		0.5	0
52	1	1	0	0	0	1	0		1	0.5	0.5	1	0	0	1	0	1		0.5	0	0.5	1	1		0.5		0	0
53	1	0	0		0	1	0	0	1	0.5	1	1	0	0	0	0	1	0.5	0.5	0.5			1	0	0.5			0
54	1	1	0	0	0	0	0	0	1	0	1	0.5	0	0	0	0	1		0.5	0	0.5	1	0	0	0	1	0.5	0
55	1	0	0	0	0	0	0	0		0.5	1	0	0	0	0	0	0.5	0.5	0	0	0.5	1	1	0	0.5			0
56	1	0	0	0	0	0	0	0		0	0	0	0	0	0	0		0.5	0.5	0	1		1	0	0			0
57	0	1	0	0	0	0	0	0	1		0	0.5	0	0	0	0	0.5		0.5	0	0	0.5	1					0
58	1	0	0.5	0	0	1	0	0	1	0.5	1	1	0	0	0	0	1	0.5	0	0	0	1	0	0	0.5	1	0	0
59	1	0	0.5	0	0	0	0	0	1	0	0	0	0	0	0	0	1	0.5	0.5	0	0		0.5					0
60	1	0	0	0	0	0	1	0	1	0.5	1	1	0	0	0	0	1		0	0	0	1	0	0	0.5		0	0
61	0	0	0	0	0.5	0	0	0.5	0	0	0	1	0	1	1	0	0.5	0.5	0	0	0	1	0		1	1		0
62	1	0	0	0	0	1	0	0	0	0	0.5	1	0	0	1	0	1	0.5	0	0	0	1	1	0	0.5			0
63	1	0	0	0	0	0	0		1	1	1	0	0	0	1	0	1		0	0			0	0	1		0.5	0
64	0	0	0	0	0	0	1		1	0	0.5	1	0	0	1	0	1		0	0	1		1	0			0.5	0
65	0.5	0	0	0	0	0	0		1	0	0.5	0.5	0	0	1	0	0.5	0.5	0	0	0	1	0		1	1	0	0
66	1	0	0	0	0.5	0	0	0	1	0	0	1	0	0	1	0	1	0.5	0	0	0.5		1	0	1		0	0
67	1	1	0.5	0	0	0	0	0.5	0	0	1	1	0	1	1	0	1		0.5	0	1	1	1	0	1	1	0.5	0
68	0.5	0	0	0	0	0	0	0.5	1	0	0	1	0	0	1	1	1		0	0	0.5	1	0	0	1			0
69	0	0	0	0	0	0	0		1	0	0.5	1	0	0	0	0	0.5		0	0	0	1	0.5	0	0.5			0
70	1	0	0.5	0	0.5	0	0	0	1	0	0.5	1	0	0	1	0	0.5	0.5	0	1	0.5	1	1			1	0.5	0
71		0	0	0	0	0	0	0	1	0	0.5	1	0	0	1	0	1	0.5	0	0	0.5	1	1		1		0.5	0
72	1	1	0	0	0	0	0	0.5	1		0	1	0	0	1	0	1	0.5	0	0	0.5		0		0.5		0	0
73	1	0	0	0	0	0	0		1	0	0.5	1	0	0	1	0	1	0.5	0	0	1	1	0.5	0			0.5	0
74	1	0	0.5	0	0	0	0		1	0.5	1	1	0	1	1	0	1	0.5	1	0	1	1	0		0.5		0.5	0.5
75	1	0.5	0.5	0	0	0	0		0	1	1	0.5	0	1	1	0	1	1	0.5	0	0.5	1	1	0	0.5		0.5	0
76	1	0.5	0	0	0	0	1	0.5	1	0	1	1	0	0	1	0	1	0.5	0.5	0	0.5	1	1		1		1	0
77	1	0	0	0	0.5	0	0	1	0	0	0.5	1	0	1	1	0	1	0.5	0.5	0	0	1	1			1	1	0
78	1	1	0	0	0	1	0	0.5	1	1	1	1	0	0	1	0	1	0.5	0	0	0.5	1	1		0.5		1	0
79	1	0	1	0	1	0	0	0	1	1	0	1	0	0	1	1	1	1	0	0	0.5	1	1		0		0.5	0
80	0	0	0.5	0	0	0	0		0	0	0	1	0	0	0	0	1	0.5	0	0	0.5	1	0.5				0	0
81	0	1	0	0	0	1	0	0.5	1	0	0	1	0	0	0	0	1	0.5	0	0	0.5	1	0.5		0.5		0.5	0
82	1	0	0	0	0	0	0	0	1	0	0	1	0	0	1	0	1	0.5	0.5	0	1	0	1		0.5		0.5	0
83	1	1	0	0	0	1	0	0.5	1	0	0.5	1	0	0	1	0	1		0	0	0.5	0.5	1		0		0.5	0.5
84	0	0	0	0	1	0	0		0	0	0	1	0	0	0	0	1	0.5	0	0	0	1	0.5		0		0.5	0
85	1	0	0	0	1	0	0	0	0	0	0	1	0	0	1	0	1		0	0	0	1	q				0.5	0
86	1	1	0	0	1	1	0	0	1	0	1	1	0	0	1	0	1	0.5	0	0	0.5	1	1		0.5		0.5	0
87	0	0	0	0	0	0	0		0	0	0	0	0	0	0	0	0.5		0	0	0	1	0				0.5	0
88	0	0	0.5	0	0	0	0	0	0	1	0	0.5	0	0	0	1	1		0.5	0	0.05	0	0.5		1		0.5	0
89	1	0	0	0	0	0	0		0	0	0	1	0	0	0	0	1		0	0	0	1	0.5	0	0.5		1	0
90	1	0	0	0	0	0	0		0	0	0	1	0	0	0	0	1	1	0	0	0	1	0.5				0.5	
91	0	0	0	0	0	0	0			0	0	1	0	0	0				0	0	0	0	0				0	0
92	0	0	0	0	0	0	0			0	0	1	0	0	0	0	0.5		0	0	0	1	0.5				0	0
93	0	0	0	0	0	0	0	0		0	0	1	0	0	0	0	0.5		0	0	0	0	0		0		0.5	0
94	0	0	0	0	0	0	0			0	0	1	0	0	0	0	0.5		0	0	0	0	0.5				0	0
95	1	0	0	0	0	0	0			0	0	1	0	1	0	0	1	0.5	0	0	0	0	1		1		0.5	0
96	1	0	0.5	0	0	1	0		1	0	0	1	0	0	1	0	1		0	0	0	0.5	0.5	0			1	0
97	1	0	0.5	0	0.5	1	0		1	1	0	0	0	0	0	0	1		0	0	0	1	1	0		1	0.5	0
98	1	0	0	0	0	1	0			0	1	0.5	0	0	0	0	1	0.5	0	0	0	0	0.5				0.5	0
99	0	0	0	0	0.5	0	0			0		0	0	0	0	0	1	0.5	0	0	0.5	1	0.5		0.5		0.5	
100	0.5	0	0	0	0.5	0	0		0	0		0.5	0	1	0	0		0.5	0	0	0	1	0.5		0.5		0.5	0

CRITERION 5: EFFECT ON EQUITY: Taking into account: (i) the present distribution of the burden of neonatal mortality, stillbirths and severe long-term disability by equity strata within the target population; (ii) the evidence of the long-term effects on equity of the existing health interventions; Would you say that the proposed research would be likely to improve equity in burden distribution by 2025?

Research Questions	1–91
1	[illegible]
2	[illegible]
3	[illegible]
4	[illegible]
5	[illegible]
6	[illegible]
7	[illegible]
8	[illegible]
9	[illegible]
10	[illegible]
11	[illegible]
12	[illegible]
13	[illegible]
14	[illegible]
15	[illegible]
16	[illegible]
17	[illegible]
18	[illegible]
19	[illegible]
20	[illegible]
21	[illegible]
22	[illegible]
23	[illegible]
24	[illegible]
25	[illegible]
26	[illegible]
27	[illegible]
28	[illegible]
29	[illegible]
30	[illegible]
31	[illegible]
32	[illegible]
33	[illegible]
34	[illegible]
35	[illegible]
36	[illegible]
37	[illegible]
38	[illegible]
39	[illegible]
40	[illegible]
41	[illegible]
42	[illegible]
43	[illegible]
44	[illegible]
45	[illegible]
46	[illegible]
47	[illegible]
48	[illegible]
49	[illegible]
50	[illegible]
51	[illegible]
52	[illegible]
53	[illegible]
54	[illegible]
55	[illegible]
56	[illegible]
57	[illegible]
58	[illegible]
59	[illegible]
60	[illegible]
61	[illegible]
62	[illegible]
63	[illegible]
64	[illegible]
65	[illegible]
66	[illegible]
67	[illegible]
68	[illegible]
69	[illegible]
70	[illegible]
71	[illegible]
72	[illegible]
73	[illegible]
74	[illegible]
75	[illegible]
76	[illegible]
77	[illegible]
78	[illegible]
79	[illegible]
80	[illegible]
81	[illegible]
82	[illegible]
83	[illegible]
84	[illegible]
85	[illegible]
86	[illegible]
87	[illegible]
88	[illegible]
89	[illegible]
90	[illegible]
91	[illegible]
92	[illegible]
93	[illegible]
94	[illegible]
95	[illegible]
96	[illegible]
97	[illegible]
98	[illegible]
99	[illegible]
100	[illegible]
101	[illegible]
102	[illegible]
103	[illegible]
104	[illegible]
105	[illegible]
106	[illegible]
107	[illegible]
108	[illegible]
109	[illegible]
110	[illegible]
111	[illegible]
112	[illegible]
113	[illegible]
114	[illegible]
115	[illegible]
116	[illegible]
117	[illegible]
118	[illegible]
119	[illegible]
120	[illegible]
121	[illegible]
122	[illegible]
123	[illegible]
124	[illegible]
125	[illegible]
126	[illegible]
127	[illegible]
128	[illegible]
129	[illegible]
130	[illegible]
131	[illegible]
132	[illegible]
133	[illegible]
134	[illegible]
135	[illegible]
136	[illegible]
137	[illegible]
138	[illegible]
139	[illegible]
140	[illegible]
141	[illegible]
142	[illegible]
143	[illegible]
144	[illegible]
145	[illegible]
146	[illegible]
147	[illegible]
148	[illegible]
149	[illegible]
150	[illegible]
151	[illegible]
152	[illegible]
153	[illegible]
154	[illegible]

155	0.5	1	0.5	1	0	1	0	1	1	0	1	1	1	0	1	1	1		1	1	0	1	0.5
156	0	0	0	0	0.5	1	0		0	0	1	1	1	0	0	1	1	0.5	0.5	1	0	0	0.5
157	0	0	0		0.5	1	0		0.5	0		1	1	0	0	1		0.5	0.5	0		0	0.5
158	0	0	1	1	1	1	0	1	1	0	1	1	1	1	0	1	1	0.5	0.5	0	0	1	1
159	0.5	0	0	0	0	1	0		0	0	1	1	1	0	0		1		1	1	0	1	1
160	1	0	0	0	1	1	1			0	1	1	0.5	1	0	0	0.5		0.5		0	0	0.5
161	0.5	1	1	0	0	1	1	1	1	0	1	1	1	0	0	0	1	1	0.5		0.5	0	0.5
162	0.5	0	0.5	1	0	1	0			0.5	1	1	0.5		0	1	1	0.5	0.5		0	0	0.5
163	1	0	0	0	0	0	0			0	1	1	1		0	0	0	0.5	0.5		0	0	0.5
164	0.5	1	0	0	0.5	1	0	1	1	0.5	1	1	0.5	1	1	1	1	0.5	1	1	0.5	1	1
165	0.5	0	0	1	0	1	1	1	1	0.5	1	1	1	0	1	0	1		1	0	0.5	0	1
166	0.5	0	0	1	1	1	1		0	0	1	1	1		0	1	1		0.5	0	0	0	0.5
167	1	0	0	1	0	1	1	0.5	0	1	1	1	1	0	0	0	1		0.5	0	0	0	0.5
168	0.5	0	0	0	0.5	0	0		1	0	1	1	1		0	0	0.5		0.5	0		1	1
169	0.5	0	1	1	0		0			0	1	1	0.5		0		0.5		0.5	0	0	0	0.5
170	0.5	0	1	1	0	1	0		1	0	1	1	0.5	0	0	0	1		0.5	0	0	1	1
171	0.5	0	0	1	0	1	0		0		0.5	1	0.5	0	0	0	1		0.5	0	0	1	0.5
172	1	1	0	0	0	1	0		1	0.5	1	1	1		0	0	0.5		0.5	0	0.5	1	1
173	1	0	0.5	1	0	1	1	1	0	0.5	1	1	0.5	1	0	0	0.5	0.5	0.5	0	0.5	0	0
174	0.5	0	0	1	0	1	0			0.5	0.5	1	0.5		0				0.5	0	0.5	0	0.5
175	0.5	0		0	0	1	0			0	0	1	1		0	0			0.5	0	0	1	0.5
176	0.5	0	0	0.5	0	1	0			0	0.5	1	0.5	0	0	0			0.5	0	0		1
177	0.5	0	0.5	1	0	1	0			0	1	1	0.5	1	0	0			0.5	0	0		1
178	1	0	0.5	1	1	1	0		0	0	1	1	0.5	1	0	1	0.5		0.5	1	0	0	
179	0.5	0	0	0.5	1	1	0		1	0	1	1	0	1	0	1	0.5		0.5	0	0		0.5
180	0.5	0	0	0	0	1	0			0	1	1	0	1	0	1			0.5	0	0		
181	0.5	0	0	0	0.5	1	0			0	1	1	0	0	0	0			0.5	0	0		
182	0.5	0	0	1	0	1	1		0	1	1	1	1	0	1	0	1		1	0	1		
183	0.5	0	0		0	1	0			0	1	1	0.5	0	0				0.5	0	0		
184	1	0	0		0	1	0			0	1	1	0.5	1	0	0			0.5	0	0	1	
185	0.5	0	0	1	1	1	0	0.5		0	1	1	0.5	1	0	1	1		0.5	1	0	1	0.5
186	0.5	0	0.5	1	0	1	0			0	0.5	1	0.5	0	0	1	1		0.5	0	0	0	
187	0.5	0	0		0	1	0			0		1	1	0	0					0	0	1	
188	0.5	0	0		0	1	0			0		1	0.5	0	0	0		0.5	0	0	0	
189	1	0	0.5		0.5	1	0			0		1	0.5	0	0	1		0.5	0	0	0	1
190	0	0	0	1	0	1	0			0		1	0	0	0	0			0.5	0	0	0	
191	1	0	0		0	1	0			0		1	0	0	0	0			0.5	0	0	0	
192	0.5	0	0.5	1	0	1	0	1		0	1	1	1	0	0	1			0.5	0	0	0	
193	1	0	0.5	1	1	1	0			0	1	1	0.5	0	0	1			0.5	0	0	1	
194	1	0	1	1	1	1	0			0	1	1	0.5	0	0	1	1		0.5	1	0	1	
195	0.5	0	0	1	0.5	1	0			0	0	1	0	0	0	0				0	1	0	
196	0.5	0	0	1	0	1	0	1		0	0.5	1	0	0	0	0			0.5	0	0	1	0.5
197	0.5	0	0	1	0	1	0			0	0.5	1	0	0	0	0	1		0	0	0	0	
198	0.5	0	0	1	0	1	0	0.5		0	1	1	0.5	0	0	0	1		0.5	0	0	1	
199	0.5	0	0	1	0	1	0			0.5	0	1	0	0	1	0	1		0.5	0	0	0	1
200	0.5	0	0	1	0.5	1	0			0	0	1	0	0	1	0			0.5	0	0	0	
201	1	0	0	1	0	1	0			0	1	1	0	0	0	1			1	0	0	0	
202	1	0	0	1	0	1	0			0	1	1	0.5	0	0	1		0.5	0.5	0	0	0	0.5
203	1	0	0	1	0	1	0			1	1	1	1	1	1	0			1	0	0	0	
204	0.5	0	0.5	1	0	1	0			0.5	1	1	0.5	1	1	1	1	0.5	0.5	1	0	1	1
205	0.5	0	0	1	0	1	0			0	1	1	0.5	0	0	1	1	0.5	0.5	0	0	0	1

155		1	1	1	0.5	0	0		1		0	1	0.5	0	1	0.5	0.5	1		1	1	0	1	1
156		1	1	1	0.5	0	0		1		0	0.5	1	0	1	0.5	0			1	0.5	0	1	1
157		1	1	1					1		0	0	1	1	0.5	0.5	0				0.5	0		1
158		0	1	1	0	1				1		0	0	1	0	1	0.5	1				0.5	0		1
159		0.5	1	1	0.5	0	0	1	1		0	1	1	1	1	1	0			0.5	0.5	0		1
160		1	1	0		0	0		1		0	0	1	1	1	0.5	0			0.5	0.5	1		1
161		1	1	0	1	1	1	1	0.5		0	0	1	0	1	0.5	0.5			0.5	0.5	0	1	1
162		1	1	1	0.5				1		0	1	1	0	1		0			0.5	0.5	1		1
163			0	0		0	0		1		0.5	1	0.5	0	1		0			0.5	0.5	0		1
164		0.5	1	1	0.5	0	0		1		0	1	1	0	1	0.5	1			0.5	1	1	1	1
165		1	1	0.5	0.5			1	1		0.5	0	1	0	1	0.5	0					1	1	1
166		0.5	1	1	0	1	1		1		0	1	1	0	1	0.5	0.5				1	0.5		1
167			1	0		1	1	0	0.5		0.5	1	1	1	1	0.5	0	0		0.5		1		1
168		1	1	0.5	0.5	0.5			1		0	0	1	1	1	0.5	0					0		1
169			1	1	0.5	0.5			1		0	0.5			1	0	0			0	1	0		1
170			1	0.5	0.5	0.5			0.5		0	0			0	0	0			0		1		1
171			1	1		0	0		0.5		0	0	0.5		1	0	0				0.5	0		1
172		1	1	0.5	0.5	0.5	0		1		0	1	1	0	1	0.5	0			0.5	0.5	0	1	1
173		1	1	0.5		0	0	0	1		0.5	1	0.5	0	1	1	0			0.5		1		1
174			0	1	0	0	0		0		0	0	0.5	0	1	0.5	0				0.5	0.5		1
175			1	0	0	0	0		1		0	0.5	0.5	0	1	0.5	0					0		1
176			1	0	0	0	0		1		0	0	0.5	0	1		0	0				0		1
177			0	0	0	0	0		1		0	0		0	1		0	0.5		0.5		0		1
178		1	0	0	0.5	1	1		1		0	1	0.5	1	1	0	0	0				0		1
179		0.5	0	1	0.5	1	1		1		0	1	0.5	0	1	1	0					0	1	1
180		0.5	0	0	0.5	1	1		1		0	0	0.5		1	0.5	0			0.5		0		1
181			1	0	0.5	0.5	0		1		0	0.5	0.5	0	1	0.5	0	0				0		1
182			1	1	0.5	0.5	0.5		0.5		0	1	1	0	0	0.5	0				0.5	0.5		1
183			1	1	0				1		0	0	0.5	0	1	0	0				0.5	0		1
184		1	1	0	0.5	0.5			1		0	1	0.5	0	1	0	0			0.5		0		1
185		0.5	1	0	0.5	0.5	0.5		1		0	1	1	1	1	0	0.5			1		0	0	1
186			0	0	0	0.5	0.5	0	1		0	0	0.5	0	1		0				1	0		1
187			0	1		0.5			1		0	0	0.5		0.5		0				0.5	0		1
188		0	0	0	0	0	0	0	1		0	0	0.5	0	1	0.5	0					0		1
189		0.5	1	1	0	0		0	1		0	0	0.5	0	1	0.5	0	0		0.5		0		1
190			1	0	0	0	0	0	1		0	0	0.5	0	1	0	0			0.5		0		1
191			0	0	0	0	0		1		0	0	0.5	0	1		0					0		1
192			0	0	0.5	1	1		1		0	1	1	0	1	0.5	0	0				0.5		1
193		0		0	0	0.5	0.5		1		0	1	0.5	0	1	0.5	0	1			1			1
194		1	1	0	0	0	0		1		0	1	0.5	1	1	1	0.5			0.5		0.5		1
195			1	0	0	0.5	0		1		0	1	0.5	0	1	1	0	0			0.5	0		1
196			1	0	0	0	0		1		0	0	0.5	0	1	0	0.5	0.5		0.5		0.5		1
197			1	1	0	0	0		1		0	0	0.5	0	1	0	0	0				0.5		1
198			1	0	0	0			1		0	1	1	0	1	0.5	0.5	0.5		1	1	0.5	0.5	1
199		1		0	0	0.5	0		1		0	1	1	0	1	0	0	0		0.5	1	1		1
200			1	0	0	0	0		1		0	1	0.5	0	1	0	0	0			1	0.5		1
201			0	0	0	0.5	0.5		0.5		0	0	0.5	0	1	0.5	0	0		nl	1	0.5	0	0
202		1	1	0	0	0.5	0		1		0	0	1	1	1	0.5	0	0.5			1	0.5		1
203		1	1	0	0	1	1	1	0.5		0.5	1	1	0	1	0.5	1	1				1	1	1
204		0.5	1	1	0	1			1		0	0	0.5	1	1	0.5	0.5				1	0		1
205			1	0	0				1		0	0.5	0.5	0	1	0.5	0					0		1

155	1	0	0	1			1	0			0.5		1	1	1	1	0.5	0	0.5	1		0	0.5
156	0.5	0		1			0	0			1	0.5	1	0.5	1	0	0.5	0	0	0.5		0	0
157		0		1			0	0			0	0.5	0.5	0.5	1	1			0		1	1	0
158	0	0	1	1	1	1	0	1			0.5	0.5	1	1	1	1	1	0	0.5	1		1	1
159	1	0	0	1			1	1	0		1	1	1	1	1	1	0	0	0	0	1	1	0.5
160	1	0	1	1			0	0	0		0	1	1	0	0.5	1	0	0	0	0	1	0	0.5
161	1	1	1	1			1	1	0.5		0.5	0.5	1	0.5	0.5	1		0	0	1	0	0	0.5
162	1	0	0	1			0	0			0	0.5	0.5	0.5	0.5	1		0	0	0.5		0	0
163		0	0	1			0	0			0	0	0.5	0.5	0	1		0	0.5		0	0	0
164	1	1	1	1			0	0	0.5		0.5	0.5	1	1	0.5	1	0.5	0	0.5	0	1	0	0
165	1	1		1			1	0	0		0	0.5	0.5	0	0.5	0	1	1	0.5	1	0	0	
166	1	0	1	1			1	0.5	0.5		1	0.5	0.5	0.5	0.5	1		0	0.5	1	1	1	0
167	0	0	1	1			0	1	0		1	0	1	0	0.5	1		0	0.5	0	0	0	0
168	0.5	1		1			1	0			0	1	0.5		0.5	1	0.5	0	0.5	0		0	0
169		0		1			0	0	0		0.5		0.5		0.5	0	0.5		0		1	0	
170		0	0	1			0	1	0		0.5		0		0.5	0		0	0.5		1	0	
171	0.5	0	0	1			0	0	0		0.5		0.5		0.5	0	0.5	0	0		1	0	
172	0.5	0		1			1	0			1	0.5	0.5	1	0.5	1		0	1	0.5		0	0
173	1	0	1	1			0	0	0		0	0	0	0	0.5	1		0	0	0.5		1	0.5
174		0	0	1			0	0			0		0.5		0.5	0			0.5	0	1	0	
175	0	0		1			0	0			0		0.5		0.5	1			0.5			1	0
176	0.5	0		1			0	0			0.5		0			1			0.5			1	0
177		0	0	1			0	0			0		0.5	0.5		1	0.5		0.5		1	1	0.5
178		0	0	1			0	0			0		0.5	0.5	0.5	0	1	0	0.5		1	1	0.5
179	0.5	0	0	1			1	0			0.5	0.5	0.5	0	0.5	1	1	0	0.5	0.5		1	0.5
180	0	0	0	1			0.5	0			0		0.5	0	0.5	1		0	0.5	0.5		1	0
181		0		1			0	0			0		0.5		0.5	1			0.5	0.5		1	0
182	1	0		1	1	1	1	0			1	0.5	0.5		0.5	1	1	0	0.5	0	1	0	
183	0	0		1			0	0			1		0.5	0.5	1	0			0.5			1	0.5
184		0	0	1			0	0			0		0.5	0.5	0.5	0			0.5			1	0
185	0.5	0	0	1			1	0	0.5		1	0.5	1	1	0.5	0	1	0	0.5	1	1	0	0.5
186		0		1			0	0	0		0		1	0.5	0.5	1	1	0	0.5	1		1	0.5
187		0		1			0	0			0	0.5	0.5		1	1			0.5		0	0	0.5
188		0		1			0	0			0		0.5		0.5	0	0.5		0			0	0.5
189		0		1			0	0			1		0.5	1	0.5	0	0		0.5			0	0.5
190		0	1	1			0	0	0.5		0		0.5	1	0.5	1	1		1		1	1	
191		0		1			0	0			0		0.5		0.5	1			0.5		1	1	
192			1	1			1	1			0	0.5	1	0.5	0.5	1	0.5	0	1	1	1	1	0.5
193		0	1	1			0	1	0.5		0	0.5	1	0.5	0.5	1	0	0	0.5		1	1	0
194		0	1	1			0.5	1	0		0		1	1	0.5	1	0.5	0	1	1	1	0	0.5
195		0		1			0	0					0		0	0			0		1	1	0.5
196		0	1	1			0	0			0		1	0.5	0.5	1	0	0	0		1	1	0.5
197		0	1	1			0	0.5	0		0.5		1	1	0.5	0		0	0.5			1	1
198		0	0	1			0	0			0.5	0.5	1	1	0.5	1	0.5	0	0.5	1	1	1	1
199		0		1			0	0			1		1	0	0	0		0	0.5	0.5	1	1	
200		0		1			0	0	0		0.5		0.5		0.5	0			0.5		1	1	0.5
201		0	1	1			0	0			0		0.5		0	0			0.5		1	1	
202		0		1			0	0	0.5		0	1	1	1	0.5	1	1	0	0.5		1	1	
203		0	1	1			1	1			0		1	0.5	0.5	1		1	0.5		1	0	
204	0.5	1		1	1	1	0	0			0.5	0.5	1	1	1	1	0.5	0	0.5	1		0	0.5
205		0	1	1			1	0	0		1		0.5	1	1		1	0	0.5	1	1	1	0.5

155	1	1	1	1	1	0.5	0	0.5	1	0	1	1	0		1	0.5	0		1	1	1
156	0.5	0	1	0	0.5		0	0.5	1	0	0	0	0		1	0.5	0		0	1	0
157	0.5	0	1	0	1			0.5	1	0.5	0	0	0		1	0.5	0		0	1	0
158	1	1	0.5	1	1	0.5	0	0.5	1	0	0				1	0.5	0		0	1	1
159	1	1	1	1	1		0	0.5	1	0.5	0	0	0		1	0	0		0	1	0
160	1	1	1	0	1		0	0.5	1	0	0	0	1		1	0.5	0.5		1	0	0
161	1	1	1	0	1	1	1	0.5		0	0	0	0		1	1	0		1	0.5	1
162	0	0	0.5	0	0		0	0.5	1	0.5	0	1	1		1	0.5	0		0.5	1	0
163	0	1					0.5	0.5	0.5	0.5	0	0	0		1	0.5	0		0	0	0
164	1	1	1	0.5	1	0.5	0	0.5	1	0.5	0	0	1	0	1	0.5	1		0.5	0.5	0.5
165	1	0	1	0.5	1	0.5	1	1	1	1	0	0	1		1	0.5	0		0	1	0.5
166	1	1		0	1	0.5	0	0.5	1	0.5	0	0	1		1	0.5	1		0	0.5	0.5
167	0.5	0	1	0	1		0	0.5	1	0	0	0	0		1	0.5	1		0.5	1	0
168	1	0	1	0	1	0.5	0	0.5	1	0.5	0	0	0	0	1	0	0		0	1	0
169	0.5	1		0.5	0			0	1	0.5	1	0				1	0	0		0	0.5	0
170	1	0		0	0			0.5	1	1	0	0	1		1	0.5			0	0.5	0
171	0.5	0		0	0			0.5	1	1	0	0	0		1	0.5	0		0	0	0
172	1	0	1	0	1	0.5	0	0.5	1	1	0	0	1		1	0.5	0.5		0	1	0.5
173	1	1	1	0	1	1	0	0.5	1	0	0	0	1		1	0.5	1		1	1	0.5
174	1	0	1	0	1	0.5	0	0.5	1	0	0	0	1		1	0.5	0		0	1	1
175	0	0	1	0			0	0	0.5	0	0	0	0	0	1	0	0		0	0	0
176	1	0	1	0	0.5			0	1	0	0	0.5	0		1	0.5	0		0.5	1	0
177	1	0	1	0	1			0.5	1	1	0	0	0		1	0	0		0	1	0
178	1	0	1	0	1		1	0.5	1	0	0	0	1		1	0			0	1	0
179	1	0	1	0	0		0	0	1	0.5	0	0.5	1		1	0	0		0.5	0.5	0.5
180	1	0	1	0	0		0	0	1	0.5	0	0.5	1	0	1	0	0		0.5	0.5	0.5
181	1	0	1	0	0			0	1	0.5	0	0	0		1	0	0		0.5	0.5	0
182	1	0		1	1		1	0.5	1	0	0	0	1		1	0.5			1	0.5	0
183	0	1		0.5	0		0	0.5	1	0	0	0			1	0	0	1	0	0.5	0
184	0.5	1	1	0	0		0	0.5	1	0	0	0	0	0	1	0	0		0	1	0
185	1	0	1	0	1		0.5	0.5	1	0	0	0	0		1	0	0.5		0	0.5	1
186	0.5	1	1	0.5	1		0	0.5	1	0.5	0	0	0	0	1	0	0		0	0.5	0.5
187	0.5	0	1	0	0		0	0.5	1	0.5	0	0		0	1	0	0	1	0	0.5	0
188	0	0	1	0	0		0	0.5	1	0.5	0	0	0	0	1	0	0	1	0	0	0
189	0	1	1	0.5	0		0	0.5	1	0.5	0	0	0		1	0	0	1	0	0	0.5
190	0.5	1		0			0.5	0.5	1	0	0	0	0		1	0	0	1	0	0	0
191	0.5	1		0	0		0	0.5	1	0	0	0	0		1	0	0		0	0	0
192	1	1		0	1		0	0.5	1	0.5	0	0	1	1	1	1	0.5	1	0	0.5	0
193	0.5	1		0	0		0	0.5	1	0.5	0	0	1	1	1	0	0		0	0.5	0
194	1	1	1	0	0		0	0.5	1	0.5	0	0	1	1	1	0	0.5		0	0.5	1
195	1	0		0	0			0.5	1	0	0	0	0		1	0	0		0	0.5	0
196	0.5	1	1	0	0			0.5	1	0	0	0	0		1	0	0		0	0.5	0.5
197	0.5	1	1	0.5	1			0.5	1	0	0	0	0		1	0.5		1	0	0.5	0.5
198	1	1		0	1			0.5	1	0.5	0	0	0		1	0.5	0		0	0.5	0.5
199	1	1		0	1		0.5	0.5	1	0	0	0	1		1	0.5	0		0	0.5	0.5
200	0.5	0		0	0.5		1	0.5	1	0.5	0	0	0		1	0			0	0.5	0
201	0.5	1		0	0	0.5	0.5	0.5	1	0.5	0	0	0		1	0	0		0	0.5	0.5
202	1	0	1	0.5	0	0.5	0.5	0.5	1	0.5	0	0.5	1		1	0.5	0		0	1	0
203	1	0	1	0	1	0.5	0	0.5	1	0	0	1	0	1	1	0.5	1		1	0.5	1
204	1	0	1	0	1		0	0.5		1	0	0.5	1	0.5		0.5	0		0.5	0.5	1
205	1	1		1	0	0.5	0	0.5	1	1	0	0	1	0.5	1	0	0	1	0.5	0.5	0.5
